# The Effectiveness of Physical Adjunctive Interventions in the Acceleration of Orthodontic Tooth Movement: An Umbrella Review and Meta‐Analysis

**DOI:** 10.1155/ijod/9131541

**Published:** 2026-02-03

**Authors:** Mohamad Radwan Sirri, Mohammad Osama Namera, Mohamad Yaman Salahi Alasbahi, Salar Karim Khalil

**Affiliations:** ^1^ Department of Orthodontics, Damascus University Faculty of Dentistry, Damascus, Syria, damascusuniversity.edu.sy; ^2^ Department of Medical Laboratory Technology, Duhok Polytechnic University, Duhok, Iraq, dpu.edu.krd

**Keywords:** electric stimulation therapy, light-emitting diodes, low-level light therapy, meta-analysis as topic, orthodontic appliances, orthodontics, photobiomodulation therapy, pulsed electromagnetic field therapy, randomized controlled trials as topic, tooth movement, vibration

## Abstract

Orthodontic treatment often lasts around 20 months, and prolonged duration increases the risk of adverse events. Physical adjunctive interventions (PAIs) are proposed to accelerate tooth movement, but their efficacy remains uncertain. This umbrella review synthesized systematic reviews and meta‐analyses of randomized clinical trials (RCTs) on PAIs published through March 2025 (PubMed, Web of Science, Scopus, Cochrane Library). Review quality was appraised with the A Measurement Tool to Assess Systematic Reviews‐2 (AMSTAR‐2) and the Risk of Bias in Systematic Reviews (ROBIS) tools. Study overlap was quantified using the corrected Graphical Representation of Overlap for Overviews (GROOVE) method. Risk of bias in the primary studies was reassessed, when required, using the Cochrane Risk of Bias 2 (RoB 2) tool. Certainty of evidence was rated with the Grading of Recommendations Assessment, Development, and Evaluation (GRADE) approach. Random‐effects models were used when statistical heterogeneity was substantial (*I* ^2^ > 50%); otherwise, fixed‐effects models were used. Effect estimates are reported as mean difference (MD) with 95% confidence intervals (CIs). Seventeen systematic reviews (12 meta‐analyses, 5 narrative) covering 76 RCTs were included. Vibration devices showed minimal or no clinically significant acceleration: leveling/alignment MD = 0.05 mm (95% CI: −0.38 to 0.49), canine retraction + 0.27 mm/month (95% CI: 0.19–0.35), en‐masse retraction + 0.19 mm/month (95% CI: −0.25 to 0.63) (GRADE: moderate–very low). Low‐level laser therapy (LLLT) shortened alignment by −58.4 days (95% CI: −88.6 to −28.2) and, in maxillary extraction, by −28 days (95% CI: −39 to −17), with early canine retraction gains (95% CI: +0.31 to +0.27 mm/month) (GRADE: moderate–very low). Light‐emitting diode (LED) therapy showed + 0.005 mm/day (95% CI: −0.001 to 0.012) with small, non‐significant monthly estimates (GRADE: low–very low). Bioelectric stimulation (BES) added + 1.78 mm at 3 months (95% CI: 0.99–2.57) (GRADE: low). Overall, methodological quality varied widely, with most reviews rated low to critically low by AMSTAR‐2 and at high risk of bias by ROBIS. Vibration should not be the primary accelerator. Photobiomodulation (PBM) is the most promising clinical option, but it requires standardized protocols (wavelength, dose, schedule). BES is promising yet underpowered. Registration: PROSPERO CRD420251043659.

**Summary**

• Photobiomodulation (PBM) accelerates tooth movement by 12.5%–54% and reduces treatment time by 19–58 days.
• Vibrational devices (e.g., AcceleDent) show minimal clinical benefit (<0.3 mm/month).
• Pulsed electromagnetic field therapy (PEMF) boosts canine retraction by 1.78 mm in 3 months (*p*  < 0.0001).
• PBM effectiveness is higher in the mandible (1.03 mm) than maxilla (0.33 mm).
• Evidence quality is low (AMSTAR‐2/ROBIS); standardized protocols and rigorous trials are needed.

## 1. Introduction

Orthodontic treatment typically lasts 14–33 months (mean: 19.9 months) and requires roughly 18 clinical visits [1]. Treatment duration varies with clinician decisions, case complexity, patient adherence, and the use of acceleration strategies, which are commonly classified as surgical, pharmacological, or physical [2, 3].

Shortening treatment is desirable because it improves comfort and adherence and reduces complications such as root resorption, demineralization, and periodontal disease [4]. However, surgical acceleration may introduce adverse effects, including interdental bone loss [5], loss of tooth vitality [6], surgical scarring [7], gingival recession [8], mechanical root injury [9], subcutaneous hematomas [10], and bacteremia [11].

Therefore, developing nonsurgical adjunctive interventions is essential to deliver accelerated orthodontic treatment that mitigates current complications and avoids introducing new adverse effects.

In orthodontics, physical adjunctive interventions (PAIs) are classified by the type of energy used to stimulate tissues and cells. Mechanical energy includes low‐intensity pulsed ultrasound (LIPUS), which generates gentle acoustic waves that induce mechanical stress and promote bone remodeling [12], and mechanical vibrations (MVs) at low or high frequencies, which activate cells by stressing the periodontal ligament [13]. Light‐based approaches, such as low‐level laser therapy (LLLT) and light‐emitting devices (LEDs), act via photobiomodulation (PBM) to increase mitochondrial ATP production, enhance osteoblastic activity, and trigger controlled sterile inflammation [14]. Bioelectric stimulation (BES) includes methods that use electrical or electromagnetic energy to modulate cellular activity. These include low‐intensity electrical stimulation (LIES), which applies mild currents to enhance signaling (e.g., upregulating RANKL to stimulate bone remodeling) [15], and pulsed electromagnetic fields (PEMF), which alter electrochemical signaling and regulate ion fluxes (e.g., calcium) to activate bone cells [16]. Despite the different energy sources, these modalities converge on intra‐ and extracellular pathways that increase blood flow and RANKL expression, thereby accelerating tooth movement via the regional acceleratory phenomenon [4, 13, 17].

Although marketing frequently claims meaningful time savings and fewer side effects, the clinical evidence from human‐based randomized controlled trials (RCTs), systematic reviews, and meta‐analyses remains inconclusive [18–20]. Marked heterogeneity in intervention types (vibration, PBM, electrical stimulation), treatment mechanics (canine retraction, leveling and alignment, En‐masse retraction; fixed appliances vs clear aligners), outcome measurements (e.g., Little’s Irregularity Index, monthly millimetric progress; direct vs model‐based assessments), and primary study quality has produced inconsistent findings across reviews of adjunct physical interventions for accelerating orthodontic tooth movement (OTM) [4, 16, 21, 22]. Some studies report increased tooth‐movement rates [23–25], whereas others show no significant difference [26–28]. These discrepancies are compounded by methodological issues, such as pooling diverse interventions within a single analysis and repeatedly including the same trials across multiple reviews [23, 27]. These practices reduce specificity, create redundancy, and make it difficult for clinicians to interpret the evidence and select effective techniques.

This umbrella review critically appraises the evidence on PAIs for accelerating OTM by systematically integrating data from existing systematic reviews and meta‐analyses. The umbrella‐review approach is uniquely suited to map overlap among prior syntheses, compare their methodological quality, and reconcile conflicting conclusions across interventions and outcomes. By synthesizing available data, it clarifies methodological sources of heterogeneity, estimates the true efficacy of each modality, and offers evidence‐based clinical guidance. It also counters unsupported marketing claims, identifies research gaps that warrant rigorous future trials, and aims to strengthen confidence in clinical decision‐making.

To the best of current knowledge, this work represents the first comprehensive umbrella review synthesizing evidence on PAIs designed to accelerate orthodontic tooth movement.

## 2. Materials and Methods

### 2.1. Scoping Search

This umbrella review followed a pre‐registered protocol (PROSPERO ID: CRD420251043659). A preliminary PubMed search confirmed no prior umbrella reviews addressing the research objective. The protocol was developed by Preferred Reporting Items for SRs and Meta‐Analyses (PRISMA) and the Cochrane guidelines for systematic reviews.

### 2.2. Eligibility Criteria

Review question: What is the impact of PAIs on OTM acceleration among patients enrolled in randomized clinical trials (RCTs)?

Inclusion criteria were defined using the PICOS framework:

• Participants: Healthy individuals of any age, gender, or ethnicity undergoing orthodontic treatment.
• Interventions: PAIs for accelerating OTM, for example, MV, PBM, BES.
• Comparisons: Control groups receiving standard orthodontic treatment without adjunctive interventions.
• Outcomes: Rate of tooth displacement (e.g., mm/week/month) or equivalent efficacy metrics.
• Study Design: Systematic reviews/meta‐analyses of RCTs (parallel or split‐mouth designs).

Exclusion Criteria:

• Systematic reviews and meta‐analyses, including those of clinical trials, cohort studies, narrative reviews, case series/reports, or animal research.
• Studies of PAIs that assess pain, root resorption, or other adverse events as primary outcomes.
• Comparative studies of PAIs versus alternative acceleration techniques (surgical or nonsurgical interventions, medical approaches, or other methods).
• Systematic reviews, meta‐analyses, or primary studies lacking comprehensive or retrievable data.

### 2.3. Search Strategy

A systematic search was conducted across PubMed, Web of Science, Scopus, and the Cochrane Library up to March 2025. Controlled vocabulary (MeSH and related free‐text words) and tailored keywords were combined and optimized for each database. To address publication bias, gray literature (e.g., theses) was sourced through ProQuest Dissertations and archived records from OpenGrey Europe (via DANS EASY). Manual searches included reference lists of relevant studies and four key orthodontic journals (*American Journal of Orthodontics and Dentofacial Orthopedics*, *European Journal of Orthodontics*, *Journal of Orthodontics*, and *Journal of Orthodontics and Craniofacial Research*). No restrictions were applied to language, publication date, or status. Full search syntax is provided in Supporting Information 1: Table S1.

### 2.4. Study Selection, Data Extraction Process, and Overlap Assessment

Two reviewers (MRS and MON) independently screened titles and abstracts to identify potentially eligible reports, with full texts assessed against predefined inclusion criteria; any discrepancies were resolved through consultation with a third reviewer (MYSA).

For the SRs, data were extracted via a standardized form capturing publication details (year, authors, study design, sample size, and age range), search characteristics (registration status, strategy, restrictions, and execution date), methodological quality (risk‐of‐bias tool and GRADE metrics), intervention and comparator parameters (device type, frequency, force, duration, treatment, and appliance specifics), outcomes (primary endpoints, measurement tools, follow‐up intervals, and results), and ethics/funding information (sources and conflicts of interest).

For the primary studies, citation matrices were generated to quantify overlap [29] and corrected covered areas (CCAs) calculated using the Graphical Representation of Overlap for Overviews tool (GROOVE) [30] (as detailed in Supporting Information 2: Table S2). Subsequently, data were extracted from these de‐duplicated studies on bibliographic details, sample size, study design, orthodontic intervention type, PAIs and parameters, inclusion frequency in systematic reviews, bias‐assessment method, and resulting bias scores.

### 2.5. Methodological Quality and Risk‐of‐Bias Assessment in Selected Systematic Reviews and Their Primary Studies

The methodological quality of included systematic reviews was assessed with A Measurement Tool to Assess Systematic Reviews‐2 (AMSTAR‐2, 16 domains: 7 critical, 9 noncritical), and their risk of bias was evaluated using the Risk of Bias in Systematic reviews tool (ROBIS, three phases: Assessing relevance, identifying concerns with the review process, and judging risk of bias). Details of the questions, response options, and rating criteria for both tools are presented in Supporting Information 2: Table S2.

For primary studies, initial bias assessment was based on the results of the highest‐confidence reviews (high confidence according to AMSTAR‐2 and low risk according to ROBIS), followed by a reassessment with the Cochrane Risk of Bias 2 (RoB 2) tool under the following exceptional circumstances:

1. A review was rated “moderate,” “low,” or “critically low” in AMSTAR‐2 or identified as “unclear” or “high” risk by ROBIS.
2. Conflicting bias judgments existed for the same study between two or more reviews.
3. Reviews published before 2019 or after 2019 continued to use RoB 1 rather than upgrading to RoB 2.

All assessments (AMSTAR‐2, ROBIS, and RoB 2) were conducted independently by two reviewers (MRS and MON), with any discrepancies resolved by a third reviewer (MYSA) to ensure a unified final classification.

### 2.6. Evaluating the Strength of Evidence

The evidence quality was assessed using the Grading of Recommendations Assessment, Development, and Evaluation (GRADE) framework. Subgroup analyses were performed for each PAI to evaluate their effects separately.

Evidence quality was classified into four levels (high, moderate, low, or very low). Two independent reviewers (MRS and MON) performed the assessments, and disagreements were resolved through consultation with a third reviewer (MYSA).

### 2.7. Primary Studies Pooling and Meta‐Analysis

Similar outcomes were combined quantitatively in Review Manager (version 5.4.1; The Nordic Cochrane Centre, Copenhagen, Denmark). Continuous results were expressed as mean differences (MDs; 95% CIs), and heterogeneity was assessed using Tau^2^ and *I* ^2^ statistics. A random‐effects model was applied when *I* ^2^ exceeded 50%, with a fixed‐effects model used otherwise. Clinically heterogeneous outcomes were narratively synthesized.

Publication bias was planned to be assessed through visual inspection of contour‐enhanced funnel plots if a sufficient number of trials examining the same intervention and outcome were available.

All data collection, analysis, and presentation of results were conducted by one reviewer (MYSA) and independently checked by a second (MRS).

## 3. Result

### 3.1. Search Results

**Figure 1:**
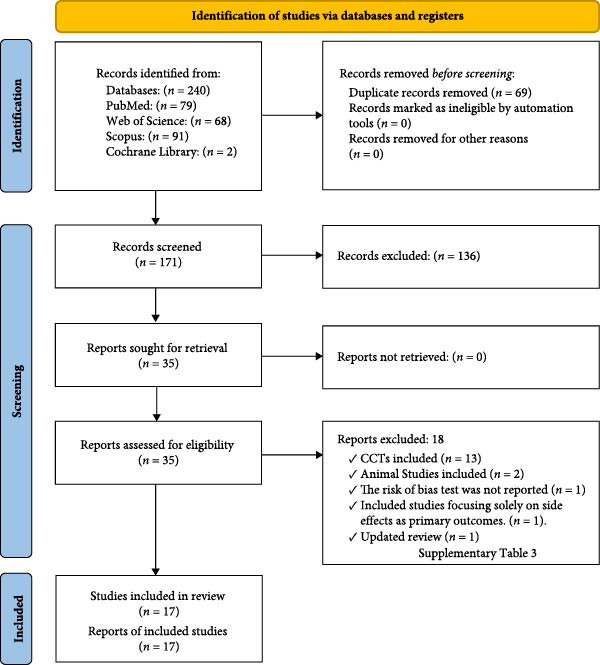
Preferred reporting items for systematic reviews and meta‐analyses (PRISMA) flow chart for included systematic reviews.

A total of 240 articles were identified through database searches. After 69 duplicates were removed, 171 records underwent title and abstract screening, which excluded 136 articles as irrelevant. The remaining 35 full‐text articles were assessed for eligibility, with 18 excluded for not meeting the predefined inclusion criteria. Ultimately, 17 systematic reviews were included in the final analysis. The literature search process is summarized in the flow diagram (Figure 1). Excluded studies after full‐text review, with reasons, are listed in Supporting Information 3: Table S3.

### 3.2. Characteristics and Findings of the Included Systematic Reviews

This synthesis analyzed 17 SRs published between 2016 and 2025, including 12 quantitative (meta‐analytic) and five qualitative (narrative) reviews. The quantitative reviews examined four distinct interventions: General Physical Acceleration (one review [4]), BES (one review [16]), Vibration Devices (six reviews [13, 22, 23, 26–28]), and PBM (LLLT and LED) (nine reviews [14, 21, 24, 25, 31–35]). All reviews included only RCTs, with five employing the Cochrane ROB2 tool for bias assessment [13, 16, 21, 34, 35] and nine using GRADE for evidence quality evaluation [4, 13, 16, 23, 28, 32–35].

Thematic analysis revealed that most SRs addressed multiple orthodontic approaches. Leveling and alignment were covered in 13 reviews [4, 13, 14, 16, 22, 23, 26–28, 32–35], canine retraction in all 17, En‐masse retraction in 10 [4, 13, 21–23, 25, 27, 28, 32, 33], molar intrusion in one [4], and premolar retraction in one [25]. Table 1 summarizes the characteristics of the Included Systematic Reviews.

**Table 1:** Characteristics of the included systematic reviews on acceleration of OTM using the physical adjunctive interventions.

Intervention type	General information					Study identification and search			Methodological quality	Interventions and comparisons			
	Study ID/author, year, country	Study design	No. of trials and design	Age of participants	No. of participants	Registration status	Search strategy/restrictions	Date of search	ROB assessment tool/quality of evidence	Device	Specifications VD: Frequency/force/daily use PBMD: WL (nm)/IP/time SE/ED (J/cm²)/ OP (mW) BES: Parameters/duration/Location	Type of orthodontic treatment	Additional details
General physical adjunctive interventions	El‐Angbawi et al. [4], UK	SR/MA	–23 RCTs (parallel) –13 in MA: 1. LVF analysis: ✓ Overall orthodontic treatment duration ✓ Number of appliance adjustment visits ✓ Tooth movement rate during early alignment (4–6 weeks) ✓ Rate of space closure (En masse) 2. LLLT analysis: ✓ Alignment stage duration ✓ Number of visits during alignment ✓ Tooth movement rate during space closure (maxillary arch) 3. LED analysis: ✓ Alignment stage duration ✓ Tooth movement rate during space closure	8–50 years	Total: 1027 – VD: – SD: – LLLT: – PLA: – Con:	Cochrane CD010887	Electronic databases (5) + manual search No restriction for language	Up to September 2022	ROB1 UROB: 2 RCTs HROB: 22 RCTs GRADE: LQE VLQE	AcceleDent (9 RCT)	30 Hz/0.25 N/20 min/day	LADC without EX: 2 RCTs LADC without EX: 1 RCT DR without EX: 3 RCT UCR: 3 RCTs	Used with FA and CA
										Tooth Masseuse (1 RCT)	111 Hz/0.06 N/20 min/day	LADC without EX: 1 RCTs	
										Tooth Bruch (1 RCT)	30–60–120 Hz/0.001 N/20 min/day	UCR: 1 RCTs	
										Customized vibratory appliance (1 RCT)	30 Hz/0.2 N/20 min/day	ER: 1 RCT	
										LLLT (10 RCT)	635–980 nm/1–10 points/3–10 s per point Daily‐every 15–30 days SE/2.25–25.7 j/cm^2^/8–2500 mW	LADC without EX: 5 RCTs UADC with EX: 2 RCTs ER: 1 RCT UI: 2 RCTs	
										LED (2 RCTs)	640–850 nm/intraoral/5–20 min per day Daily SE/10–108 j/cm^2^/40–90 mW/cm^2^	LADC without EX: 1 RCTs UCR: 1 RCT	

BES	Dutta et al. [16], India	SR/MA	– 4 RCTs (3 SMD/1 parallel) – 2 of them in MA for the RCR in the third and fifth months	18–24 years	Total: 78 – BES – SD –Con	Pro CRD42023495077	Electronic databases (4) + manual search Unclear restriction for language	Up to November 2023	ROB2 LROB: 1 RCT HROB: 3 RCTs GRADE: MQE	LIDEC (1 RCT/ SMD)	20 μA (15 V)/5 h/day Upper canines	UCR: 1 RCT	Used with FA
										PEMF (2 RCTs/ SMD)	0.5 mT, 1 Hz/8 h/night Upper canines	UCR: 2 RCTs	
										BM (1 RCT/Parallel)	10 μA/5 min/week Lower incisors	LADR: 1 RCT	

Vibration devices	Aljabaa et al.[26], Saudi Arabia/USA	SR	– 6 RCTs (parallel)	11.1–40 years	Total: 339 – VD – SD – Con	Not registered	Electronic databases (3) + manual search No restriction for language	Up to March 2018	ROB 1: ROB: 4 RCTs HROB: 2 RCTs	AccelDent 5RCTs	30 Hz/0.2 N/20 min/day	– UCR: 1 RCT ER: 1 RCT – LADC with EX: 1 RCT – LADC without EX: 1 RCT – DR without EX: 1 RCT	– Used with FA (5 RCTs) and CA (1 RCT)
										Tooth Masseuse 1RCT	111 Hz/0.06 N/20 min/day	– LADC without EX: 1 RCT	
	Abd Elmotaleb et al. [27], Egypt	SR/MA	– 6 RCTs (parallel) – 3 of them in MA (LII)	11–40 years	Total: 232– VD – SD – Con	At the Evidence‐Based Center, in their faculty (Registration number: 14–2018)	Electronic databases (3) + manual search English only	Up to July 2018	ROB 1: LROB: 2 RCTs HROB: 4 RCTs	AcceleDent 5 RCTs	30 Hz/0.2 N/20 min/day	– LADC with EX: 1 RCT – LADC without EX: 1 RCT – UCR: 1 RCT ER: 2 RCTs	– Used with FA (6 RCTs)
										Tooth Masseuse 1RCT	111 Hz/0.06 N/20 min/day	– LADC without EX: 1 RCT	
	Bakdach et al. [22], Syria	SR	– 17 RCTs (14 parallel/ 3 SMD)	12–33 years	Total: 730– VD – SD – Con	Not registered	Electronic databases (7) + manual search No restriction for language	Up to November 2019	ROB 1:LROB 5 RCTs UROB: 11 RCTs HROB: 1 RCT	AcceleDent 12 RCTs	30 Hz/20 min/day	– LADC with EX: 1 RCT – LADC without EX: 2 RCTs – DR without EX: 5 RCT – UCR: 2 RCTs – ER: 2 RCTs	– Used with FA (12 RCTs) and CA (5 RCTs)
										Electric Toothbrush 3 RCTs	125 Hz/20 min/day 125 Hz/15 min/day 113 Hz/10 min/day	– UCR: 3 RCTs	
										VPro5 1 RCT	120 Hz/5 min/day	– LADC without EX: 1 RCT	
										Tooth Masseuse 1 RCT	111 Hz/20 min/day	– LADC without EX: 1 RCT	
	Keerthana et al. [23], India	SR/MA	– 12 RCTs (9 parallel/3 SMD) – 8 of them in MA (RTM)	12–40 years	Total: 526– VD – SD – Con	PROSPERO CRD42020169675	Electronic databases (3) + manual search English only	Up to Jan 31, 2020	ROB 1: UROB: 2 RCTs HROB: 8 RCTs Grade: MQE: (9 RCTs) HQE: (1 RCT)	AcceleDent 7 RCTs	30 Hz/0.2 N/20 min/day	– LADC with EX: 1 RCT – LADC without EX: 1 RCT – DR without EX: 1 RCT – UCR: 2 RCTs – ER: 2 RCTs	– Used with FA (11 RCTs) and CA (1 RCTs)
										Electric toothbrush 4 RCTs	50 Hz/0.2 N/10 min/day 30 Hz–60 Hz–120 Hz/0.001 N/20 min/day 100–105 Hz/5 min/day	– UCR: 4 RCTs	
										Tooth Masseuse 1 RCT	30 Hz/0.2 N/20 min/day	– LADC without EX: 1 RCT	
	García Vega et al. [28], Mexico	SR	– 15 RCTs (11 parallel/ 4 SMD)	11–45 years	Total: 670– VD – SD – Con	PROSPERO CRD42021245217	Electronic databases (6) + manual search English only	From 2010 to June 2021	ROB 1: LROB: 3 RCTs UROB: 5 RCTs HROB: 7 RCT Grade: LQE: 15 RCTs	AcceleDent 7 RCTs	30 Hz/0.2 N/20 min/day	– LADC with EX: 1 RCTs – LADC without EX: 1 RCTs – DR without EX: 2 RCT – UCR: 2 RCTs – ER: 1 RCTs	– Used with FA (12 RCTs) and CA (3 RCTs)
										Electric Toothbrush 5 RCTs	50 Hz/0.2 N/10 min/day 30 Hz–60 Hz–120 Hz/0.001 N/20 min/day 100–105 Hz/5 min/day 125 Hz/5 min/day	– UCR: 5 RCTs	
										VPro5 1 RCT	120 Hz/0.0003 N/5 min/day	– LADC without EX: 1 RCT	
										Tooth Masseuse 1 RCT	111 Hz/0.06 N/20 min/day	– LADC without EX: 1 RCTs	
										Customized appliance 1 RCTs	30 Hz/0.2 N/20 min/day	– ER: 1 RCTs	
	Dutta et al. [13], India	SR/MA	– 21 RCTs (14 parallel/ 7 SMD) – 14 of them in MA (5 for LII / 6 for CRR	12–40 years	Total: 815 – VD – SD – Con	PROSPERO CRD42024542014	Electronic databases (5) + manual search No restriction for language	Up to April 2024	ROB 2: LROB: 3 RCTs SROB: 18 RCTs Grade: MQE: 14 RCTs	AcceleDent 9 RCTs	30 Hz/0.2 N/20 min/day	– LADC with EX: 1 RCTs – LADC without EX: 2 RCTs – DR without EX: 1 RCT – UCR: 3 RCTs – ER: 2 RCTs	– Used with FA (19 RCTs) and CA (2 RCTs)
										Electric Toothbrush 5 RCTs	50 Hz/0.2 N/10 min/day 30 Hz–60 Hz–120 Hz/0.001 N/20 min/day 100–105 Hz/5 min/day 125 Hz/5 min/day	– UCR: 5 RCTs	
										VPro5 1 RCT	120 Hz/0.0003 N/5 min/day	– LADC without EX: 1 RCT	
										Tooth Masseuse 1 RCT	111 Hz/0.06 N/20 min/day	– LADC without EX: 1 RCTs	
										Customized appliance 5 RCTs	30 Hz/0.2 N/20 min/day 102 Hz/0.05 N/3 min/visit 125–150 Hz/1 min/tooth	– LADC without EX: 1 RCTs – UCR: 2 RCTs – ER: 2 RCTs	

Photobiomodulation	Almeida et al. [31], Brazil	SR/MA	– 6 RCTs (SMD) – 5 of them in MA for the CRR	10.5–23 years	Total: 73 – LLLT – PLA – Con	PROSPERO CRD42015025009	Electronic databases (6) No restriction for language	Up to September 2015	Selection criteria and scores, adapted from Cericato et al. MQ: 1 RCT HQ: 5 RCTs	LLLT 6 RCTs (6 SMD) GaAlAs: 5 RCTs GaAs: 1 RCT	NA/3–5 points/NA 3–8 SE/4.2–10 (J/cm²)/NA	UCR: 3 RCTs U&LCR: 3 RCTs	– Used with FA
	Imani et al. [24], Iran	SR/MA	– 6 RCTs (SMD) – 6 in MA for the CRR	10.5–31 years	Total: 96 – LLLT – PLA – Con	Not registered	Electronic databases (5) No restriction for language	Up to October 2017	ROB 1: UROB: 5 RCTs HROB: 1 RCT	LLLT 6 RCTs (6 SMD) GaAlAs	780–940 nm/NA/3–10 s per point 3–17 SE/5–8 (J/cm² per point)/20–200 mW	UCR: 4 RCTs U&LCR: 2 RCTs	– Used with FA
	Deana et al. [32], Chile	SR/MA	16 RCTs (2 parallel/ 14 SMD) – 12 of them in MA for the CRR per month/Accumulated OTM rate	10.5–31 years	Total: 273 – LLLT – PLA – Con	Not registered	Electronic databases (4) + manual search Language restrictions (English/Spanish/Portuguese)	Up to March 2018	ROB 1: UROB: 9 RCTs HROB: 7 RCTs Grade: VLQE/LQE/MQE	LLLT 16 RCTs (2 parallel/14 SMD) GaAlAs: 15 RCTs GaAs: 1 RCTs	780–980 nm/4–10 points/3–30 s per point 1–17 SE/33–214 (J/cm²)/0.25–200 mW	UCR: 8 RCTs U&LCR: 5 RCTs – UADC with EX: 1 RCTs – LADC without EX: 1 RCTs – ER: 1 RCTs	– Used with FA
	Bakdach et al. [33], Syria	SR/MA	25 RCTs (7 parallel/18 SMD) – 17 MA studies: upper CR/month; 5 included lower canines	12–40 years	Total: 570 – LLLT – PLA – Con	Not registered	Electronic databases (9) + manual search No restriction for lg	Up to June 2019	ROB 1: LROB: 4 RCTs UROB: 17 RCTs HROB: 4 RCTs Grade: LQE/VLQE	LLLT 20 RCTs (3 Parallel/17 SMD) GaAlAs: 19 RCTs GaAs: 1 RCTs	780–980 nm/4–10 points/3–30 s per point 3–17 SE per month/2.25–150 (J/cm²)/0.25–1000 mW	– LADC without EX: 1 RCTs. – UADC with EX: 1 RCTs – DR without EX: 1 RCT UCR: 10 RCTs U&LCR: 7 RCTs	– Used with FA (24 RCTs) and CA (1 RCTs)
										LED 5 RCTs (4 parallel/1SMD)	618–850 nm/NA (extra ‐intra oral)/3–20 min per session 21–180 SE/ 0.063–108 (J/cm²)/20–90 mW/cm²	– LADC without EX: 1 RCTs – UADC with EX: 1 RCTs – DR without EX: 1 RCT UCR: 1 RCTs – ER: 1 RCTs	
	Camacho et al. [25], Colombia	SR	9 RCTs (1 parallel/8 SMD)	12–35 years	Total: 126– LLLT – PLA – Con	PROSPERO CRD42019117648	Electronic databases (4) + manual search English only	From January 2001 to February 2018	ROB 1: UROB: 2 RCTs HROB: 7 RCTs	LLLT 9 RCTs (1 Parallel/8 SMD)	670–904 nm/NA/NA 2–9 SE/NA/12–200 mW	UCR: 3 RCTs U&LCR: 3 RCTs – UADC with EX: 1 RCTs – UPR: 1 RCTs – ER: 1 RCTs	– Used with FA
	Grajales et al. [21], Spain	SR/MA	19 RCTs (1 parallel/18 SMD) – 18 of them in MA: UCRR/month Linking WL and energy to accelerated OTM	10–35 years	Total: 311 – LLLT – PLA – Con	PROSPERO CRD42022332585	Electronic databases (4) + manual search No restriction for language	Up to October 2022	ROB 2: LROB: 2 RCTs SROB: 17 RCTs	LLLT 19 RCTs (1 parallel/18 SMD)	658–980 nm/4–10 points/3–23 s per point 3–17 SE/4.2–25 (J/cm²)/NA	UCR: 10 RCTs U&LCR: 7 RCTs – ER: 2 RCTs	– Used with FA
	Jnaneshwar et al. [34], India	SR/MA	10 RCTs (3 Parallel/7 SMD) – 6 of them in MA (4 UCRR/2 Anterior Alignment)	12–24 years	Total: 223 – LLLT – PLA – Con	PROSPERO CRD42020196472	Electronic databases (5) + manual search No restriction for language	Up to June 2020	ROB 1: LROB: 3 RCTs UROB: 7 RCTs GRADE: HQE MQE	LLLT 8 RCTs (2 Parallel/6 SMD)	808–980 nm/4–10 points/10–184 s per point or tooth 3–15 SE/7.5–150 (J/cm²)/20–1000 mW	– LADC without EX: 1 RCTs. – UADC with EX: 1 RCTs. U&LCR: 2 RCTs UCR: 4 RCTs	– Used with FA
										LED 2 RCTs (1 parallel/1 SMD)	618–850 nm/NA (extra–intra oral)/20 min per day Daily SE/150 (J/cm²)/20–90 (mW/cm²)	– LADC without EX: 1 RCTs. UCR: 1 RCTs	
	Malik et al. [14], India	SR/MA	6 RCTs (4 Parallel/2 SMD) – 2 of them in MA: UCRR/month	Not available	Total: NA – LLLT – PLA – Con	Not registered	Electronic databases (5) English only	Not available	ROB 1: LROB: 4 RCTs HROB: 2 RCTs	LLLT 4 RCTs (2 Parallel/2 SMD)	810–980 nm/4–6 points/8–30 s per point Variable SE (monthly to daily)/5–150 (J/cm²)/100–1000 mW	– LADC without EX: 1 RCTs – UADC with EX: 1 RCTs U&LCR: 2 RCTs	– Used with FA
										LED 2 RCTs (2 parallel)	850 nm/NA (extra–intra oral)/5–20 min per day Daily/0.065–108 (J/cm²)/65 mW–90 (mW/cm²)	– LADC without EX: 1 RCTs – UADC without EX: 1 RCTs	
	Hmida et al. [35], Tunisia	SR	14 RCTs (5 parallel/9 SMD)	Mean age 13.4–21.5 years	Total: 416 – LLLT – PLA – Con	Not registered	Electronic databases (3) + manual search English only	Up to October 15, 2023	ROB 2: LROB: 10 RCTs SROB: 4 RCTs GRADE: HQE: 6 RCTs/ MQE: 8 RCTs	LLLT 11 RCTs (11 SMD)	810–980 nm/4–10 points/3 s per point‐80 s per aspect 3–16 SE/25.7–150 (J/cm^2^)/100 mW	LADR without EX:1 RCT UADR with EX: 1 RCT DR without EX:1 RCT UCR: 6 RCTs U&LCR: 1 RCT UER: 1 RCT	– Used with FA
										LED 3 RCTs (3 SMD)	450–850 nm/NA (extra‐intra oral)/10–20 min per day Daily‐Biweekly SE/36–144 (J/cm2/session)/60 mW/cm²	LADR: 2 RCTs UCR: 1 RCT	

*Note:* SRs were organized using physical acceleration method (General Physical Adjunctive Intervention, BES, vibration, PBM) and sorted chronologically (oldest to newest) within each category.

Abbreviations: BES, bioelectric stimulation; BM, bimaxillary mouthpiece; cm, centimeter; Con, Control group; CRR, Canine Retraction Rate; DR, decrowding; ED, energy density; ER, En masse retraction; GRADE, Grading of Recommendations, Assessment, Development and Evaluation; HROB, high risk of bias; HQE, high‐quality evidence; Hz, Hertz; IP, irradiation points; J, Joule; LADR, lower anterior decrowding; LED, light‐emitting diode; LIDEC, low‐intensity direct current; LII, little irregularity index; LLLT, low level laser therapy; LQE, low‐quality evidence; LROB, low risk of bias; LVF, light vibrational forces; MA, meta‐analysis; Min, minute; MQE, moderate quality evidence; mW, milliwatt; N, Newton; OP, output power; PBM, photobiomodulation; PEMF, pulsed electromagnetic field; PLA, Placebo; RCT, randomized clinical trials; ROB, risk of bias; RTM, rate tooth movement; SD, sham device group; SE, sessions; SMD, split mouth design; SR, systematic review; UADC, upper anterior decrowding; UCR, upper canine retraction; UI, upper intrusion; UROB, unclear risk of bias; VD, vibration device; VLQE, very low‐quality evidence; WL, wavelength.

Findings on vibrational devices were inconsistent: while Abd Elmotaleb et al. [27] found no significant improvement in Little’s Index (*p* = 0.49), Keerthana et al. [23] reported a statistically significant overall acceleration in tooth movement (*p*  < 0.00001). Dutta et al. [13] documented substantial acceleration in canine retraction (standardized mean difference [SMD] = 2.48, *p*  < 0.05), despite considerable heterogeneity. Descriptive data further suggested that high‐frequency vibrations (113–125 Hz) were more effective, while low‐frequency vibrations produced negligible results (Table 2).

**Table 2:** Outcomes of the included systematic reviews on acceleration of OTM using the physical adjunctive interventions.

Intervention type	Study ID/author, year, country	Outcomes					Ethics and funding	
		Primary outcomes	Outcome measurement tools	Follow‐up period	MA reported outcomes	Descriptive reported outcomes	Source of funding	Declared conflicts of interest
General physical adjunctive interventions	El‐Angbawi et al. [4], UK	✓ Overall orthodontic treatment duration ✓ Number of appliance adjustment visits ✓ Rate of tooth movement during treatment phases (alignment, space closure)	✓ Time documentation (in months) ✓ Clinic records ✓ LII for malalignment measurement ✓ SC: mm per month on study models	✓ Duration: VD: 4–16 weeks LLLT: 6–12 weeks LED: 3–6 months ✓ Frequency: VA: 4–6 weeks LLLT: every 2 weeks, 4 per month LED: 20 min daily ✓ Focus: Short‐term effects only	Light Vibrational Forces (LVF) Analysis: 1. Overall orthodontic treatment duration (2 RCTs/77 PT): ◦ No significant difference found (MD: −0.61 months; 95% CI: −2.44 to 1.22) 2. Number of appliance adjustment visits (2 RCTs/77 PT): ◦ No reduction observed (MD: −0.32 visits; 95% CI: −1.69 to 1.05) 3. Tooth movement rate during early alignment (4–6 weeks) (3 RCTs/144 PT): ◦ No acceleration detected (MD: 0.12 mm; 95% CI: −1.77 to 2.01) 4. Rate of space closure (En masse) (2 RCTs/81 PT): ◦ Minimal clinical effect (MD: 0.10 mm/month; 95% CI: −0.08 to 0.29) 5. Rate of canine retraction = (2 RCTs/40 PT): ◦ No acceleration detected (MD: −0.01 mm/month; 95% CI: −0.20 to 0.18) Low‐Level Laser Therapy (LLLT) Analysis: 1. Alignment stage duration (3 RCTs/92 PT): ◦ Statistically significant reduction (MD: −48.87 days; 95% CI: −56.48 to −41.26) 2. Number of visits during alignment (2 RCTs/125 PT): ◦ Reduction observed (MD: −2.25 visits; 95% CI: −2.52 to −1.97) Light‐Emitting Diode (LED) Analysis: 1. Alignment stage duration (1 RCTs/34 PT): ◦ Reduction observed (MD: −24.5 days; 95% CI: −42.45 to −6.55) 2. Tooth movement rate during space closure (1 RCTs/39 PT): ◦ No significant difference (MD: 0.006 mm/day; 95% CI: 0 to 0.02)	—	SR: NF PS: Katchooi et al. [36], Lombardo et al. [37], Pavlin et al. [38], Reiss et al. [39] Funded by OrthoAccel Technologies (manufacturer of vibrational devices like Acceledent).	SR: NC PS: Funding from OrthoAccel Technologies creates a potential conflict of interest (COI) in the cited studies (Katchooi et al. [36], Lombardo et al. [37], Pavlin et al. [38], Reiss et al. [39])

BES	Dutta et al. [16], India	UCR (mm/month) Decrowding DR	✓ RCR: Digital calipers (millimeter measurements between teeth/anchors) ✓ LII (crowding severity scoring)	✓ Duration: Ranged from 4 weeks to 5 months One study continued until treatment completion ✓ Frequency: Weekly Monthly Pre‐ and post‐treatment only ✓ Focus: Short‐term effects only	• Positive correlation between electrical stimulation and accelerated orthodontic tooth movement (30–40% faster movement in some studies) • Meta‐analysis results: – 3rd month (2 RCTs/29 PT): Standardized Mean Difference (SMD) = 0.69 (95% CI: −1.26 to 0.12), *p* < 0.05 (statistically significant) – 5th month (2 RCTs/29 PT): SMD = 1.64 (95% CI: −3.44 to 0.16), *p* > 0.05 (nonsignificant)	—	NF	NC

Vibration devices	Aljabaa et al. [26], Saudi Arabia/USA	RTM DR/CR/SC	✓ LII On study models by Digital caliper ✓ RCR: mm per month, Direct in the patient’s mouth by Digital caliper ✓ SC: mm per month on study models by Digital caliper	✓ Initial alignment studies: 8 weeks–7 months. ✓ CRR: until canine retraction ✓ SC: 10 weeks–6 months	—	• Tooth Movement: 1/6 studies (Pavlin et al. [38]) reported an increased rate 5/6 studies found no significant difference • Pain: No reduction in 3/3 studies (except reduced analgesic use in Miles & Fisher)	SR: NF PS: ✓ Pavlin et al. [38]: Funded by OrthoAccel Technologies ✓ Katchooi et al. [36]: Funded by OrthoAccel Technologies ✓ Woodhouse et al. [40]: Received devices from OrthoAccel Technologies	SR: NC PS: ✓ Pavlin et al. [38]: The lead author was a paid consultant for OrthoAccel during the study’s development and reporting ✓ Katchooi et al. [36]: The study’s risk of bias was rated as "unclear" due to funding from the device manufacturer ✓ Woodhouse et al. [40]: The Authors declared no financial conflicts of interest, despite receiving free devices
	Abd Elmotaleb et al. [27], Egypt	RTM CR/DR/SC	✓ LII On study models by Digital caliper ✓ RCR: mm per month, directly in the patient’s mouth by digital caliper. ✓ SC: mm per month on study models by Digital caliper	10–18 weeks (short‐term)	• LII (3 RCTs/162 PT): no significant difference between vibration and control groups (MD: −0.24 mm, 95% CI: −0.92 to 0.45, *p* = 0.49)	—	SR: NM PS: Pavlin et al. [38]: Funded by OrthoAccel Technologies	SR: NC PS: Pavlin et al. [38]: The authors of this study did not explicitly declare conflicts in the review
	Bakdach et al. [22], Syria	RTM DR/CR/SC	✓ LII On plaster models by digital caliper or on digital models ✓ PCPD ✓ Tracking Percentage ✓ RCR: mm per month, Direct in the patient’s mouth or on plaster models by Digital caliper ✓ SC: mm per month on study models	✓ Short‐Term: 3–12 weeks (e.g., canine retraction studies). ✓ Long‐Term: Up to 12 months (e.g., alignment studies). ✓ Variable: Some studies followed patients until specific milestones (e.g., complete crowding resolution)	—	• 30 Hz (20 min/day): No significant acceleration in most studies (12 RCTs) • 113 Hz (10 min/day): Significant acceleration in CRR (1 RCT) • 120 Hz (5 min/day): Reduced aligner change intervals (1 RCT) • 125 Hz (15 min/day): Faster CRR (1 RCT) • Pain Reduction: Mixed results: Some studies reported reduced pain with vibration	NF	NC
	Keerthana et al. [23], India	RTM DR/CR/SC	✓ LII On plaster models by digital caliper or on digital models ✓ RCR: mm per month, Direct in the patient’s mouth or on plaster/ digital models ✓ SC: mm per month on study models by digital caliper	✓ Short‐Term Monitoring (Days to Weeks): Measured outcomes from 0 days (immediate) up to 3 months (e.g., 24 h, 48 h, weekly, or 4–12‐week intervals). ✓ Medium‐Term Monitoring (Weeks to Months): Tracked changes at 5–10‐week intervals or monthly evaluations until treatment completion ✓ Long‐Term Monitoring (Until Treatment Completion): Follow‐up until final outcomes	• (10 RCTs/310 PT): 30 Hz vibratory devices (e.g., AcceleDent, powered toothbrushes) accelerate orthodontic tooth movement (mean difference: 0.34 mm, 95% CI: 0.25–0.42, *p* < 0.00001)	—	NM	NC
	García Vega et al. [28], Mexico	RTM DR/CR/SC	✓ LII On plaster models by digital caliper or on digital models ✓ Tracking Percentage ✓ RCR: mm per month, Direct in the patient’s mouth or on plaster/digital models ✓ SC: mm per month on study models by digital caliper	Varied Durations: ✓ Ranged from 5 days to full treatment completion (e.g., space closure or alignment) ✓ Common intervals: 10 weeks, 3 months, 60 days, and 1‐month post‐vibration	—	• Limited Evidence: Most studies had high or moderate risk of bias, with only 4 out of 15 suggesting vibrations might accelerate tooth movement • No Clear Benefit: Current evidence does not conclusively show that vibrations increase tooth movement speed, reduce alignment time, or improve canine retraction • Method Issues: Variations in devices, vibration types, and short follow‐up periods weaken the reliability of results • Future Needs: High‐quality, standardized trials (especially for high‐frequency vibrations) with longer follow‐up are critical to confirm effectiveness	M Institutional resources of the Meritorious Autonomous University of Puebla funded this research	NC
	Dutta et al. [13], India	RTM DR/CR/SC	✓ LII On plaster models by digital caliper or on digital models ✓ Tracking Percentage ✓ RCR: mm per month, Direct in the patient’s mouth or on plaster/digital models ✓ SC: mm per month on plaster or digital models by digital caliper	✓ Most studies: 3–6 months (tooth movement and pain tracking) ✓ Initial alignment studies: 8–12 weeks ✓ CRR/ SC studies: 3–12 months (case‐dependent) ✓ Aligner studies: 3–9 months (with aligner changes every 7–14 days)	• LII (5 RCTs/209 PT): No significant difference between vibration and control groups (SMD = 0.16, CI: −0.11 to 0.43, *p* > 0.05) • RCR (6 RCTs/244 PT): Significant improvement in the vibration group (SMD = 2.48, CI: 0.90–4.07, *p* < 0.05), but high heterogeneity (*I* ^2^ = 96%, *p* < 0.00001) • Pain (VAS) (3 RCTs/144 PT): No significant reduction in pain (SMD = −0.31, CI: −0.68 to 0.06, *p* > 0.05)	—	NF	NC


Photobiomodulation	Almeida et al. [31], Brazil	RCR (U/L) (mm/month)	✓ RCR: mm per month, directly in the patient’s mouth by digital caliper	✓ Duration: 1–4.5 months (varied across studies) ✓ Frequency: Monthly measurements (tracked movement over 3–4.5 months) ✓ Focus: Short‐term effects only; long‐term outcomes not assessed	RCR (5 RCTs/63 PT): • Maxilla: Significant acceleration (0.33 mm difference, *p* = 0.012) at 3 months. • Mandible: Significant acceleration (1.03 mm difference, *p* < 0.0001) at 1 month • No overall acceleration: No consistent evidence across time points • No clear benefit of LLLT for accelerating orthodontic tooth movement	—	NF	NC
	Imani et al. [24], Iran	RCR (U/L) (mm/month)	✓ Undefined	✓ Duration: Measurements taken at 6 intervals: 21 days, 1 month, 1.5 months, 2 months, 3 months, and 4.5 months ✓ Frequency: varied per study ✓ Focus: Short‐term effects only; long‐term outcomes not assessed	• Tooth Movement Distance (mm): Significant increase in LLLT group vs. control at all intervals: ✓ 21 days (2 RCTs/33 PT): MD = 0.74 (95% CI: 0.17–1.31; *p* = 0.01) ✓ 1 month (4 RCTs/59 PT): MD = 0.40 (95% CI: 0.10–0.69; *p* = 0.008) ✓ 1.5 months (1 RCTs/11 PT): MD = 0.72 (95% CI: 0.51–0.93; *p* < 0.00001) ✓ 2 months (2 RCTs/44 PT): MD = 0.84 (95% CI: 0.23–1.44; *p* = 0.006) ✓ 3 months (3 RCTs/48 PT): MD = 0.92 (95% CI: 0.06–1.78; *p* = 0.04) ✓ 4.5 months (1 RCTs/20 PT): MD = 1.53 (95% CI: 0.92–2.14; *p* < 0.00001) • LLLT significantly speeds up canine movement and reduces treatment time	—	NM	NC
	Deana et al. [32], Chile	OTM acceleration, monthly/accumulated rates, and ED efficacy	✓ LII On plaster models by digital caliper or on digital models ✓ RCR: mm per month, Direct in the patient’s mouth or on plaster models by Digital caliper Reference wires and palatal plugs 3D models/visual assessments ✓ SC: mm per month on study models	✓ Duration: CR: 8 weeks–10 months Alignment: not explicitly specified ✓ Frequency: Weekly, biweekly, or monthly ✓ Focus: Short‐term OTM acceleration	RCR: • First month OTM (3 RCTs/38 PT): ED 50–75 J/cm²: MD 0.58 mm (95% CI: 0.36–0.80; *p* < 0.00001) • Second month OTM (2 RCTs/23 PT): ED 50–75 J/cm²: MD 0.41 mm (95% CI: 0.14–0.68; *p* = 0.003). • Accumulated OTM (4 RCTs/59 PT): ED 50–75 J/cm²: MD 1.40 mm (95% CI: 0.94–1.85; *p* < 0.00001) • No significant acceleration with ED 33–42 J/cm² or 200–214 J/cm² • PBMT with 50–75 J/cm² per tooth significantly accelerates OTM in the short term	—	NF	NC
	Bakdach et al. [33], Syria	RTM CR Alignment time SC velocity	✓ LII On plaster models by digital caliper or on digital models ✓ RCR: mm per month, Direct in the patient’s mouth or on plaster models by Digital caliper Reference wires and palatal plugs 3D models/visual assessments ✓ SC: mm per month on study models	✓ Duration: Ranged from 8 weeks to 6 months ✓ Frequency: Weekly, biweekly, or monthly ✓ Focus: Short‐term acceleration	Upper CR: • Month 1 (9 RCTs/113 PT): No significant difference (WMD = 0.21 mm, 95% CI [−0.09, 0.51], *p* = 0.16) • Month 2 (12 RCTs/168 PT): Significant acceleration (WMD = 0.50 mm, 95% CI [0.29, 0.72], *p* < 0.001) • Month 3 (6 RCTs/89 PT): Significant acceleration (WMD = 0.49 mm, 95% CI [0.02, 0.96], *p* = 0.04) Lower CR: • Month 1 (3 RCTs/34 PT): No significant difference (WMD = 0.56 mm, 95% CI [−0.37, 1.50], *p* = 0.24) • Month 2 (5 RCTs/74 PT): Significant acceleration (WMD = 0.28 mm, 95% CI [0.17, 0.40], *p* < 0.001) • Month 3 (3 RCTs/42 PT): Significant acceleration (WMD = 0.52 mm, 95% CI [0.40, 0.63], *p* < 0.001) – LLLT accelerates tooth movement, but clinical significance is questionable (low‐quality evidence) – No consensus on optimal laser dosage (Recommendation: report total joules/month instead of J/cm²)	—	NF	NC
	Camacho et al. [25], Colombia	RTM CR PR Alignment time	✓ LII On plaster models by digital caliper or on digital models ✓ RCR: mm per month, Direct in the patient’s mouth or on plaster/digital models/Stereomicroscope	✓ Duration: Ranged from 45 days to 3–4 months. ✓ Frequency: varied between 2 and 9 sessions, typically administered weekly or biweekly ✓ Focus: Short‐term acceleration	—	• 780–830 nm: Most studies showed significant tooth movement acceleration within this range • 24% increase in tooth movement speed compared to control groups (calculated across 9 studies) • 780–809 nm: Highest acceleration rates (up to 34–54% in specific studies) • 830–860 nm: Moderate acceleration (e.g., 26% reduction in treatment time) • > 900 nm: No significant effect (e.g., 904 nm)	NF	NC
	Grajales et al. [21], Spain	RTM CR ER	✓ RCR: mm per month, Direct in the patient’s mouth or on plaster/ digital models ✓ SC: mm per month on study models by digital caliper	✓ Duration: 3–4 months ✓ Frequency: Varied: Weekly, biweekly, or monthly ✓ Focus: Short‐term acceleration	Upper CR: ✓ Significant acceleration in LLLT groups during months 1–3: – Month 1 (12 RCTs/143 PT): OR = 0.28 (95% CI: 0.07–0.48) – Month 2 (13 RCTs/181 PT): OR = 0.52 (95% CI: 0.31–0.73) – Month 3 (10 RCTs/143 PT): OR = 0.41 (95% CI: 0.03–0.79) – Month 4 (3 RCTs/51 PT): No significant difference (OR = 0.41, 95% CI: −0.11 to 0.94) • Wavelength ≤810 nm and energy density ≤5.3 J/cm² showed statistically significant acceleration (subgroup analysis)	—	NF	NC
	Jnaneshwar et al. [34], India	RTM CR Alignment time	✓ LII On plaster models by digital caliper or on digital models ✓ RCR: mm per month, Direct in the patient’s mouth or on plaster models by Digital caliper	✓ Duration: Not explicitly mentioned in most studies ✓ Frequency: Varied: Weekly, biweekly, or monthly ✓ Limitations: No long‐term follow‐up ✓ Focus: Short‐term acceleration	• RCR: – (4 RCTs/ 48 PT): LLLT applied ≥4 times in the first month significantly increased OTM (SMD = 0.46 mm, *p* = 0.03), with no heterogeneity (*I*² = 0%) • Reduced Alignment Duration: – (2 RCTs/60 PT): LLLT reduced anterior alignment time by 25.58 days (*p* < 0.00001), with no heterogeneity (*I*² = 0%)	—	NF	NC
	Malik et al. [14], India	RTM CR Alignment time	✓ LII On plaster models by digital caliper or on digital models ✓ RCR: mm per month, Direct in the patient’s mouth or on plaster models by Digital caliper	✓ Duration: Varied: 67 days–6 months ✓ Frequency: Varied: Weekly, biweekly, or monthly ✓ Limitations: ✓ Focus: Limitations: No long‐term follow‐up ✓ Focus: Short‐term acceleration	(OTM) Rate (6 RCTs): • Significant acceleration observed in OTM starting from the 2nd month (WMD = 0.50 mm, *p* < 0.001) and 3rd month (WMD = 0.49 mm, *p* = 0.04) • No significant difference in the 1st month (WMD = 0.20 mm, 95% CI: −0.09 to 0.51, *p* = 0.26)	—	NF	NC
	Hmida et al. [35], Tunisia	RTM CR Alignment time	✓ LII On plaster models by digital caliper or on digital models ✓ RCR: mm per month, Direct in the patient’s mouth or on plaster models by Digital caliper	✓ Duration: Short‐Term (1–3 months) Medium‐Term (3–7 months) Variable/Unspecified ✓ Frequency: Weekly, biweekly, monthly, or mixed intervals	—	• Efficacy: 12 out of 14 studies demonstrated that PBM accelerates orthodontic tooth movement by 12.5%–40% and reduces treatment duration by 22%–26% • Heterogeneity: Varied treatment parameters (e.g., 810–980 nm wavelength, sessions 3–16)	NM	NM

*Note:* SRs were organized using the physical acceleration method (General Physical Adjunctive Intervention, BES, vibration, PBM) and sorted chronologically (oldest to newest) within each category.

Abbreviations: BES, bioelectric stimulation; CR, Canine Retraction; DR, decrowding; ER, En‐mass retraction; Hz, Hertz; LED, light‐emitting diode; LII, Little’s Irregularity Index; LLLT, low level laser therapy; LVF, light vibrational forces; M, mentioned; MD, mean deviation; Min, minute; mm, millimeter; NC, no conflict; NF, no funding; NM, not mentioned; OTM, orthodontic tooth movement; PBM, photobiomodulation; PCPDI, proximal contact point discrepancy index; PR, premolar retraction; PS, primary study; PT, patient; RCR, rate of canine retraction; RCT, randomized clinical trials; RTM, rate of tooth movement; SC, space closure; SMD, standard mean deviation; SR, systematic review; VD, vibration device; WMD, weighted mean difference.

For LLLT, studies using wavelengths between 780 and 830 nm demonstrated up to a 54% increase in the rate of tooth movement [25], with significantly greater effects in the mandible (MD = 1.03 mm, *p*  < 0.0001) compared to the maxilla (MD = 0.33 mm, *p* = 0.012) [31]. LLLT also shortened treatment duration by ~25 days (*p*  < 0.00001) [29], with the greatest acceleration observed during the first 3 months (weighted mean difference [WMD] = 0.49–0.50 mm, *p*  < 0.04) [14, 33], followed by a statistically reduced effect in the fourth month [21]. Twelve out of 14 studies confirmed a 12.5%–40% increase in movement speed [35], supporting LLLT as a promising technique, provided that photonic and temporal parameters are precisely controlled (Table 2).

LED therapy improved alignment by an average of 24.5 days (*p* = 0.008), though it did not significantly affect space closure or the average daily rate of tooth movement (*p* = 0.07) [27] (Table 2).

BES demonstrated significant acceleration in alleviating crowding by the third month (SMD = 0.69, *p*  < 0.05), but no statistically significant effect was observed by the fifth month (SMD = 1.64, *p*  > 0.05) [16]. Descriptive data from selected studies indicated a 30%–40% increase in movement speed [16].

Table 1 summarizes the findings of the included systematic reviews.

### 3.3. Characteristics of Primary Studies Included in SRs

Seventeen SRs incorporated 76 primary studies published between 2004 and 2024, with detailed characteristics in Supporting Informations 4–6: Tables S4–S6. The overall CCA was 0.11 (11% overlap), indicating study repetition inflated conclusions (Supporting Information 7: Table S7). For vibration devices, 7 SRs analyzed 28 RCTs [36–63], focusing on leveling/alignment (13 RCTs), canine retraction (11 RCTs), and En‐mass retraction (4 RCTs) (Supporting Information 4: Table S4), showing considerable overlap (CCA = 0.36, 36%; Supporting Information 8: Table S8). For PBM, 10 SRs assessed 44 RCTs [64–107], covering leveling/alignment (9 RCTs), canine retraction (28 RCTs), and En‐mass retraction (4 RCTs) (Supporting Information 5: Table S5), with notable overlap (CCA = 0.19, 19%; Supporting Information 9: Table S9). 1 SR on BES included 4 RCTs [108–111], addressing leveling/alignment (1 RCT) and canine retraction (3 RCTs) (Supporting Information 6: Table S6).

### 3.4. Methodological Quality Assessment (AMSTAR‐2)

**Figure 2:**
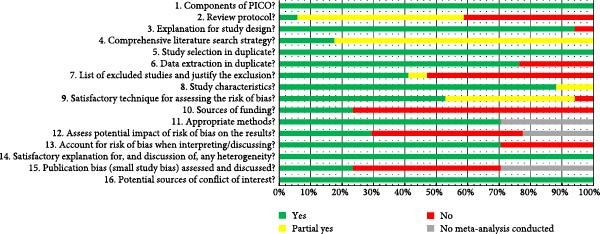
Methodological quality of the selected systematic reviews according to the 16 items proposed by the AMSTAR 2 tool.

All systematic reviews exhibited methodological shortcomings, with quality ranging from critically low [14, 21, 24, 27, 31, 32, 34, 35] to moderate [25, 28]. Results from the AMSTAR‐2 tool assessments are summarized in Table 3 and Figure 2, with detailed rationale in Supporting Information 10: Table S10.

**Table 3:** The AMSTAR‐2 (a measurement tool to assess systematic reviews‐2) criteria for the included systematic reviews.

Domains	General PAI	BES	VDs						PBM								
	El‐Angbawi et al. [4]	Dutta et al. [16]	Aljabaa et al. [26]	Abd Elmotaleb et al. [27]	Bakdach et al. [22]	Keerthana et al. [23]	García Vega et al. [28]	Dutta et al. [13]	Almeida et al. [31]	Imani et al. [24]	Deana et al. [32]	Bakdach et al. [33]	Camacho et al. [25]	Grajales et al. [21]	Jnaneshwar et al. [34]	Malik et al. [14]	Hmida et al. [35]
1. Did the research questions and inclusion criteria for the review include the components of PICO?	Yes	Yes	Yes	Yes	Yes	Yes	Yes	Yes	Yes	Yes	Yes	Yes	Yes	Yes	Yes	Yes	Yes
2. Did the report of the review contain an explicit statement that the review methods were established prior to the conduct of the review and did the report justify any significant deviations from the protocol?	Yes	Partial Yes	No	Partial Yes	No	Partial Yes	Partial Yes	Yes	Partial Yes	No	No	No	Partial Yes	Partial Yes	Yes	No	No
3. Did the review authors explain their selection of the study designs for inclusion in the review?	Yes	Yes	Yes	No	Yes	Yes	Yes	Yes	Yes	Yes	Yes	Yes	Yes	Yes	Yes	Yes	Yes
4. Did the review authors use a comprehensive literature search strategy?	Partial Yes	Partial Yes	Partial Yes	Partial Yes		Partial Yes	Partial Yes	Partial Yes	Partial Yes	Partial Yes	Partial Yes		Partial Yes	Partial Yes	Partial Yes	Partial Yes	Partial Yes
5. Did the review authors perform study selection in duplicate?	Yes	Yes	Yes	Yes	Yes	Yes	Yes	Yes	Yes	Yes	Yes	Yes	Yes	Yes	Yes	Yes	Yes
6. Did the review authors perform data extraction in duplicate?	Yes	Yes	Yes	Yes	Yes	Yes	Yes	Yes	Yes	No	Yes	Yes	No	Yes	Yes	No	No
7. Did the review authors provide a list of excluded studies and justify the exclusions?	Yes	No	Yes	No	Yes	Partial Yes	Yes	No	No	No	No	Yes	Yes	Yes	No	No	No
8. Did the review authors describe the included studies in adequate detail?	Yes	Yes	Yes	Partial Yes	Yes	Yes	Yes	Yes	Partial Yes	Yes	Yes	Yes	Yes	Yes	Yes	Yes	Yes
9. Did the review authors use a satisfactory technique for assessing the risk of bias (RoB) in individual studies that were included in the review?	Yes	Partial Yes	Yes	Partial Yes	Partial Yes	Partial Yes	Partial Yes	Yes	Partial Yes	Yes	Partial Yes	Yes	Yes	Yes	Yes	Yes	Yes
10. Did the review authors report on the sources of funding for the studies included in the review?	Yes	No	Yes	Yes	No	No	No	No	No	No	No	Yes	No	No	No	No	No
11. If meta‐analysis was performed did the review authors use appropriate methods for statistical combination of results?	Yes	Yes	No meta‐analysis conducted	Yes	No meta‐analysis conducted	Yes	No meta‐analysis conducted	Yes	Yes	Yes	Yes	Yes	No meta‐analysis conducted	Yes	Yes	Yes	No meta‐analysis conducted
12. If meta‐analysis was performed, did the review authors assess the potential impact of RoB in individual studies on the results of the meta‐analysis or other evidence synthesis?	No	No	No meta‐analysis conducted	No	No meta‐analysis conducted	No	No meta‐analysis conducted	No	No	No	No	Yes	No meta‐analysis conducted	No	No	No	No meta‐analysis conducted
13. Did the review authors account for RoB in individual studies when interpreting/ discussing the results of the review?	Yes	Yes	Yes	No	Yes	No	Yes	Yes	No	No	Yes	Yes	Yes	No	Yes	Yes	Yes
14. Did the review authors provide a satisfactory explanation for, and discussion of, any heterogeneity observed in the results of the review?	Yes	Yes	Yes	Yes	Yes	Yes	Yes	Yes	Yes	Yes	Yes	Yes	Yes	Yes	No	Yes	Yes
15. If they performed quantitative synthesis did the review authors carry out an adequate investigation of publication bias (small study bias) and discuss its likely impact on the results of the review?	No	Yes	No meta‐analysis conducted	No	No meta‐analysis conducted	Yes	No meta‐analysis conducted	Yes	No	No	No	Yes	No meta‐analysis conducted	No	No	No	No meta‐analysis conducted
16. Did the review authors report any potential sources of conflict of interest, including any funding they received for conducting the review?	Yes	Yes	Yes	Yes	Yes	Yes	Yes	Yes	Yes	Yes	Yes	Yes	Yes	Yes	Yes	Yes	Yes
Overall quality	LQ	LQ	LQ	CLQ	LQ	LQ	MQ	LQ	CLQ	CLQ	CLQ	LQ	MQ	CLQ	CLQ	CLQ	CLQ

Abbreviations: BES, bioelectric stimulation; CLQ, critical low quality; LQ, low quality; MQ, moderate quality; PAI, physical adjunctive interventions; PBM, photobiomodulation; VDs, vibration devices.

All reviews met requirements for domains 1, 5, 14, and 16. Among the seven critical domains, compliance was highest in domains 9, 11, and 13 but lowest in domains 2, 4, and 15. Key deficiencies included: 41.1% lacking prior protocol registration; 52.94% of registered protocols failing to explain adherence or deviations; 92% omitting comprehensive searches (e.g., manual/gray literature/unpublished data); and only 23.52% (four reviews [13, 16, 23, 33]) analyzing publication bias.

### 3.5. ROBIS

**Figure 3:**
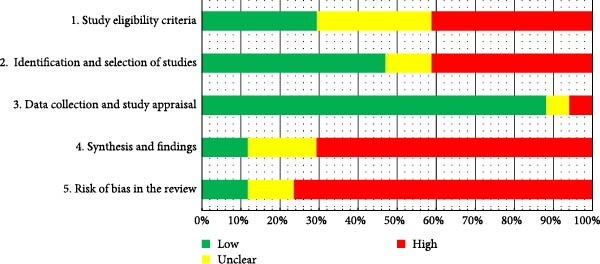
Methodological risk of bias assessment of the selected systematic reviews based on the four stages proposed by the ROBIS tool.

A ROBIS assessment of 17 SRs found 2 with low risk [4, 34], 2 unclear [13, 33], and 13 high risk [14, 16, 21–25, 27, 28, 31, 32, 35], summarized in Table 4 and Figure 3 with detailed rationale in Supporting Information 11: Table S11. Domain 1 faced issues from unregistered protocols and eligibility restrictions. Domain 2 was limited by insufficient database coverage and a lack of supplementary methods. Domain 3 demonstrated high compliance via reliable data collection, approved bias tools, and accurate reporting, while Domain 4 performed poorly by failing to assess heterogeneity and conducting meta‐analyses combining high‐bias/varied‐design studies without sensitivity analyses, undermining reliability and highlighting the need for improved rigor in heterogeneity handling and meta‐analyses.

**Table 4:** Risk of bias in systematic reviews summary.

Review	Phase 2				Phase 3
	1. Study eligibility criteria	2. Identification and selection of studies	3. Data collection and study appraisal	4. Synthesis and findings	Risk of bias in the review
El‐Angbawi et al. [4]	☺	☺	☺	☺	☺
Dutta et al. [16]	☺	☺	☺	☹	☹
Aljabaa et al. [26]	?	☺	☺	☹	☹
Abd Elmotaleb et al. [27]	☹	☹	☺	☹	☹
Bakdach et al. [22]	?	☺	☺	☹	☹
Keerthana et al. [23]	☹	☹	☺	?	☹
García Vega et al. [28]	☹	☹	☺	☹	☹
Dutta et al. [13]	☺	☺	☺	?	?
Almeida et al. [31]	☹	?	☹	☹	☹
Imani et al. [24]	?	?	☺	☹	☹
Deana et al. [32]	?	☹	?	☹	☹
Bakdach et al. [33]	?	☺	☺	?	?
Camacho et al.[25]	☹	☹	☺	☹	☹
Grajales et al. [21]	☺	☺	☺	☹	☹
Jnaneshwar et al. [34]	☺	☺	☺	☺	☺
Malik et al. [14]	☹	☹	☺	☹	☹
Hmida et al. [35]	☹	☹	☺	☹	☹

*Note:* ☺ = low risk; ☹ = high risk; ? = unclear risk.

### 3.6. Reassessment of ROB in Primary Studies Using ROB2

The risk of bias for all primary studies was re‐evaluated using the ROB2 tool due to concerns including low evidence quality (AMSTAR‐2) and high bias risk (ROBIS) in most systematic reviews, significant overlap (CCA) of primary studies across reviews, and the use of ROB1 in 12 systematic reviews (four pre‐ [24, 26, 27, 32] and eight post‐2019 [4, 14, 22, 23, 25, 28, 33, 34]). Variability in bias risk persisted for repeated studies across reviews, even with identical assessment tools, and some primary studies underwent evaluation with multiple tools. Reassessment of ROB in Primary Studies in systematic reviews is justified by Supporting Information 12: Table S12.

Original and updated bias risks are documented in Supporting Informations 4–6: Tables S4–S6 and Supporting Informations 14 and 15: Figures S1 and S2, with detailed assessment rationales in Supporting Information 13: Table S13.

### 3.7. Findings From the Pooled Primary Studies After Removal of Overlap

The meta‐analysis results (MA‐pool) are presented in Figures 4–12, and both MA‐pool and individual findings (IF‐pool) are summarized in Tables 5–7.

**Figure 4:**
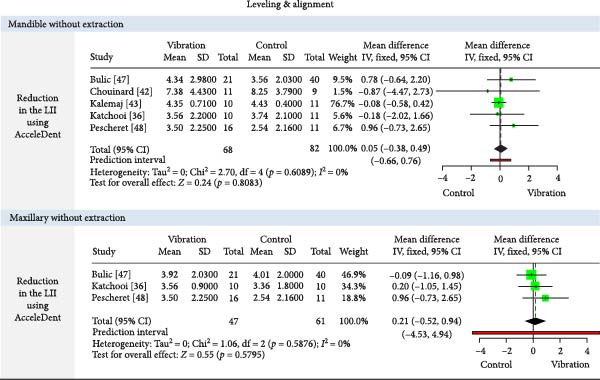
Forest plot comparing AcceleDent vibration therapy versus control for reduction in Little’s irregularity index (LII) during orthodontic leveling and alignment in mandibular and maxillary arches without extractions.

**Figure 5:**
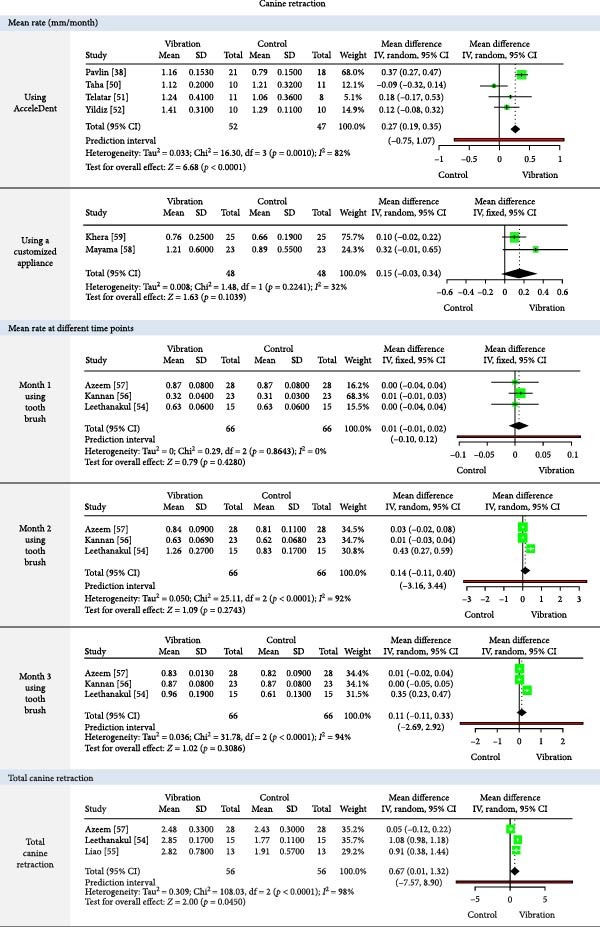
Forest plot comparing vibration and control for mean canine retraction rate (overall), monthly canine retraction (at months 1–3), and total canine retraction.

**Figure 6:**
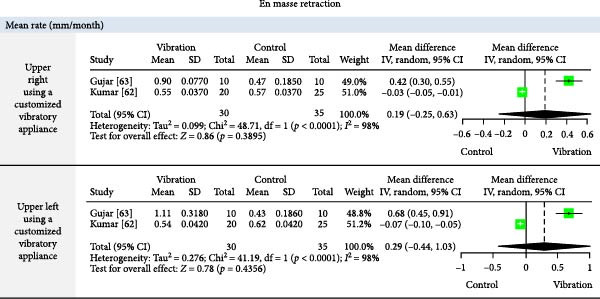
Forest plot comparing vibration vs. control for mean En‐masse retraction rate (mm/month) in upper quadrants.

**Figure 7:**
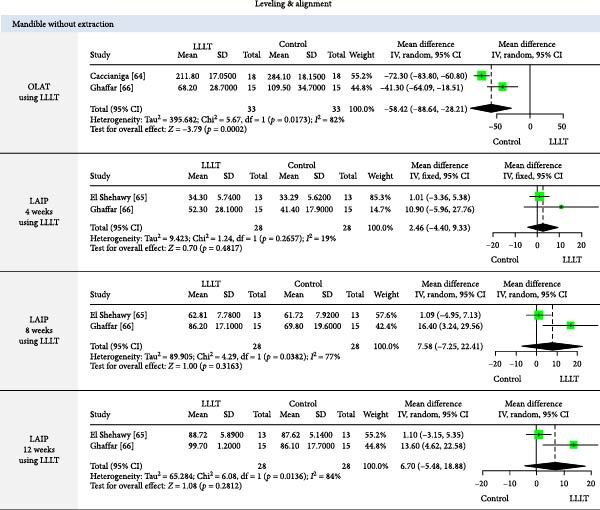
Forest plot of low‐level laser therapy (LLLT) versus control effect on orthodontic leveling and alignment time (OLAT) and leveling and alignment improvement percentage (LAIP) in mandible without extraction.

Figure 8Forest plot comparing low‐level laser therapy (LLLT) and control for monthly upper canine retraction (mm) (at months 2–3), cumulative upper canine retraction (mm from baseline) (at 21 days and months 1–3), and total upper canine retraction (mm) (post‐treatment).

**Figure:**
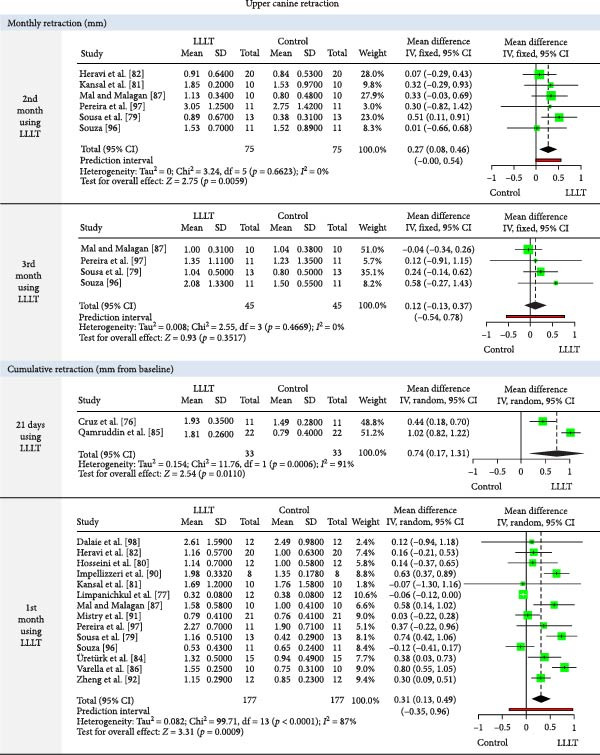

**Figure:**
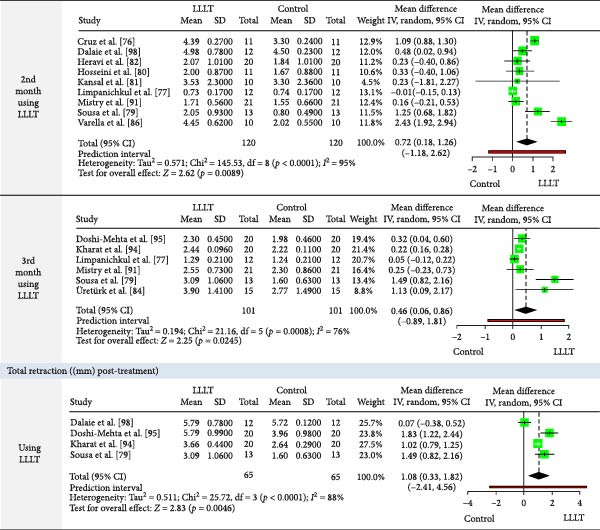

**Figure 9:**
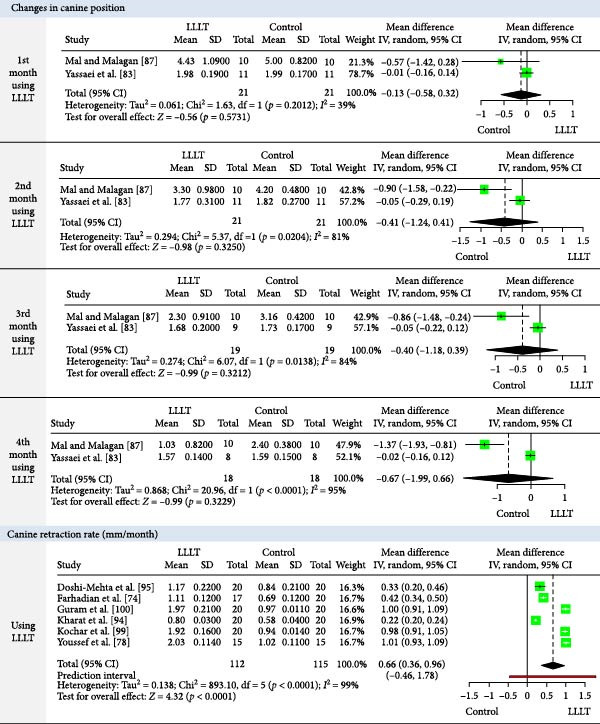
Forest plot comparing low‐level laser therapy (LLLT) versus control for monthly changes in upper canine position (at months 1–4) and upper canine retraction rate (mm/month).

**Figure 10:**
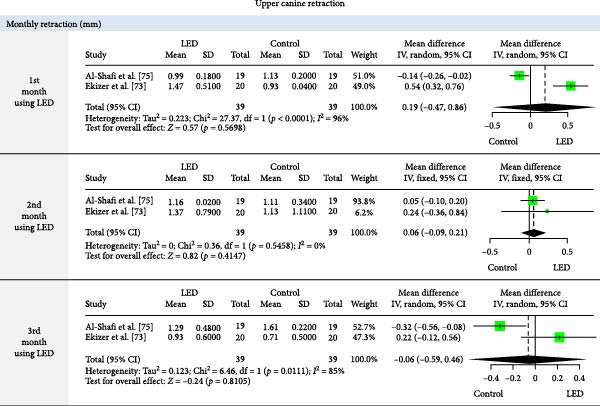
Forest plot comparing light‐emitting diode (LED) therapy versus control for monthly upper canine retraction rate (mm) (at months 1–3).

**Figure 11:**
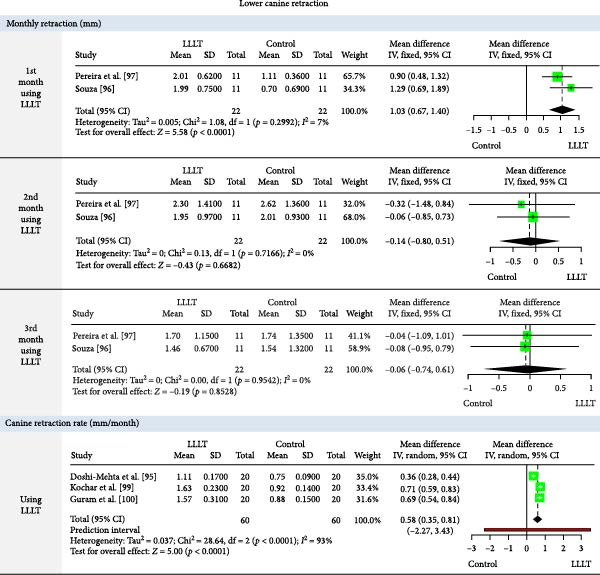
Forest plot of low‐level laser therapy (LLLT) versus control for monthly lower canine retraction rate (mm) (at months 1–3) and overall lower canine retraction rate (mm).

**Figure 12:**
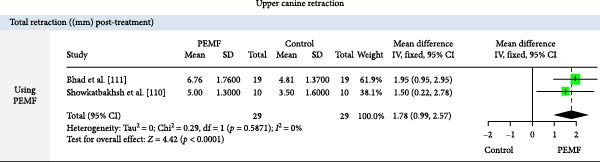
Forest plot of pulsed electromagnetic fields (PEMF) versus control for total upper canine retraction (mm).

**Table 5:** Findings from the pooled primary studies on accelerating orthodontic treatment using VDs.

Intervention			VDs	Treatment effect	95% CI	No. of primary trials	No. of events	*p*‐Value
Leveling & alignment	Mandible without extraction	Reduction in the LII (mm)	AcceleDent	0.05	−0.38, −0.49	5 RCTs [37, 38, 44, 45, 47]	150	0.808^a^
			Tooth Masseuse	0.60	—	1 RCT [40]	64	>0.05
			Customized appliance	0.83	—	1 RCT [41]	12	=0.05
	Mandible with extraction		AcceleDent	0.01	−0.02, 0.03	1 RCT [43]	53	0.66
	Maxillary without extraction		AcceleDent	0.21	−0.52, 0.94	3 RCTs [44, 45, 47]	109	0.579^a^

Canine retraction	Mean CRR (mm/month)		AcceleDent	0.27	0.19, 0.35	4 RCTs (49‐52)	99	<0.0001^a^
			Customized appliance	0.15	‐0.03, 0.34	2 RCTs (58, 59)	96	0.103^a^
	Mean CR at different time points (mm)	1st month	Tooth Bruch	0.01	−0.01, 0.02	3 RCTs [54, 56, 57]	132	0.428^a^
		2nd month		0.14	−0.11, 0.40	3 RCTs [54, 56, 57]	132	0.274^a^
		3rd month		0.11	−0.11, 0.33	3 RCTs [54, 56, 57]	132	0.308^a^
	Total CR (mm)			0.67	0.01, 1.32	3 RCTs (54, 56, 57)	112	0.045^a^

En masse retraction	Mean ERR (mm/month)	Total upper	AcceleDent	0.05	−0.23, 0.33	1 RCT [60]	40	0.73
		Upper right	Customized appliance	0.19	−0.25, 0.63	2 RCTs [62, 63]	65	0.389^a^
		Upper left		0.29	−0.44, 1.03	2 RCTs [62, 63]	65	0.435^a^
		Lower right		−0.020	−0.103, 0.063	1 RCT [62]	45	1
		Lower left		‐0.020	−0.108, 0.069	1 RCT [62]	45	1

Abbreviations: CI, confidence interval; CR, Canine retraction; CRR, Canine retraction rate; ER, En‐mass retraction; ERR, En‐mass retraction rate; LII, Little’s Irregularity Index; RCT, randomized clinical trials; VDs, vibration devices.

^a^Meta‐analysis of studies with pooled data (MA‐Pool).

**Table 6:** Findings from the pooled primary studies on accelerating orthodontic treatment using PBM.

Intervention				PBM	Treatment effect	95% CI	No. of primary trials	No. of events	*p*‐Value
Leveling and Alignment	Mandible without extraction	OLAT (day)		LLLT	−58.42	−88.64, −28.21	2 RCTs [64, 66]	66	0.0002^a^
		LAIP (%)	Early alignment 4 weeks		2.46	−5.96, 27.76	2 RCTs [65, 66]	56	0.481^a^
			Early alignment 8 weeks		7.58	−7.25, 22.41	2 RCTs [65, 66]	56	0.316^a^
			Early alignment 12 weeks		6.70	−5.48, 18.88	2 RCTs [65, 66]	56	0.281^a^
		OLAT (day)		LED	−19.5	−37.45, −1.55	1 RCT [67]	34	0.043
				MD = −57	—	1 RCT (68)	89	<0.001	
	Maxilla with extraction	OLAT (day)		LLLT	−28.00	−39.34, −16.66	1 RCT [69]	26	<0.001
	Maxilla without extraction	OLAT (day)		LED	−22.3	—	1 RCT [70]	26	0.028
	Both jaws without extraction	Alignment rate (mm/week)		LED	0.63	—	1 RCT [71]	90	<0.00001
		Effect of LLLT on daily wear time (hours)		LLLT	LLLT enhanced success Reducing aligner wear time from 22 to 12 hours/day.	—	1 RCT [72]	21	<0.001

Upper Canine Retraction	Mean CRR (mm/day)			LED	0.005	−0.001, 0.012	1 RCT [74]	39	0.15
	Monthly CR (mm)	1st month			0.19	−0.47, 0.86	2 RCTs [73, 75]	78	0.569^a^
		2nd month			0.06	−0.09, 0.21	2 RCTs [73, 75]	78	0.414^a^
		3rd month			−0.06	−0.59, 0.46	2 RCTs [73, 75]	78	0.810^a^
	Monthly CR (mm)	2nd month		LLLT	0.27	0.08, 0.46	6 RCTs [79, 81, 82, 87, 96, 97]	150	0.005^a^
		3rd month			0.12	−0.13, 0.37	4 RCTs [79, 87, 96, 97]	90	0.351^a^
		4th month			0.52	0.14, 0.90	1 RCT [87]	20	0.03
	Cumulative CR (mm from Baseline)	21 days			0.74	0.17, 1.31	2 RCTs [75, 85]	66	0.011^a^
		1st month			0.31	0.13, 0.49	14 RCTs [77, 79–82, 84, 86, 87, 90–92, 96–98]	354	0.0009^a^
		2nd month			0.72	0.18, 1.26	9 RCTs [76, 77, 79–82, 86, 91, 98]	240	0.0089^a^
		3rd month			0.46	0.06, 0.86	6 RCTs [77, 79, 84, 91, 94, 95]	202	0.02^a^
	Total CR ((mm) post‐treatment)				1.08	0.33, 1.82	4 RCTs [79, 94, 95, 98]	130	0.0046^a^
	Changes in canine position (mm)	1st month			−0.13	−0.58, 0.32	2 RCTs [83, 87]	42	0.576^a^
		2nd month			−0.41	−1.24, 0.41	2 RCTs [83, 87]	42	0.325^a^
		3rd month			−0.40	−1.18, 0.39	2 RCTs [83, 87]	38	0.321^a^
		4th month			−0.67	−1.99, 0.66	2 RCTs [83, 87]	36	0.322^a^
	CRR (mm/month)				0.66	0.36, 0.96	6 RCTs [74, 78, 94, 95, 99, 100]	227	<0.0001^a^
	Days to achieve space closure				13.14	—	1 RCT [88]	82	<0.001
	Total CR without extraction (mm)				0.63	—	1 RCT [93]	54	0.009

Lower Canine Retraction	Monthly CR (mm)	1st month		LLLT	1.03	0.67, 1.40	2 RCTs [96, 97]	44	<0.0001^a^
		2nd month			−0.14	−0.80, 0.51	2 RCTs [96, 97]	44	0.668^a^
		3rd month			−0.06	−0.74, 0.61	2 RCTs [96, 97]	44	0.852^a^
	CRR (mm/month)				0.58	0.35, 0.81	3 RCTs [95, 99, 100]	120	<0.0001^a^

En Masse Retraction	Daily ER (mm/day)			LLLT	0.04	—	1 RCT [101]	40	<0.0001
	Monthly ERR (mm/month)	Total upper			0.194	—	1 RCT [102]	24	0.017
		Upper right			0.18	0.05, 0.3	1 RCT [103]	45	0.003
		Upper left			0.19	0.06, 0.32	1 RCT [103]	45	0.002
		Lower right			0.16	0.12, 0.19	1 RCT [103]	45	<0.0001
		Lower left			0.185	0.13, 0.23	1 RCT [103]	45	<0.0001
	Monthly ERR (mm/month)			LED	0.22	0.09, 0.49	1 RCT [104]	45	0.007

Upper molars intrusion	Average at 6 months in adults			LLLT	−0.64	—	1 RCT [106]	20	> 0.05
	Average at 7 months in children				0.39	—	1 RCT [107]	28	0.018

Abbreviations: CI, confidence interval; CR, Canine retraction; CRR, Canine retraction rate; ER, En‐mass retraction; ERR, En‐mass retraction rate; LAIP, leveling and alignment improvement percentage; LED, light‐emitting diode; LLLT, low level laser therapy; OLAT, orthodontic leveling and alignment time; PBM, photobiomodulation; RCT, randomized clinical trials.

^a^Meta‐analysis of studies with pooled data (MA‐Pool).

**Table 7:** Findings from the pooled primary studies on accelerating orthodontic treatment using BES.

Intervention			BES device	Treatment effect	95% CI	No. of primary trials	No. of events	*p*‐Value
Leveling and alignment	Mandible without extraction	3 months of crowding reduction (mm)	BM	1.9	—	1 RCTs [108]	28	<0.05
Upper canine retraction	Cumulative CR (mm from baseline)	1st month	LIDEC	0.53	—	1 RCT [109]	14	0.001
	Total CR ((mm) post‐treatment)		PEMF	1.78	0.99, 2.57	2 RCTs [110, 111]	58	<0.0001^a^

Abbreviations: BES, bioelectric stimulation; BM, bimaxillary mouthpiece; CI, confidence interval; CR, Canine retraction; LIDEC, low‐intensity direct current; PEMF, pulsed electromagnetic field; RCT, randomized clinical trials; VDs, vibration devices.

^a^Meta‐analysis of studies with pooled data (MA‐Pool).

#### 3.7.1. Vibration Devices (Table 5, Figures 4–6)

Leveling and alignment: AcceleDent showed minimal or no clinical benefit in both mandibular and maxillary leveling (effect sizes: −0.12 to 0.21, *p*  > 0.05) across multiple RCTs [36, 39, 41–43, 47, 48]. Tooth Masseuse showed moderate improvement in one small trial [44], while a customized device showed better results (0.83, 1 RCT, *p* = 0.05) but in a very small sample (*n* = 12) [45].

Canine retraction: AcceleDent significantly accelerated retraction (0.27 mm/month, four RCTs, *p*  < 0.0001) [38, 50–52], while customized appliances [58, 59] and toothbrush [54, 56, 57] use showed inconsistent or nonsignificant effects. Total retraction was modest (0.67 mm, three RCTs, *p* = 0.045) [54, 55, 57].

En masse retraction: AcceleDent [60] and customized vibratory devices [62, 63] had no significant impact. Effects were inconsistent and generally negligible, especially in lower arches [62].

#### 3.7.2. PBM (Table 6, Figures 7–11)

Leveling and alignment (Figure 7): LLLT significantly reduced mandibular alignment time without extraction (−58.42 days, 2 RCTs, *p* = 0.0002) [64, 66], though it did not improve alignment percentage [65, 66]. LED therapy also shortened alignment time (−19.5 to −57 days; *p*  < 0.05) [67, 68] and increased alignment rate (0.63 mm/week; *p*  < 0.00001) [71]. LLLT reduced daily aligner wear from 22 to 12 h (*p*  < 0.001) [72], enhancing treatment efficiency.

Upper canine retraction (Figures 8–10): LLLT showed significant retraction in the 2nd (0.27 mm, six RCTs, *p* = 0.005) [79, 81, 82, 87, 96, 97] and 4th month (0.52 mm, 1 RCT, *p* = 0.03) [87]. Cumulative gains were significant up to 3 months (total: 1.08 mm, four RCTs, *p* = 0.0046) [79, 94, 95, 98]. Retraction rate reached 0.66 mm/month (6 RCTs, *p*  < 0.0001) [74, 78, 94, 95, 99, 100], with space closure accelerated by 13.14 days [88]. LED devices showed no significant effects (*p*  > 0.05).

Lower canine retraction (Figure 11): Significant retraction in the 1st month (1.03 mm, two RCTs, *p*  < 0.0001) [96, 97], with an overall rate of 0.58 mm/month, three RCTs [95, 99, 100]. Later months showed no notable improvement.

En masse retraction: LLLT showed daily retraction of 0.04 mm (one RCT, *p*  < 0.0001) [101]; LED showed monthly effects (0.22 mm, one RCT, *p* = 0.007) [104]. Total upper arch retraction was 0.194 mm/month (one RCT, *p* = 0.017) [102], with significant site‐specific effects in both upper and lower quadrants (*p*  < 0.01) [103].

Upper molar intrusion: In adults, LLLT showed a nonsignificant effect (−0.64, one RCT, *p*  > 0.05) [106]. In children, it was effective (0.39, one RCT, *p* = 0.018) [107].

#### 3.7.3. BES (Table 7, Figure 12)

BES devices significantly improved orthodontic outcomes. The bimaxillary mouthpiece device (BM) reduced mandibular crowding by 1.9 mm more than controls in 3 months (one RCT, *p*  < 0.05). Low‐intensity direct current (LIDEC) achieved 0.53 mm greater upper canine retraction in 1 month (one RCT, *p* = 0.001). PEMF showed the strongest effect, with 1.78 mm total retraction compared to control (two RCTs, *p*  < 0.0001) (Figure 12).

### 3.8. Overall Quality of the Evidence Across Studies

The assessment of evidence certainty based on the GRADE methodology is detailed in Tables 8–10, organized according to the acceleration modality (vibration, PBM, or BES). In general, the majority of the included studies were judged to provide low to very low certainty evidence, primarily due to concerns related to high risk of bias or some concerns in key ROB2 domains, coupled with imprecision arising from small sample sizes and wide confidence intervals (CIs).

**Table 8:** Summary of findings table according to the GRADE guidelines for the included trials on accelerating orthodontic treatment using VDs.

Interventions		Quality assessment criteria						Summary of findings		
		No. of studies	Risk of bias	Inconsistency	Indirectness	Imprecision	Other considerations	No. of patients	Effect	Overall quality of evidence
Accelerating leveling and alignment										
Lower Jaw without extraction			Serious	Not serious	Not serious	Serious	None	150	Adding AcceleDent did not produce a clinically important speed‐up in lower‐arch alignment	⨁⨁◯◯^a^ Low
Using AcceleDent		5 RCTs								
Using Tooth Masseuse		1 RCT	Serious	Not serious	Not serious	Serious	None	64	Adding the Tooth Masseuse vibrating appliance did not produce a clinically important increase in the rate of lower‐front‐teeth alignment	⨁⨁◯◯^b^ Low
Using a Customized appliance		1 RCT	Serious	Not serious	Not serious	Serious	None	12	Adding the customized vibration appliance significantly increased the rate of lower‐front‐teeth alignment and reduced pain during initial leveling and alignment	⨁⨁◯◯^c^ Low
Lower Jaw with extraction			Not serious	Not serious	Not serious	Serious	None	53	Adding AcceleDent did not produce a clinically important speed‐up in lower‐arch alignment	⊕⊕⊕◯^d^ Moderate
Using AcceleDent		1 RCT								
Upper jaw without extraction			Serious	Not serious	Not serious	Serious	None	56	Adding AcceleDent did not produce a clinically important speed‐up in upper‐arch alignment	⨁⨁◯◯^e^ Low
Using AcceleDent		3 RCTs								
Accelerating canine retraction										
Using AcceleDent	Mean rate (mm/month)	4 RCTs	Serious	Serious	Not serious	Very serious	None	107	≈ 0.3 mm/month faster (MD = +0.27 mm/month; 95 % CI +0.19 to +0.35)	⨁◯◯◯^f^ Very low
Using a customized appliance		2 RCTs	Serious	Serious	Not serious	Serious	None	48	Essentially, no clinically or statistically significant acceleration	⨁◯◯◯^g^ Very low
Using tooth brush	Mean rate at different time points	3 RCTs	Serious	Serious	Not serious	Serious	None	66	Essentially, no clinically or statistically significant acceleration	⨁◯◯◯^h^ Very low
	Total canine retraction	3 RCTs	Serious	Very serious	Not serious	Serious	None	56	+0.67 mm (0.01 to 1.32 mm) more retraction—favors vibration	⨁◯◯◯^i^ Very low
Accelerating en‐masse retraction										
Using AcceleDent	Mean rate (mm/month)	1 RCT	Serious	Not serious	Not serious	Serious	None	40	Essentially, no clinically or statistically significant acceleration	⨁⨁◯◯^j^ Low
Using a customized appliance		2 RCT	Serious	Very serious	Not serious	Serious	None	65	MD = 0.19 mm/month (95% CI −0.25 to 0.63)—an average gain of about two‐tenths of a millimeter per month, but the CI includes both slower and faster movement, so the difference is not statistically or clinically meaningful	⨁◯◯◯^k^ Very low

*Note:* High‐quality, further research is very unlikely to change our confidence in the estimate of effect. Low quality, Further research is very likely to have an important impact on our confidence in the estimate of effect and is likely to change the estimate. Moderate quality, further research is likely to have an important impact on our confidence in the estimate of effect and may change the estimate. Very low quality, We are very uncertain about the estimate.

Abbreviations: CI, confidence interval; GRADE, Grading of Recommendations; Assessment; Development; and Evaluations; LAIP, leveling and alignment improvement percentage; LED, light‐emitting diode; LLLT, low‐level laser therapy; mm, millimeter; OLAT, orthodontic leveling and alignment time; PBM, photobiomodulation; RCTs, randomized clinical trials.

^a^Declineone level for risk of bias (some concern in the randomization process [43, 48], deviations from intended interventions [43, 47, 48], missing outcome data [42, 48], and measurement of the outcome [47, 48], and high risk in the selection ofthe reported result [48]) and one level for imprecision.^3^

^b^Decline one level for risk of bias (some concern in the randomization process [44], and the selection of the reported result [44]) and one level forimprecision.^3^

^c^Decline one level for risk of bias (some concern due to issues in therandomization process [45], measurement of the outcome [45], and selection ofthe reported result [45]), and one level for imprecision.^3^

^d^Decline one level for imprecision.^3^

^e^Decline one level for risk of bias (some concern in the randomization process [47], deviations from intended interventions [47, 48], missing outcome data [48], and measurement of the outcome [47, 48], with high risk in the selection of the reported result [48]), and one level for imprecision.^3^

^f^Decline one level for risk of bias (some concern in the randomization process [50–52], deviations from intended interventions [50–52], measurement of theoutcome [50–52], and selection of the reported result [38, 50–52]), one level forInconsistency^1^ and two levels for Imprecision.^3^

^g^Decline one level for risk of bias (some concern in the measurement of theoutcome [59]), one level for Inconsistency^1^, and one level for Imprecision.^3^

^h^Decline one level for risk of bias (some concern in the randomization process [56, 57], measurement of the outcome [56], and selection of the reported result [57]), one level for Inconsistency^1^, and one level for Imprecision.^3^

^i^Decline one level for risk of bias (some concern in the randomization process [57], measurement of the outcome [55], and selection of the reported result [55, 57]), two levels for Inconsistency^1^, and one level for Imprecision.^3^

^j^Decline one level for risk of bias (some concern in the selection of thereported result [60]), and one level for Imprecision.^3^

^k^Decline one level for risk of bias (some concern in the randomization process [63], deviations from intended interventions [62, 63], and measurement of theoutcome [63]), two levels for Inconsistency^1^, and one level for Imprecision.^3^

^1^Wide variance of point estimates across studies study.

^2^Indirectness: Short assessment duration (study time frame), and a differencebetween desired (Patient’ important) and measured outcomes.

^3^Imprecision: Limited number oftrials and sample size.

**Table 9:** Summary of findings table according to the GRADE guidelines for the included trials on accelerating orthodontic treatment using PBM.

Interventions			Quality assessment criteria						Summary of findings		
			No. of studies	Risk of bias	Inconsistency	Indirectness	Imprecision	Other considerations	No. of patients	Effect	Overall quality of evidence
Accelerating leveling and alignment											
Lower jaw without extraction											
Using LLLT	OLAT		2 RCTs	Serious	Serious	Not serious	Not serious	Large absolute benefit (≈ 2 months faster); no clear publication‐bias signals	66	LLLT shortened alignment time by 58 days (Mean Difference −58.4 days, 95 % CI −88.6 to −28.2) – statistically significant	⨁⨁◯◯^a^ Low
	LAIP		2 RCTs	Serious	Not serious	Not serious	Serious	None	56	LLLT may slightly improve leveling and alignment improvement percentage (LAIP) compared to control, but without a statistically significant effect in the pooled analysis (*p* > 0.05)	⨁⨁◯◯b Low
Using LED	OLAT		1 RCT	Very serious	Not serious	Not serious	Serious	None	34	Mean difference −19.5 days (≈ 22 % faster alignment; *p* = 0.043 → statistically significant)	⨁◯◯◯^c^ Very low
Upper jaw with extraction											
Using LLLT	OLAT		1 RCT	Serious	Not serious	Not serious	Serious	None	26	LLLT shortened OLAT by ≈ 28 days (MD = –28 days, 95% CI –39 to –17; *p* < 0.001 ⇒ statistically significant)	⨁⨁◯◯^d^ Low
Upper jaw without extraction											
Using LED	OLAT		1 RCT	Serious	Not serious	Not serious	Serious	None	26	≈ 22 days faster alignment with LLLT (41 d vs. 63 d; significant, *p* = 0.028)	⨁⨁◯◯^e^ Low
Both jaws without extraction											
Using LLLT	Effect of LLLT on daily wear time (hours)		1 RCT	Serious	Not serious	Not serious	Serious	Large effect size	21	LLLT enhanced success, reducing aligner wear time from 22 to 12 h/day	⨁◯◯◯^f^ Very low
Using LED	Alignment rate (mm/week)		1 RCT	Serious	Not serious	Not serious	Serious	Manufacturer funding may introduce publication bias	90	Mean difference ≈ +0.63 mm/week (1.12 vs. 0.49 mm/week). About 2.3× faster alignment; statistically significant (*p* < 0.00001)	⨁◯◯◯^g^ Very low
Accelerating upper canine retraction											
Using LED	Mean rate (mm/day)		1 RCT	Serious	Not serious	Very serious	Very serious	None	39	+0.006 mm day⁻¹ (≈ +0.18 mm month⁻¹) 95 % CI ‐0.001 → +0.012 mm day⁻¹, NS (*p* = 0.15)	⨁◯◯◯^h^ Very low
	Monthly retraction (mm)		2 RCT	Serious	Serious	Not serious	Serious	None	39	LED may have little to no effect on monthly retraction rate; point estimates fluctuate (e.g. +0.19 mm [−0.47, 0.86] at 1st month; +0.06 mm [−0.09, 0.21] at 2nd month; −0.06 mm [−0.59, 0.46] at 3rd month), no statistically significant differences (*p* > 0.05)	⨁⨁◯◯^i^ Low
Using LLLT	Monthly retraction (mm)	2nd month	6 RCTs	Serious	Not serious	Not serious	Not serious	None	75	At 2nd month: LLLT increased monthly canine retraction by 0.27 mm (95% CI: 0.08 to 0.46), *p* = 0.0059, statistically significant.	⨁⨁⨁◯^j^ Moderate
		3rd month	4 RCTs	Serious	Serious	Not serious	Serious	None	45	At 3rd month: increase of 0.12 mm (95% CI: −0.13 to 0.37), *p* = 0.35, not statistically significant.	⨁⨁◯◯^k^ Low
	Cumulative retraction (mm from baseline)	21 days	2 RCTs	Serious	Serious	Not serious	Serious	None	33	Use of LLLT may accelerate cumulative upper canine retraction by approximately 0.74 mm more than control over 21 days	⨁⨁◯◯^l^ Low
		1st month	14 RCTs	Serious	Serious	Not serious	Serious	None	177	LLLT likely increased monthly canine retraction by ~0.3 mm (95% CI: 0.13 to 0.49), statistically significant but with substantial variability across studies.	⨁⨁◯◯^m^ Low
		2nd month	9 RCTs	Serious	Serious	Not serious	Not serious	None	120	LLLT likely increased monthly canine retraction by 0.72 mm (95% CI: 0.18 to 1.26)	⨁⨁◯◯^n^ Low
		3rd month	6 RCTs	Serious	Serious	Not serious	Serious	None	101	LLLT increased canine retraction by ~0.46 mm (95% CI 0.06 to 0.86) at 3 months vs. control (statistically significant, but wide variation)	⨁⨁◯◯^o^ Low
	Total retraction ((mm) post‐treatment)		4 RCTs	Serious	Serious	Not serious	Very Serious	None	65	LLLT increased total retraction by 1.08 mm (95% CI: 0.33 to 1.82); statistically significant	⨁◯◯◯^p^ Very low
	Changes in canine	position	2 RCTs	Serious	Very serious	Not serious	Serious	None	21	Mean differences from −0.13 mm (1st month) to −0.67 mm (4th month) favoring slightly less movement with LLLT, but all 95% CIs cross zero; no statistically significant effect	⨁◯◯◯^q^ Very low
	Canine retraction rate (mm/month)		6 RCTs	Serious	Not serious	Not serious	Serious	None	115	LLLT increased canine retraction rate by 0.66 mm/month compared to control; statistically significant (*p* < 0.0001)	⨁⨁◯◯^r^ Low
Accelerating lower canine retraction											
Using LLLT	Monthly retraction (mm)		2 RCTs	Serious	Serious	Not serious	Serious	None	22	1st month: MD ≈ +1.03 mm favoring LLLT (95% CI: 0.67 to 1.40) statistically significant 2nd month: MD ≈ −0.14 mm (95% CI: −0.80 to 0.51) not significant 3rd month: MD ≈ −0.06 mm (95% CI: −0.74 to 0.61) not significant	⨁⨁◯◯^s^ Low
	Canine retraction rate (mm/month)		3 RCTs	Serious	Not serious	Not serious	Serious	None	60	LLLT increased the canine retraction rate by 0.58 mm/month compared to the control. Statistically significant (*p* < 0.0001).	⨁⨁◯◯^t^ Low
Accelerating En‐masse retraction											
Using LLLT	Daily retraction (mm/day)		1 RCT	Serious	Not serious	Not serious	Serious	None	20	+30% faster tooth movement on laser side vs control over ~3 months (≈0.09 mm/day vs. 0.07 mm/day), statistically significant (*p* < 0.0001)	⨁⨁◯◯^u^ Low
	Monthly retraction (mm/month)		1 RCT	Serious	Not serious	Not serious	Very serious	None	12	0.694 mm/month (LLLT) vs. 0.500 mm/month (control). LLLT increased rate by 12.6%. The difference was statistically significant (*p* = 0.017).	⨁◯◯◯^v^ Very low
Using LED	Monthly retraction (mm/month)		1 RCT	Serious	Not serious	Not serious	Serious	None	60	Mean difference: +0.22 mm/month (LED group: 1.08 ± 0.54 vs. control: 0.86 ± 0.42) ➡ ~26% faster closure Statistically significant (*p* < 0.01)	⨁⨁◯◯^w^ Low
Accelerating upper molars intrusion											
Using LLLT	Average at six months in adults.		1 RCT	Serious	Not serious	Not serious	Serious	None	20	Mean intrusion at 6 months: PBM group: 2.31 ± 0.65 mm vs. Control: 2.95 ± 0.17 mm (slightly slower, no statistically significant difference, *p* > 0.05) ^∗∗^	⨁⨁◯◯^x^ Low
	Average at seven months in childs		1 RCT	Not serious	Not serious	Not serious	Serious	None	42	The mean intrusion was 1.21 mm in the FPBB + LLLT group vs. 0.82 mm in FPBB alone, a difference ≈ 0.39 mm more intrusion with LLLT, statistically significant (*p* = 0.018)	⨁⨁◯◯^y^ Low

*Note:* High‐quality, further research is very unlikely to change our confidence in the estimate of effect. Low quality, Further research is very likely to have an important impact on our confidence in the estimate of effect and is likely to change the estimate. Moderate quality, further research is likely to have an important impact on our confidence in the estimate of effect and may change the estimate. Very low quality, We are very uncertain about the estimate.

Abbreviations: CI, confidence interval; GRADE, Grading of Recommendations; Assessment; Development; and Evaluations; LAIP, leveling and alignment improvement percentage; LED, light‐emitting diode; LLLT, low‐level laser therapy; mm, millimeter; OLAT, orthodontic leveling and alignment time; PBM, photobiomodulation; RCTs, randomized clinical trials.

^a^Decline one level for risk of bias (some concern in the deviations from intended interventions [64, 66], measurement of the outcome [64], and selection of the reported result [64, 66]), and one level for Inconsistency.^3^

^b^Decline one level for risk of bias (some concern in the randomization process [65], deviations from intended interventions [65, 66], missing outcome data [65], and selection of the reported result [66]), and one level for Imprecision.^3^

^c^Decline two levels for risk of bias (some concern in the randomization process [67], missing outcome data [67], measurement of the outcome [67], and selection of the reported result [67], with high risk in deviations from intended interventions [67]), and one level for Imprecision.^3^

^d^Decline one level for risk of bias (some concern in the randomization process [69], and deviations from intended interventions [69]), and one level for Imprecision.^3^

^e^Decline one level for risk of bias (some concern in the randomization process [70], deviations from intended interventions [70], measurement of the outcome [70], and selection of the reported result [70], with high risk in missing outcome data [70]), and one level for Imprecision.^3^

^f^Decline one level for risk of bias (some concern in the randomization process [72], deviations from intended interventions [72], and selection of the reported result [72], with high risk in the measurement of the outcome [72]), and one level for Imprecision.^3^

^g^Decline one level for risk of bias (some concern in the randomization process [71], deviations from intended interventions [71], measurement of the outcome [71], and selection of the reported result [71]), and one level for Imprecision.^3^

^h^Decline one level for risk of bias (some concern in the deviations from intended interventions [74], and measurement of the outcome [74]), two levels for Indirectness^2^, and one level for Imprecision.^3^

^i^Decline one level for risk of bias (some concern in the deviations from intended interventions [75] and selection of the reported result [73]), one level for Inconsistency^1^, and one level for Imprecision.^3^

^j^Decline one level for risk of bias (some concern in the randomization process [79, 81, 82, 87, 96, 97], measurement of the outcome [81, 82, 87, 96], and selection of the reported result [79, 81, 96, 97]).

^k^Decline one level for risk of bias (some concern in the randomization process [79, 87, 96, 97], measurement of the outcome [87, 96], and selection of the reported result [79, 96, 97]), one level for Inconsistency^1^, and one level for Imprecision.^3^

^l^Decline one level for risk of bias (some concern in the randomization process [76], measurement of the outcome [76], and selection of the reported result [76]), one level for Inconsistency^1^, and one level for Imprecision.^3^

^m^Decline one level for risk of bias (some concern in the randomization process [77, 79, 81, 82, 84, 86, 87, 90, 92, 96–98], measurement of the outcome [81, 82, 87, 90, 96], and selection of the reported result [77, 79, 81, 84, 86, 90, 92, 96–98]), one level for Inconsistency^1^, and one level for Imprecision.^3^

^n^Decline one level for risk of bias (some concern in the randomization process [76, 77, 79, 81, 82, 86, 98], measurement of the outcome [76, 81, 82], and selection of the reported result [76, 77, 79, 81, 86, 98]), and one level for Inconsistency.^1^

^o^Decline one level for risk of bias (some concern in the randomization process [77, 79, 84, 94, 95], deviations from intended interventions [94, 95], measurement of the outcome [94], and selection of the reported result [77, 79, 84, 94, 95]), one level for Inconsistency^1^, and one level for Imprecision.^3^

^p^Decline one level for risk of bias (some concern in the randomization process [79, 94, 95, 98], deviations from intended interventions [94, 95], measurement of the outcome [94], and selection of the reported result [79, 94, 95, 98]), one level for Inconsistency^1^, and two levels for Imprecision.^3^

^q^Decline one level for risk of bias (some concern in the randomization process [87] and measurement of the outcome [87]), two levels for Inconsistency^1^, and one level for Imprecision.^3^

^r^Decline one level for risk of bias (some concern in the randomization process [78, 94, 95, 99, 100], deviations from intended interventions [74, 94, 95], measurement of the outcome [94], and selection of the reported result [74, 78, 94, 95, 99, 100]), and one level for Imprecision.^3^

^s^Decline one level for risk of bias (some concern in the randomization process [96, 97], measurement of the outcome [96], and selection of the reported result [96, 97]), one level for Inconsistency^1^, and one level for Imprecision.^3^

^t^Decline one level for risk of bias (some concern in the randomization process [95, 99, 100], deviations from intended interventions [95], and selection of the reported result [95, 99, 100]), and one level for Imprecision.^3^

^u^Decline one level for risk of bias (some concern in the randomization process [101], deviations from intended interventions [101], and selection of the reported result [101]), and one level for Imprecision.^3^

^v^Decline one level for risk of bias (some concern in the randomization process [102], deviations from intended interventions [102], measurement of the outcome [102], and selection of the reported result [102]), and two levels for Imprecision.^3^

^w^Decline one level for risk of bias (some concern in the randomization process [104], deviations from intended interventions [104], missing outcome data [104], and selection of the reported result [104]), and one level for Imprecision.^3^

^x^Decline one level for risk of bias (some concern in the randomization process [106], deviations from intended interventions [106], and selection of the reported result [106], with high risk of bias in the measurement of the outcome [106]), and one level for Imprecision.^3^

^y^Decline one level for Imprecision.^3^

^1^Wide variance of point estimates across studies Study.

^2^Indirectness: Short assessment duration (study time frame), and a difference between desired (Patient’ important) and measured outcomes.

^3^Imprecision: Limited number of trials and sample size.

**Table 10:** Summary of findings table according to the GRADE guidelines for the included trials on accelerating orthodontic treatment using BES.

Interventions		Quality assessment criteria						Summary of findings		
		No. of studies	Risk of bias	Inconsistency	Indirectness	Imprecision	Other considerations	No. of patients	Effect	Overall quality of evidence
Accelerating leveling and alignment										
Lower jaw without extraction			Serious	Not serious	Not serious	Serious	None	28	≈ 1.9 mm extra crowding reduction over 3 months (no statistically significant difference reported)	⨁⨁◯◯^a^ Low
Using BM		1 RCT								
Accelerating upper canine retraction										
Using LIDEC	Cumulative CR 1st month (mm from baseline)	1 RCT	Serious	Not serious	Not serious	Serious	None	7	Mean difference + 0.53 mm over 4 weeks (≈ 31 % faster); statistically significant (*p* = 0.001)	⨁⨁◯◯^b^ Low
Using PEMF	Total CR ((mm) post‐treatment)	2 RCTs	Serious	Not serious	Not serious	Serious	None	29	≈ +1.8 mm more retraction (≈ 35 % increase) *p* < 0.01 in both trials	⨁⨁◯◯^c^ Low

Abbreviations: BES, Bioelectric stimulation; BM, Bimaxillary mouthpiece; CR, Canine retraction; GRADE, Grading of Recommendations; Assessment; Development; and Evaluations; High‐quality, further research is very unlikely to change our confidence in the estimate of effect; LIDEC, Low‐intensity direct current; Low quality, further research is very likely to have an important impact on our confidence in the estimate of effect and is likely to change the estimate; mm, millimeter; Moderate quality, further research is likely to have an important impact on our confidence in the estimate of effect and may change the estimate; PEMF, Pulsed electromagnetic field; RCTs, randomized clinical trials; Very low quality, we are very uncertain about the estimate.

^a^Decline one level for risk of bias (some concern in the randomization process [108]), and one level for imprecision^3^.

^b^Decline one level for risk of bias (high risk in the randomization process [109], and some concern in the deviations from intended interventions [109], measurement of the outcome [109], and selection of the reported result [109]), and one level for Imprecision^3^.

^c^Decline one level for risk of bias (some concern in the randomization process [110, 111], deviations from intended interventions [110, 111], and selection of the reported result [110, 111], with high risk in the measurement of the outcome [110]), and one level for Imprecision^3^.

^1^Wide variance of point estimates across studies Study.

^2^Indirectness: Short assessment duration (study time frame), and a difference between desired (Patient’ important) and measured outcomes.

^3^Imprecision: Limited number of trials and sample size.

### 3.9. Additional Analysis

**Figure 13:**
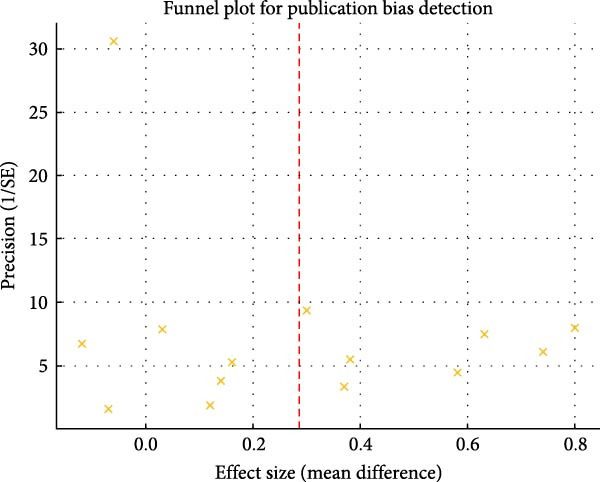
Funnel plot for publication bias detection. Cumulative upper canine retraction (mm) (at the first month) accelerated by low‐level laser therapy (LLLT).

Publication bias was visually assessed using funnel plots for outcomes reported in ≥10 studies. This included cumulative upper canine retraction (mm) from baseline using LLLT at 1 month. Figure 13 displays the funnel plot of effect estimate versus standard error (SE) for this outcome. The plot exhibited asymmetry, indicating publication bias.

Publication bias analysis was not possible for other interventions/variables due to insufficient trials.

## 4. Discussion

Despite the widespread use of PAIs to accelerate orthodontic treatment, evidence on their efficacy remains inconsistent due to variability in study designs and protocols. This umbrella review provided a critical synthesis and quantitative analysis of primary studies from existing systematic reviews to optimize PAIs’ use in clinical practice. It assessed the effects of PAIs on orthodontic treatment modalities (leveling and alignment, canine and En‐masse retraction, and molar intrusion). Overall, the certainty of evidence (GRADE) across modalities ranged from moderate to very low.

### 4.1. Leveling and Alignment

Current evidence shows that vibrational devices offer no clear therapeutic benefit in most orthodontic treatments [13, 26, 27]. In non‐extraction cases [36, 39, 41–43, 47, 48], AcceleDent’s effect is negligible, while devices like Tooth Masseuse [44] and Customized Appliance [45] produce only modest, non‐significant improvements, or benefits seen only in very small samples, limiting how broadly these results can be applied. Vibrations add no measurable advantage in extraction cases either [40]. This variation likely reflects insufficient force levels to trigger the needed biological response, inconsistent patient compliance, differences in treatment stages, and individual variability [28]. GRADE: ranged from moderate to low, for vibration devices.

PBM accelerates tooth movement by boosting cellular energy production and growth‐factor release, which speeds bone remodeling around the roots and shortens alignment time [34]. Clinically, lower‐arch leveling without extractions can be reduced by about 2 months with LLLT, and LED systems show similar, though wavelength‐dependent, effects. However, the early weeks of treatment do not show an immediate rise in the Leveling and Alignment Improvement Percentage index (LAIP) [65, 66], since final precision still depends on appliance design and applied mechanical forces.

LED treatment has been found to cut processing time by roughly 3 weeks in non‐extraction lower arches [67] and by nearly 2 months in upper‐extraction cases [69]. Its broader beam angle and adequate soft‐tissue penetration in the upper arch likely contribute to these gains. LED can increase tooth‐movement rates by over 0.5 mm per week, helping achieve treatment goals more quickly.

LLLT also reduces the required daily wear time for clear aligners from 22 to 12 h by stimulating bone‐forming cells and reducing local inflammation [72]. This makes aligner compliance easier for patients, improving success rates, lowering compliance‐related risks, and shortening overall treatment duration. GRADE: ranged from low to very low, for PBM (LLLT/LED).

BES: In one small RCT [108], BES reduced lower‐arch crowding by 1.9 mm over 3 months, but the tiny sample size limits result credibility. BES likely works by activating osteoblasts through ion‐channel stimulation, improving local blood flow and calcium concentration, thus enhancing bone remodeling and lowering tissue resistance. However, it remains unclear whether the effect accelerates incisor movement or alters tooth axis, raising questions about its precision. These early findings are promising and justify further study, but they do not yet support strong clinical recommendations. GRADE: low for BES.

From a clinical standpoint, these findings indicate that vibration alone is unlikely to justify additional chairside time or device cost for routine leveling and alignment, whereas PBM—particularly LLLT—may be selectively considered in cases where a 3–8‐week reduction in alignment time or a halving of aligner wear (from 22 to 12 h/day) could meaningfully improve adherence, although the overall certainty of evidence remains low.

### 4.2. Canine Retraction

Canine retraction adjunctive methods exhibit variable efficacy depending on their biological mechanisms of action and implementation quality:

Vibrational Stimulation: Significant superiority using AcceleDent (0.27 mm/month) arises from osteocyte activation via enhanced blood perfusion and increased cytokines [13]. In contrast, manual brushing showed irregular cumulative effects (likely due to inconsistent stimulation or patient compliance variability) [54, 56, 57]. However, the wide CI (0.01–1.32) highlights the need for standardized protocols (e.g., vibration intensity/duration). GRADE: very low for vibration devices.

Photobiostimulation (LLLT and LED): Delayed results (up to the fourth month) correlate with cumulative cellular modifications (e.g., increased ATP production and growth factors like Vascular Endothelial Growth Factor [VEGF]) [87]. The weaker outcomes with LED devices may reflect differences in wavelength penetration [73, 75]. The decline in mandibular stimulation efficacy could stem from high bone density resisting rapid changes or gradual depletion of cellular metabolic reserves, necessitating intermittent protocols to avoid adaptive resistance [96]. GRADE: ranged from moderate to very low for LLLT and from low to very low for LED.

BES: Marked superiority (1.78 mm) reflects activation of calcium channels and Wnt/*β*‐catenin signaling pathways that accelerate bone remodeling [110, 111]. However, the scarcity of studies and variability in their designs (e.g., stimulation intensity) limit the generalizability of findings. GRADE: low for BES.

Clinically, a gain of roughly 0.27–0.66 mm/month in canine retraction corresponds to ~2–4 mm of additional movement over a 6‐month retraction phase, which may shorten space closure by one or two visits but is unlikely to dramatically reduce total treatment time, whereas the larger point estimate reported for BES (≈1.8 mm at 3 months) remains too uncertain to support routine use until replicated in larger, high‐quality trials.

### 4.3. En‐Masse Retraction

Vibration devices showed no meaningful benefit, likely due to insufficient amplitude or non‐optimal frequencies failing to stimulate the cellular mechanisms of bone remodeling, compounded by uneven force distribution across arches [4, 63]. GRADE: ranged from low to very low, for vibration devices.

In contrast, both LLLT and LED treatments produced statistically significant increases in daily and monthly tooth movement [101, 104], reflecting enhanced cytokine and growth‐factor signaling that accelerates bone resorption and formation. However, these results stem from single, small RCTs, limiting their generalizability. Future work should optimize vibratory device design and standardize PBM protocols—especially dosing and delivery—to reliably harness their respective effects in a safe, efficient orthodontic regimen. GRADE: ranged from low to very low for LLLT, and low for LED.

In routine practice, the small absolute gains reported with PBM during en‐masse retraction (on the order of fractions of a millimeter to just over 1 mm per month) are unlikely to transform overall treatment duration, but could marginally shorten the retraction phase in complex extraction cases; in contrast, vibration devices currently offer no clinically meaningful advantage in this context and should not be relied upon as primary accelerators.

### 4.4. Upper Molars Intrusion

In adults, LLLT did not achieve a statistically significant effect over 6 months, possibly due to slow bone remodeling and high bone density [106]. In children, it showed a positive, statistically significant effect after 7 months, reflecting more active bone growth and faster renewal [107]. The absence of CIs, a limited number of studies, and a small sample size limit the ability to generalize the results. GRADE: low for LLLT.

Thus, for molar intrusion, PBM should presently be regarded as an experimental adjunct with limited clinical impact in adults and only a modest, age‐dependent benefit in growing patients, where an additional ≈0.4 mm of intrusion over 7 months is unlikely to alter treatment planning but might slightly reduce the duration of active mechanics.

### 4.5. Evidence Comparison: Systematic Reviews vs. Pooled Primary Data

Discrepancies between the results reported in the included systematic reviews and those of the current umbrella review (after consolidating primary studies) arise due to:

1. Limited primary studies per review: The largest review included only 25 trials.
2. Narrative reporting: Outcomes were often described qualitatively, lacking quantitative effect size analysis relative to study quality/quantity.
3. Heterogeneous meta‐analyses: Some reviews combined diverse assessment methods, acceleration techniques, parameters, or protocols without subgroup analyses or appropriate statistical models (fixed‐/random‐effects), risking biased effect estimates.

When these effect sizes are considered together, they suggest that—even under favorable conditions—physical adjunctive interventions are unlikely to reduce the overall duration of comprehensive orthodontic treatment by more than a few months or to eliminate multiple visits; rather, their benefits are concentrated in specific phases (such as early alignment or initial space closure) and remain highly dependent on protocol design and patient adherence. Consequently, PAIs should be presented to patients as optional adjuncts that may offer modest, phase‐specific time savings or improved comfort in selected cases, rather than as guaranteed solutions for dramatically shortening orthodontic therapy.

### 4.6. Strengths and Limitations

This umbrella review’s main benefit is to summarize evidence on physical interventions for accelerating orthodontic treatment and assess its certainty. Strengths include: an extensive electronic search including gray literature, dissertations, and non‐English references. Reviews quality and bias were checked with AMSTAR‐2 and ROBIS. Primary studies were extracted and re‐assessed for bias using ROB2. Evidence quality was graded using GRADE. Systematic review findings were compared with the pooled primary studies’ results.

Key limitations include significant clinical heterogeneity across reviews and primary trials due to variations in PAIs, parameters, appliances, treatments, wire sequences, and protocols. This was mitigated by grouping data with similar treatment goals, PAIs, and parameters. Furthermore, many primary studies exhibited low quality with high/unclear risk of bias, addressed through ROB2 re‐evaluation and GRADE evidence assessment. Substantial overlap among systematic reviews introduced redundancy, as many reiterated the same trials without novel insights. To prevent this, orthodontic journals should adopt Cochrane’s model of updating existing reviews rather than publishing redundant ones. Additionally, the present umbrella review was intentionally focused on efficacy outcomes (rate of tooth movement and treatment duration), without a parallel, comprehensive synthesis of key safety endpoints such as pain and root resorption; these outcomes are being addressed in a separate, complementary review, and future syntheses should incorporate standardized safety measures to support a more balanced risk–benefit appraisal of physical adjunctive interventions. Finally, future trials should prioritize robust RCTs with larger samples, long‐term follow‐up, standardized protocols, and thorough evaluation of interventions’ cost–benefit‐risk profiles.

### 4.7. Future Research Recommendations and Gaps

Future trials should use preregistered protocols and report according to CONSORT. Randomization, allocation concealment, and blinding must be explicit, with credible sham devices and adherence reporting. Multicenter RCTs with adequate sample sizes are encouraged. A core outcome set should include OTM rate and total treatment time, with standardized measurement methods and 95% CIs. Adverse events and patient‐reported outcomes should be captured, and basic economic endpoints added when feasible. Dosing and parameters must be standardized for each modality: for light‐based therapy, report wavelength, energy density, power, application points, and session timing; for vibration, report frequency, force, session length, and use patterns; for electrical or electromagnetic stimulation, report current or field strength, waveform, duty cycle, and exposure time. Co‐interventions should be minimized or described clearly, and dissimilar modalities should not be pooled in single analyses. De‐identified data and analysis code should be shared, and follow‐up extended to assess durability and late harms. Adhering to these principles will reduce heterogeneity, increase certainty, and enable reliable, modality‐specific conclusions.

## 5. Conclusions

This umbrella review reveals inconsistent quality in included systematic reviews, with all exhibiting issues in at least one domain (ROB, AMSTAR‐2, or ROBIS). Consequently, their evidence credibility remains questionable and requires cautious interpretation.

After standardizing primary study criteria and removing overlaps, pooled data show orthodontic acceleration effectiveness varies by each technique’s mechanism.

### 5.1. Vibrational Devices

Current data do not support vibration as a primary acceleration method. It may, however, complement other modalities (e.g., PBM) if patients are well‐informed about its modest effect and maintain strict adherence. Despite widespread marketing, vibration currently holds the weakest scientific support among acceleration strategies. GRADE: ranged from moderate to very low.

### 5.2. PBM

PBM shows potential in accelerating treatment, particularly during leveling/alignment and retraction phases. Its efficacy appears influenced by treatment stage, arch location, extraction status, and light source (LLLT vs. LED). LLLT generally shows stronger outcomes than LED, with effects often more pronounced in the upper arch. Consistent protocols are essential to optimize results. GRADE: overall ranged from moderate to very low; LLLT: ranged from moderate to very low; LED: ranged from low to very low.

### 5.3. BES

Preliminary evidence for techniques like PEMF is promising, especially for canine retraction, though further validation is needed. Combining PBM and BES in future multicenter trials using standardized protocols and CBCT‐based assessments is encouraged. GRADE: low.

Finally, studies assessing cost‐effectiveness and patient compliance are crucial to guide practical adoption and ensure long‐term sustainability of these technologies.

**Nomenclature**
AMSTAR‐2: A Measurement Tool to Assess Systematic Reviews‐2
ATP: Adenosine Triphosphate
BES: Bioelectric Stimulation
BM: Bimaxillary mouthpiece
CCA: Corrected Covered Area
CBCT: Cone Beam Computed Tomography
CI: Confidence Interval
CIs: Confidence Intervals
GRADE: Grading of Recommendations Assessment, Development, and Evaluation
GROOVE: Graphical Representation of Overlap for Overviews
LAIP: Leveling and Alignment Improvement Percentage
LED: Light‐Emitting Diode
LIDEC: Low‐intensity direct current
LIES: Low‐Intensity Electrical Stimulation
LII: Little’s Irregularity Index
LIPUS: Low‐Intensity Pulsed Ultrasound
LLLT: Low‐Level Laser Therapy
MA‐Pool: Meta‐Analysis of Pooled Data
MD: Mean Difference
MeSH: Medical Subject Headings
MV: Mechanical Vibration
OLAT: Orthodontic Leveling and Alignment Time
OTM: Orthodontic Tooth Movement
PAIs: Physical Adjunctive Interventions
PBM: Photobiomodulation
PEMF: Pulsed Electromagnetic Fields
PICOS: Participants, Interventions, Comparisons, Outcomes, Study Design
PRISMA: Preferred Reporting Items for Systematic Reviews and Meta‐Analyses
PROSPERO: International Prospective Register of Systematic Reviews
RANKL: Receptor Activator of Nuclear Factor Kappa‐B Ligand
RCTs: Randomized Controlled Trials
ROBIS: Risk Of Bias in Systematic Reviews
RoB 2: Cochrane Risk of Bias 2 Tool
SMD: Standardized Mean Difference
SRs: Systematic Reviews
VEGF: Vascular Endothelial Growth Factor
VDs: Vibrational Devices
WMD: Weighted Mean Difference.

## Ethics Statement

This umbrella review and meta‐analysis is based solely on previously published studies and involves no new data collection from human participants or animals; therefore, ethics approval and informed consent were not required (not applicable).

## Disclosure

All authors have read and approved the final version of the manuscript and agree to be accountable for all aspects of the work.

## Conflicts of Interest

The authors declare no conflicts of interest.

## Author Contributions

Conceptualization: Mohamad Radwan Sirri, Mohammad Osama Namera. Methodology: Mohamad Radwan Sirri, Mohammad Osama Namera, Mohamad Yaman Salahi Alasbahi. Software: Mohamad Yaman Salahi Alasbahi. Validation: Mohamad Radwan Sirri, Mohammad Osama Namera, Mohamad Yaman Salahi Alasbahi. Formal analysis: Mohamad Radwan Sirri, Salar Karim Khalil. Investigation: Mohamad Yaman Salahi Alasbahi. Data curation: Mohamad Yaman Salahi Alasbahi, Salar Karim Khalil. Writing – original draft: Mohamad Radwan Sirri, Mohamad Yaman Salahi Alasbahi, Salar Karim Khalil. Writing – review & editing: Mohammad Osama Namera, Salar Karim Khalil, Mohamad Radwan Sirri. Visualization: Mohamad Radwan Sirri, Mohammad Osama Namera. Supervision: Mohamad Radwan Sirri, Mohammad Osama Namera. Project administration: Mohamad Radwan Sirri.

## Funding

No funding was received for this research.

## Supporting Information

Additional supporting information can be found online in the Supporting Information section.

## Supporting information


**Supporting Information 1** Table S1: Electronic search strategy.

**Supporting Information 2** Table S2: The CCA formula and AMSTAR‐2 tool domains and judgments.

**Supporting Information 3** Table S3: Studies excluded and reasons for exclusion.

**Supporting Information 4** Table S4: Characteristics of the pooled primary studies on accelerating orthodontic treatment using VDs.

**Supporting Information 5** Table S5: Characteristics of the pooled primary studies on accelerating orthodontic treatment using PBM.

**Supporting Information 6** Table S6: Characteristics of the pooled primary studies on accelerating orthodontic treatment using BES.

**Supporting Information 7** Table S7: The degree of overlap of primary studies included in all systematic reviews.

**Supporting Information 8** Table S8: The degree of overlap of primary studies included in systematic reviews of VDs.

**Supporting Information 9** Table S9: The degree of overlap of primary studies included in systematic reviews of PBM.

**Supporting Information 10** Table S10: Detailed supporting reasons for the AMSTAR‐2 (A Measurement Tool to Assess Systematic Reviews‐2) assessment of each included systematic review.

**Supporting Information 11** Table S11: Detailed supporting reasons for the ROBIS Tool assessment of each included systematic review.

**Supporting Information 12** Table S12: The need for reassessment of risk of bias in primary studies using ROB2 tool.

**Supporting Information 13** Table S13: Risk‐of‐bias reassessment of included RCTs with supporting justifications.

**Supporting Information 14** Figure S1: Risk of bias summary of RCT: reviewers’ reassessment of risk of bias for each domain in the included studies using the RoB 2 Tool.

**Supporting Information 15** Figure S2: Overall reassessment of risk of bias profile for RCTs: Reviewers’ RoB 2 judgments presented as percentages across all included studies.

## Data Availability

The data that support the findings of this study are available from the corresponding author upon reasonable request.
