# Supplementary material for: The Effectiveness of Physical Adjunctive Interventions in the Acceleration of Orthodontic Tooth Movement: An Umbrella Review and Meta‐Analysis
Source: Int J Dent. 2026 Feb 3;2026:9131541. doi: 10.1155/ijod/9131541 (PMC12868923; doi:10.1155/ijod/9131541)
Supplement: Supplementary file 1 — Supporting Information 1 Table S1: Electronic search strategy. [file IJOD-2026-9131541-s008.docx]

| **Supplementary Table 1.** Electronic search strategy | | |
| --- | --- | --- |
| NO | Database | Search strategy |
| **1** | **PubMed**  Publication Date:  from no limit until 25-3-2025 | 1. orthodontic* OR ‘‘tooth movement’’ OR ‘’dental movement’’ OR ‘‘orthodontic treatment’’ 2. accelerate* OR rapid* OR short* OR speed* OR duration OR rate OR ‘‘regional accelerated phenomenon’’ OR RAP. 3. (non-surgical* OR physical intervention*) AND (Vibration* OR Mechanical Vibration OR photobiostimulation* OR laser therapy OR low-level laser OR low-level laser therapy OR LLLT OR ‘’light emitting diode’’ OR LED OR Bioelectric Stimulation* OR ‘’Electric current’’ OR ‘’Electric Stimulation’’ OR ‘’Low Intensity Direct Electric Current’’ OR LIDC OR ‘’Low-Intensity Electrical Stimulation’’ OR LIES OR ‘’low-intensity pulsed ultrasound’’ OR LIPUS OR ‘’pulsed electromagnetic field’’ OR PEMF) 4. ‘’Systematic review’’ OR “meta-analysis” 5. #1 AND #2 AND #3 AND #4 |
| **2** | **Web of Science**  Publication Date:  from no limit until 25-3-2025 | 1. **TS**= (orthodontic* OR ‘‘tooth movement’’ OR ‘’dental movement’’ OR ‘‘orthodontic treatment’’) 2. **TS**= (accelerate* OR rapid* OR short* OR speed* OR duration OR rate OR ‘‘regional accelerated phenomenon’’ OR RAP) 3. **TS**= (non-surgical* OR physical intervention*) AND (Vibration* OR Mechanical Vibration OR photobiostimulation* OR laser therapy OR low-level laser OR low-level laser therapy OR LLLT OR ‘’light emitting diode’’ OR LED OR Bioelectric Stimulation* OR ‘’Electric current’’ OR ‘’Electric Stimulation’’ OR ‘’Low Intensity Direct Electric Current’’ OR LIDC OR ‘’Low-Intensity Electrical Stimulation’’ OR LIES OR ‘’low-intensity pulsed ultrasound’’ OR LIPUS OR ‘’pulsed electromagnetic field’’ OR PEMF) 4. **TS**= (‘’Systematic review’’ OR “meta-analysis”) 5. #4 #1 AND #2 AND #3 AND #4 |
| **3** | **Scopus**  Publication Date:  from no limit until 25-3-2025 | 1. **TITLE-ABS-KEY** (orthodontic* OR ‘‘tooth movement’’ OR ‘’dental movement’’ OR ‘‘orthodontic treatment’’) 2. **TITLE-ABS-KEY** (accelerate* OR rapid* OR short* OR speed* OR duration OR rate OR ‘‘regional accelerated phenomenon’’ OR RAP) 3. **TITLE-ABS-KEY** (non-surgical* OR physical intervention*) AND (Vibration* OR Mechanical Vibration OR photobiostimulation* OR laser therapy OR low-level laser OR low-level laser therapy OR LLLT OR ‘’light emitting diode’’ OR LED OR Bioelectric Stimulation* OR ‘’Electric current’’ OR ‘’Electric Stimulation’’ OR ‘’Low Intensity Direct Electric Current’’ OR LIDC OR ‘’Low-Intensity Electrical Stimulation’’ OR LIES OR ‘’low-intensity pulsed ultrasound’’ OR LIPUS OR ‘’pulsed electromagnetic field’’ OR PEMF) 4. **TITLE-ABS-KEY** (‘’Systematic review’’ OR “meta-analysis”) 5. #1 AND #2 AND #3 AND #4 |
| **4** | **Cochrane Central Register of Controlled Trials (CENTRAL)**  Publication Date:  from no limit until 25-3-2025  Search field:  title, abstract,  keywords | 1. orthodontic* OR ‘‘tooth movement’’ OR ‘’dental movement’’ OR ‘‘orthodontic treatment’’ 2. accelerate* OR rapid* OR short* OR speed* OR duration OR rate OR ‘‘regional accelerated phenomenon’’ OR RAP. 3. (non-surgical* OR physical intervention*) AND (Vibration* OR Mechanical Vibration OR photobiostimulation* OR laser therapy OR low-level laser OR low-level laser therapy OR LLLT OR ‘’light emitting diode’’ OR LED OR Bioelectric Stimulation* OR ‘’Electric current’’ OR ‘’Electric Stimulation’’ OR ‘’Low Intensity Direct Electric Current’’ OR LIDC OR ‘’Low-Intensity Electrical Stimulation’’ OR LIES OR ‘’low-intensity pulsed ultrasound’’ OR LIPUS OR ‘’pulsed electromagnetic field’’ OR PEMF) 4. ‘’Systematic review’’ OR “meta-analysis” 5. #1 AND #2 AND #3 AND #4 |
| **5** | **OpenGrey**  **(via DANS EASY)** | 1. (orthodontic* OR ‘‘tooth movement’’ OR ‘’dental movement’’ OR ‘‘orthodontic treatment’’) AND (accelerate* OR rapid* OR short* OR speed* OR duration OR rate OR ‘‘regional accelerated phenomenon’’ OR RAP) 2. (non-surgical* OR physical intervention*) AND (Vibration* OR Mechanical Vibration OR photobiostimulation* OR laser therapy OR low-level laser OR low-level laser therapy OR LLLT OR ‘’light emitting diode’’ OR LED OR Bioelectric Stimulation* OR ‘’Electric current’’ OR ‘’Electric Stimulation’’ OR ‘’Low Intensity Direct Electric Current’’ OR LIDC OR ‘’Low-Intensity Electrical Stimulation’’ OR LIES OR ‘’low-intensity pulsed ultrasound’’ OR LIPUS OR ‘’pulsed electromagnetic field’’ OR PEMF) 3. (‘’Systematic review’’ OR “meta-analysis”) |
| **6** | **PQDT OPEN (from proQuest)** http://pqdtopen.proquest.com/ | (orthodontic OR tooth movement OR dental movement OR orthodontic treatment) AND (accelerate OR rapid OR short OR speed OR duration OR rate OR regional accelerated phenomenon OR RAP) AND (flapless OR minimally invasive OR noninvasive) AND (non-surgical OR physical intervention) AND (Vibration OR Mechanical Vibration OR photobiostimulation OR laser therapy OR low-level laser OR low-level laser therapy OR LLLT OR light emitting diode OR LED OR Bioelectric Stimulation OR Electric current OR Electric Stimulation OR Low Intensity Direct Electric Current OR LIDC OR Low-Intensity Electrical Stimulation OR LIES OR low-intensity pulsed ultrasound OR LIPUS OR pulsed electromagnetic field OR PEMF) AND (Systematic review OR meta-analysis) |
