# Supplementary material for: The Effectiveness of Physical Adjunctive Interventions in the Acceleration of Orthodontic Tooth Movement: An Umbrella Review and Meta‐Analysis
Source: Int J Dent. 2026 Feb 3;2026:9131541. doi: 10.1155/ijod/9131541 (PMC12868923; doi:10.1155/ijod/9131541)
Supplement: Supplementary file 2 — Supporting Information 2 Table S2: The CCA formula and AMSTAR‐2 tool domains and judgments. [file IJOD-2026-9131541-s007.docx]

| **Supplementary Table 2: The CCA formula, AMSTAR-2 tool domains, and ROBIS tool phases and judgments** | | | | | | | | |
| --- | --- | --- | --- | --- | --- | --- | --- | --- |
| **Domains** | | | | **Judgments** | | | | |
| **CCA (For Overlap Assessment)** | | | | | | | | |
| 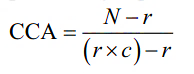 | | | | **0–5 (Slight)**  **6–10 (Moderate)**  **11–15 (High)**  **>15 (Very High Overlap)** | | | | |
| **AMSTAR-2: A Measurement Tool to Assess Systematic Reviews-2 tool (For Quality Assessment of SRs)** | | | | | | | | |
| 1. Did the research questions and inclusion criteria for the review include the components of PICO? | | | | **Yes, No** | | **Over All** | **HC**  **MC**  **LC**  **CLC** |  |
| 1. Did the report of the review contain an explicit statement that the review methods were established prior to the conduct of the review and did the report justify any significant deviations from the protocol? | | | | **Yes, Partial Yes, No** | |  |  |  |
| 1. Did the review authors explain their selection of the study designs for inclusion in the review? | | | | **Yes, No** | |  |  |  |
| 1. Did the review authors use a comprehensive literature search strategy? | | | | **Yes, Partial Yes, No** | |  |  |  |
| 1. Did the review authors perform study selection in duplicate? | | | | **Yes, No** | |  |  |  |
| 1. Did the review authors perform data extraction in duplicate? | | | | **Yes, No** | |  |  |  |
| 1. Did the review authors provide a list of excluded studies and justify the exclusions? | | | | **Yes, Partial Yes, No** | |  |  |  |
| 1. Did the review authors describe the included studies in adequate detail? | | | | **Yes, Partial Yes, No** | |  |  |  |
| 1. Did the review authors use a satisfactory technique for assessing the risk of bias (RoB) in individual studies that were included in the review? | | | | **Yes, Partial Yes, No** | |  |  |  |
| 1. Did the review authors report on the sources of funding for the studies included in the review? | | | | **Yes, No** | |  |  |  |
| 1. If meta-analysis was performed did the review authors use appropriate methods for statistical combination of results? | | | | **Yes, No, No MA conducted** | |  |  |  |
| 1. If meta-analysis was performed, did the review authors assess the potential impact of RoB in individual studies on the results of the meta-analysis or other evidence synthesis? | | | | **Yes, No, No MA conducted** | |  |  |  |
| 1. Did the review authors account for RoB in individual studies when interpreting/ discussing the results of the review? | | | | **Yes, No** | |  |  |  |
| 1. Did the review authors provide a satisfactory explanation for, and discussion of, any heterogeneity observed in the results of the review? | | | | **Yes, No** | |  |  |  |
| 1. If they performed quantitative synthesis did the review authors carry out an adequate investigation of publication bias (small study bias) and discuss its likely impact on the results of the review? | | | | **Yes, No, No MA conducted** | |  |  |  |
| 1. Did the review authors report any potential sources of conflict of interest, including any funding they received for conducting the review? | | | | **Yes, No** | |  |  |  |
| **ROBIS: risk of bias in systematic reviews** | | | | | | | | |
| **Phase 2: Identifying concerns with the review process** | | | | **Phase 3: Final Risk of Bias Judgment** | | **Overall** | **LROB**  **UNROB**  **HROB** |  |
| **1. Study Eligibility Criteria** | 1.1 Did the review adhere to pre-defined objectives and eligibility criteria?  1.2 Were the eligibility criteria appropriate for the review question?  1.3 Were eligibility criteria unambiguous?  1.4 Were any restrictions in eligibility criteria based on study characteristics appropriate (e.g. date, sample size, study quality, outcomes measured)?  1.5 Were any restrictions in eligibility criteria based on sources of information appropriate (e.g. publication status or format, language, availability of data)? | Y/PY/PN/N/NI | Low/High/  Unclear | A. Addressed Phase 2 concerns?  B. Relevance of studies considered?  C. Avoided overemphasis on significance? | Y/PY/PN/N/NI |  |  |  |
| **2. Study Identification & Selection** | 2.1 Did the search include an appropriate range of databases/electronic sources for published and unpublished reports?  2.2 Were methods additional to database searching used to identify relevant reports?  2.3 Were the terms and structure of the search strategy likely to retrieve as many eligible studies as possible?  2.4 Were restrictions based on date, publication format, or language appropriate?  2.5 Were efforts made to minimize error in the selection of studies? | Y/PY/PN/N/NI | Low/High/  Unclear |  |  |  |  |  |
| **3. Data Collection & Appraisal** | 3.1 Were efforts made to minimize error in data collection?  3.2 Were sufficient study characteristics available for both review authors and readers to be able to interpret the results?  3.3 Were all relevant study results collected for use in the synthesis?  3.4 Was risk of bias (or methodological quality) formally assessed using appropriate criteria?  3.5 Were efforts made to minimize error in risk of bias assessment? | Y/PY/PN/N/NI | Low/High/  Unclear |  |  |  |  |  |
| **4. Synthesis & Findings** | 4.1 Did the synthesis include all studies that it should?  4.2 Were all pre-defined analyses reported or departures explained?  4.3 Was the synthesis appropriate given the nature and similarity in the research questions, study designs, and outcomes across included studies?  4.4 Was between-study variation (heterogeneity) minimal or addressed in the synthesis?  4.5 Were the findings robust, e.g., as demonstrated through funnel plot or sensitivity analyses?  4.6 Were biases in primary studies minimal or addressed in the synthesis? | Y/PY/PN/N/NI | Low/High/Unclear |  |  |  |  |  |
| **CCA**: Corrected Covered Areas; **N**: number of included publications; **r**: number of rows; **c**: number of columns.  **AMSTAR**: A Measurement Tool to Assess Systematic Reviews; **SRs**: Systematic reviews; **MA**: Meta-Analysis.  **HC**: High Confidence:   - No non-critical weaknesses, or only one non-critical weakness. - Results align closely with an ideal systematic review.   **MC**: Moderate Confidence:   - More than one non-critical weakness. - Results are acceptable but with non-critical limitations.   **LC**: Low Confidence:   - One critical flaw (with or without non-critical weaknesses). - Likely significant negative impact on results.   **CLC**: Critically Low Confidence:   - More than one critical flaw (with or without non-critical weaknesses). - Results are fundamentally unreliable.   **Critical Items** in AMSTAR-2: Q 2 -4 -7 - 9 -11 - 13 – 15  **ROBIS:** risk of bias in systematic reviews; **Y**: Yes; **PY**: Probably Yes; **PN**: Probably No; **N**: No; **NI**: No Information  **Evaluation Criteria for Phase 2 questions:**   - LOW: Majority Y/PY. - HIGH: Multiple PN/N. - UNCLEAR: Insufficient info (NI).   **Final Risk Level**   - **LROB**: Low Risk of Bias   Reliability: Review findings are trustworthy.  Reason: No Phase 2 concerns, or concerns were addressed appropriately.  Conclusions: Evidence-backed and considered study relevance.   - **HROB**: High Risk of Bias   Weaknesses: Unresolved Phase 2 issues, unsupported conclusions, or ignored study relevance.   - **UNROB**: Unclear Risk of Bias   Reason: Insufficient data to assess bias. | | | | | | | | |
