# Supplementary material for: The Effectiveness of Physical Adjunctive Interventions in the Acceleration of Orthodontic Tooth Movement: An Umbrella Review and Meta‐Analysis
Source: Int J Dent. 2026 Feb 3;2026:9131541. doi: 10.1155/ijod/9131541 (PMC12868923; doi:10.1155/ijod/9131541)
Supplement: Supplementary file 3 — Supporting Information 3 Table S3: Studies excluded and reasons for exclusion. [file IJOD-2026-9131541-s006.docx]

| **Supplementary Table 3: Studies excluded and reasons for exclusion** | | |
| --- | --- | --- |
| **NO** | **Study** | **Reason for exclusion** |
| **1** | Akbari A, Gandhi V, Chen J, et al.; Vibrational Force on Accelerating Orthodontic Tooth Movement: A Systematic Review and Meta-Analysis. European Journal of Dentistry 2022;17. doi: 10.1055/s-0042-1758070. | CCTs included |
| **2** | Al-Shahrani I, Togoo R, Hosmani J; Photobiomodulation in Acceleration of Orthodontic Tooth Movement: A Systematic Review and Meta analysis. Complementary Therapies in Medicine 2019;47:102220. doi: 10.1016/j.ctim.2019.102220. |  |
| **3** | Baghizadeh Fini M, Olyaee P, Homayouni A; The Effect of Low-Level Laser Therapy on the Acceleration of Orthodontic Tooth Movement. J Lasers Med Sci 2020;11(2):204-211. doi: 10.34172/jlms.2020.34. |  |
| **4** | Farsaii A, Al-Jewair T; Declarative Title: Insufficient evidence supports the use of low level laser therapy (LLLT) to accelerate tooth movement, prevent orthodontic relapse, and modulate acute pain during orthodontic treatment. Journal of Evidence Based Dental Practice 2017;17. doi: 10.1016/j.jebdp.2017.06.008. |  |
| **5** | Ge M, He W, Chen J, et al.; Efficacy of low-level laser therapy for accelerating tooth movement during orthodontic treatment: a systematic review and meta-analysis. Lasers in medical science 2014;30. doi: 10.1007/s10103-014-1538-z. |  |
| **6** | Huang T, Wang Z, Li J; Efficiency of photobiomodulation on accelerating the tooth movement in the alignment phase of orthodontic treatment—A systematic review and meta-analysis. Heliyon 2023;9:e13220. doi: 10.1016/j.heliyon.2023.e13220. |  |
| **7** | Jedliński M, Romeo U, Del Vecchio A, et al.; Comparison of the Effects of Photobiomodulation with Different Lasers on Orthodontic Movement and Reduction of the Treatment Time with Fixed Appliances in Novel Scientific Reports: A Systematic Review with Meta-Analysis. Photobiomodulation, Photomedicine, and Laser Surgery 2020;38. doi: 10.1089/photob.2019.4779. |  |
| **8** | Long H, Zhou Y, Xue J, et al.; The effectiveness of low-level laser therapy in accelerating orthodontic tooth movement: a meta-analysis. Lasers in medical science 2013;30. doi: 10.1007/s10103-013-1507-y. |  |
| **9** | Mourad J; Evaluating Non-Surgical Methods of Accelerating the Rate of Orthodontic Tooth Movement: A Systematic Review. Boston University, 2024. |  |
| **10** | Olmedo-Hernández O, Mota-Rodríguez A, Torres-Rosas R, et al.; Effect of the photobiomodulation for acceleration of the orthodontic tooth movement: a systematic review and meta‑analysis. Lasers in Medical Science 2022;37. doi: 10.1007/s10103-022-03538-8. |  |
| **11** | Sousa M, Pinzan A, Consolaro A, et al.; Systematic Literature Review: Influence of Low-Level Laser on Orthodontic Movement and Pain Control in Humans. Photomedicine and laser surgery 2014;32. doi: 10.1089/pho.2014.3789. |  |
| **12** | Yavagal C, Matondkar S, Yavagal P; Efficacy of Laser Photobiomodulation in Accelerating Orthodontic Tooth Movement in Children: A Systematic Review with Meta-analysis. International Journal of Clinical Pediatric Dentistry 2021;14:S91-S97. doi: 10.5005/jp-journals-10005-1964. |  |
| **13** | Zheng D-H, Du Y-Q, Zhang Q-Q, et al.; Effect of low-level laser therapy on orthodontic dental alignment: a systematic review and meta-analysis. Lasers in Medical Science 2023;38. doi: 10.1007/s10103-023-03835-w. |  |
| **14** | Chaturvedi T; Effect of Electrical Stimulation on Orthodontic Tooth Movement: A Systematic Review. 2020. | Animal Studies included |
| **15** | Pascoal S, Oliveira S, Ascione M, et al.; Effects of Vibration on Accelerating Orthodontic Tooth Movement in Clinical and In Vivo Studies: A Systematic Review. Dentistry Journal 2024;12:243. doi: 10.3390/dj12080243. |  |
| **16** | Cronshaw M, Parker S, Anagnostaki E, et al.; Systematic Review of Orthodontic Treatment Management with Photobiomodulation Therapy. Photobiomodulation, Photomedicine, and Laser Surgery 2019;37. doi: 10.1089/photob.2019.4702. | Did not report the risk of bias test. |
| **17** | Jing D, Xiao J, Li X, et al.; The effectiveness of vibrational stimulus to accelerate orthodontic tooth movement: a systematic review. BMC Oral Health 2017;17(1):143. doi: 10.1186/s12903-017-0437-7. | Includes studies on the side effects of vibration without information on acceleration. |
| **18** | El-Angbawi A, McIntyre GT, Fleming PS, et al.; Non-surgical adjunctive interventions for accelerating tooth movement in patients undergoing fixed orthodontic treatment. Cochrane Database Syst Rev 2015;2015(11):Cd010887. doi: 10.1002/14651858.CD010887.pub2. | Updated in 2023; the updated version is included in this review |
