# Supplementary material for: The Effectiveness of Physical Adjunctive Interventions in the Acceleration of Orthodontic Tooth Movement: An Umbrella Review and Meta‐Analysis
Source: Int J Dent. 2026 Feb 3;2026:9131541. doi: 10.1155/ijod/9131541 (PMC12868923; doi:10.1155/ijod/9131541)
Supplement: Supplementary file 4 — Supporting Information 4 Table S4: Characteristics of the pooled primary studies on accelerating orthodontic treatment using VDs. [file IJOD-2026-9131541-s005.docx]

| **Supplementary Table 4:** Characteristics of the Pooled Primary Studies on Accelerating Orthodontic Treatment Using VDs | | | | | | | | | | | | | | |
| --- | --- | --- | --- | --- | --- | --- | --- | --- | --- | --- | --- | --- | --- | --- |
| **Treatment Method** | | **Vibration Device** | | **Orthodontic Device** | **Study** | **No. Patients** | **Vibration Parameters** | **Study Overlap Frequency** | **Bias Assessment Tools** | **Judgments** | | | | **Re-Assessment Using the RoB2 Tool** |
|  |  |  |  |  |  |  |  |  |  | **H** | **U** | **S** | **L** |  |
| Alignment & leveling  (13 RCTs)  (13 Parallel) | Mandible without extraction  (7 RCTs) | AcceleDent  (4 RCTs) | | FA | Miles & Fisher. 2016 (1) | 40 | 30 Hz/ 0.25N/ 20 min/ day | 7 | ROB1+2 | **4** | **1** | **1** | **1** | **S** |
|  |  |  |  |  | Chouinard 2016 (2) | 23 | 30 Hz/ 0.25N/ 20 min/ day | 1 | ROB 1 |  | **1** |  |  | **S** |
|  |  |  |  |  | Kalemaj et al. 2017 (3) | 33 | 30 Hz/ 0.25N/ 20 min/ day | 1 | ROB1+2 |  |  |  | **1** | **S** |
|  |  |  |  |  | Reiss et al. 2018 (4) | 40 | 30 Hz/ 0.25N/ 20 min/ day | 1 | ROB 1 | **1** |  |  |  | **S** |
|  |  | Tooth Masseuse (1 RCT) | |  | Miles et al. 2012 (5) | 64 | 111 Hz/ 0.06N/20min/day | 7 | ROB1+2 | **4** | **2** | **1** |  | **S** |
|  |  | Customized appliance (1 RCT) | |  | Bisht. 2019 (6) | 36 | 30 Hz/ 0.25N/ 20 min/ day | 1 | ROB1+2 |  |  | **1** |  | **S** |
|  |  | VPro5 (1 RCT) | | Aligner | Alansari. 2017 (7) | 60 | 120 Hz/ 0.0003N/ 5 min/ day | 3 | ROB1+2 | **1** | **1** | **1** |  | **H** |
|  | Mandible with extraction  (1 RCT) | AcceleDent (1 RT) | | FA | Woodhouse et al. 2015 (8) | 81 | 30 Hz/ 0.25N/ 20 min/ day | 7 | ROB1+2 | **1** | **3** | **1** | **2** | **L** |
|  | Both jaws without extraction  (5 RCTs) | AcceleDent  (5 RCTs) | | FA | Bulic. 2017 (9) | 71 | 30 Hz/ 0.25N/ 20 min/ day | 1 | ROB 1 |  | **1** |  |  | **S** |
|  |  |  |  | Aligner | Pescheret. 2017 (10) | 40 | 30 Hz/ 0.25N/ 20 min/ day | 1 | ROB 1 |  | **1** |  |  | **H** |
|  |  |  |  |  | Bragassa. 2018 (11) | 33 | 30 Hz/ 0.25N/ 20 min/ day | 2 | ROB 1 | **1** |  |  | **1** | **S** |
|  |  |  |  |  | Katchooi et al. 2018 (12) | 27 | 30 Hz/ 0.25N/ 20 min/ day | 6 | ROB1+2 |  | **3** |  | **3** | **L** |
|  |  |  |  |  | Lombardo et al. 2019 (13) | 45 | 30 Hz/ 0.25N/ 20 min/ day | 3 | ROB 1 | **1** | **2** |  |  | **L** |
| Canine Retraction  (11 RCTs)  (4 parallel/ 7 SMD) | | AcceleDent  ((4 RCTs | Parallel | FA | Pavlin et al. 2015 (14) | 45 | 30 Hz/ 0.25N/ 20 min/ day | 7 | ROB1+2 | **4** | **2** | **1** |  | **S** |
|  |  |  |  |  | Taha et al. 2019 (15) | 22 | 30 Hz/ 0.25N/ 20 min/ day | 4 | ROB 1 | **3** | **1** |  |  | **S** |
|  |  |  |  |  | Telatar et al. 2020 (16) | 20 | 30 Hz/ 0.25N/ 20 min/ day | 2 | ROB1+2 | **1** |  | **1** |  | **S** |
|  |  |  | SMD |  | Yildiz et al. 2023 (17) | 20 | 30 Hz/ 0.25N/ 20 min/ day | 1 | ROB1+2 |  |  | **1** |  | **S** |
|  |  | Tooth Bruch  (5 RCTs) | Parallel | FA | Siriphan et al. 2019 (18) | 60 | 30 – 60 – 120 Hz/ 0.001N/ 20 min/ day | 4 | ROB1+2 | **3** |  | **1** |  | **S** |
|  |  |  | SMD |  | Leethanakul et al. 2016 (19) | 15 | 125 Hz/ 20 min/ day | 3 | ROB1+2 | **1** |  | **1** | **1** | **L** |
|  |  |  |  |  | Liao et al. 2017 (20) | 16 | 50 Hz/ 0.2N/ 10 min/ day | 3 | ROB1+2 | **1** | **1** | **1** |  | **S** |
|  |  |  |  |  | Kannan et al. 2019 (21) | 23 | 100-103 Hz/ 5 min/ day | 2 | ROB1+2 |  | **1** | **1** |  | **S** |
|  |  |  |  |  | Azeem et al. 2019 (22) | 28 | 125 Hz/ 20 min/ day | 4 | ROB1+2 | **1** | **2** | **1** |  | **S** |
|  |  | Customized appliance  (2 RCTs) | SMD | FA | Mayama et al.2022 (23) | 25 | 102.2±2.6 Hz/ 0.05N/ 3 min/ Visit | 1 | ROB1+2 |  |  |  | **1** | **L** |
|  |  |  |  |  | Khera et al. 2022 (24) | 30 | 30 Hz/ 0.25N/ 20min/ day | 1 | ROB1+2 |  |  | **1** |  | **S** |
| En Masse Retraction  (4 RCTs)  (4 Parallel) | | AcceleDent  (2 RCTs) | Parallel | FA | Miles et al. 2018 (25) | 40 | 30 Hz/ 0.25N/ 20 min/ day | 4 | ROB1+2 | **2** |  | **1** | **1** | **S** |
|  |  |  |  |  | Dibiase et al. 2018 (26) | 81 | 30 Hz/ 0.25N/ 20 min/ day | 6 | ROB1+2 | **2** | **2** | **1** | **1** | **L** |
|  |  | Customized appliance  (2 RCTs) | Parallel |  | Kumar et al. 2020 (27) | 65 | 30 Hz/ 0.2N/ 20 min/ day | 3 | ROB1+2 | **1** |  | **1** | **1** | **S** |
|  |  |  |  |  | Gujar et al. 2023 (28) | 30 | 125-150 Hz/ 1 min/ tooth | 1 | ROB1+2 |  |  | **1** |  | **S** |
| **VDs**: Vibration Devices; **RCT**: Randomized Controlled Trial; **RCTs**: Randomized Controlled Trials; **SMD**: Split-Mouth Design; **FA**: Fixed Appliance; **RoB/ROB 1 & 2**: Risk-of-Bias assessment tool (versions 1 and 2); **H**: High risk of bias; **U:** Unclear risk of bias; **S**: Some concerns; **L**: Low risk of bias. | | | | | | | | | | | | | | |

1. Miles P, Fisher E; Assessment of the changes in arch perimeter and irregularity in the mandibular arch during initial alignment with the AcceleDent Aura appliance vs no appliance in adolescents: A single-blind randomized clinical trial. *Am J Orthod Dentofacial Orthop* 2016;**150**(6):928-936. doi: 10.1016/j.ajodo.2016.07.016.

2. Chouinard M-C; Biomarkers of Orthodontic Tooth Movement with Fixed Appliances and Vibration Device: A Randomized Clinical Trial. University of Connecticut, 2016.

3. Kalemaj Z, Buti J, Deregibus A, et al.; Aligning Effectiveness, Secretion of Interleukin 1β and Pain Control During Fixed Orthodontic Treatment with Self-Ligating Appliances and Supplemental Vibrational Appliances. A Randomized Controlled Clinical Trial. *Journal of Biomedicine* 2017;**2**:25-33. doi: 10.7150/jbm.17492.

4. Reiss S, Chouinard MC, Frias Landa D, et al.; Biomarkers of orthodontic tooth movement with fixed appliances and vibration appliance therapy: a pilot study. *Eur J Orthod* 2020;**42**(4):378-386. doi: 10.1093/ejo/cjaa026.

5. Miles P, Smith H, Weyant R, et al.; The effects of a vibrational appliance on tooth movement and patient discomfort: a prospective randomised clinical trial. *Aust Orthod J* 2012;**28**(2):213-8. doi: 10.2478/aoj-2012-0019.

6. Bisht D, Goyal V, Singh G, et al.; To Study the Effect of Vibrations on Pain and Rate of Anterior Teeth Alignment: An Invivo Study. *Journal of Contemporary Orthodontics* 2019;**3**(2). doi: 10.18231/j.jco.2019.011.

7. Alansari S, Atique M, Gomez J, et al.; The effects of brief daily vibration on clear aligner orthodontic treatment. *Journal of the World Federation of Orthodontists* 2018;**7**. doi: 10.1016/j.ejwf.2018.10.002.

8. Woodhouse NR, DiBiase AT, Johnson N, et al.; Supplemental vibrational force during orthodontic alignment: a randomized trial. *J Dent Res* 2015;**94**(5):682-9. doi: 10.1177/0022034515576195.

9. Bulic M; The Effect of AcceleDent on Arch Alignment and Pain Level During Orthodontic Treatment with SureSmile. University of Illinois Chicago, 2017.

10. Pescheret C; The Effect of AcceleDent on Arch Alignment and Pain Level During Orthodontic Treatment with Invisalign. University of Illinois Chicago, 2017.

11. Bragassa B; Accelerated Invisalign® In Conjunction with Acceledent Aura®: A Randomized Clinical Trial. *Department of Orthodontics*: University of North Carolin, 2018.

12. Katchooi M, Cohanim B, Tai S, et al.; Effect of supplemental vibration on orthodontic treatment with aligners: A randomized trial. *Am J Orthod Dentofacial Orthop* 2018;**153**(3):336-346. doi: 10.1016/j.ajodo.2017.10.017.

13. Lombardo L, Arreghini A, Huanca Ghislanzoni LT, et al.; Does low-frequency vibration have an effect on aligner treatment? A single-centre, randomized controlled trial. *Eur J Orthod* 2019;**41**(4):434-443. doi: 10.1093/ejo/cjy076.

14. Pavlin D, Anthony R, Raj V, et al.; Cyclic Loading (Vibration) Accelerates Tooth Movement in Orthodontic Patients: A Double-Blind, Randomized Controlled Trial. *Seminars in Orthodontics* 2015;**26**. doi: 10.1053/j.sodo.2015.06.005.

15. Taha K, Conley RS, Arany P, et al.; Effects of mechanical vibrations on maxillary canine retraction and perceived pain: a pilot, single-center, randomized-controlled clinical trial. *Odontology* 2020;**108**(2):321-330. doi: 10.1007/s10266-019-00480-0.

16. Telatar BC, Gungor AY; Effectiveness of vibrational forces on orthodontic treatment : A randomized, controlled clinical trial. *J Orofac Orthop* 2021;**82**(5):288-294. doi: 10.1007/s00056-020-00257-z.

17. Yildiz O, Yagci A, Hashimli N; A different method to accelerate orthodontic tooth movement: Randomized controlled trial. *Balkan Journal of Dental Medicine* 2023;**27**:51-55. doi: 10.5937/bjdm2301051Y.

18. Siriphan N, Leethanakul C, Thongudomporn U; Effects of two frequencies of vibration on the maxillary canine distalization rate and RANKL and OPG secretion: A randomized controlled trial. *Orthod Craniofac Res* 2019;**22**(2):131-138. doi: 10.1111/ocr.12301.

19. Leethanakul C, Suamphan S, Jitpukdeebodintra S, et al.; Vibratory stimulation increases interleukin-1 beta secretion during orthodontic tooth movement. *Angle Orthod* 2016;**86**(1):74-80. doi: 10.2319/111914-830.1.

20. Liao Z, Elekdag-Turk S, Turk T, et al.; Computational and clinical investigation on the role of mechanical vibration on orthodontic tooth movement. *J Biomech* 2017;**60**:57-64. doi: 10.1016/j.jbiomech.2017.06.012.

21. Kannan S, Fassul S, Singh AK, et al.; Effectiveness and importance of powered tooth brushes in tooth movement. *J Family Med Prim Care* 2019;**8**(7):2478-2483. doi: 10.4103/jfmpc.jfmpc_352_19.

22. Azeem M, Afzal A, Jawa SA, et al.; Effectiveness of electric toothbrush as vibration method on orthodontic tooth movement: a split-mouth study. *Dental Press J Orthod* 2019;**24**(2):49-55. doi: 10.1590/2177-6709.24.2.049-055.oar.

23. Mayama A, Seiryu M, Takano-Yamamoto T; Effect of vibration on orthodontic tooth movement in a double blind prospective randomized controlled trial. *Sci Rep* 2022;**12**(1):1288. doi: 10.1038/s41598-022-05395-5.

24. Khera AK, Raghav P, Mehra V, et al.; Effect of customized vibratory device on orthodontic tooth movement: A prospective randomized control trial. *J Orthod Sci* 2022;**11**:18. doi: 10.4103/jos.jos_127_21.

25. Miles P, Fisher E, Pandis N; Assessment of the rate of premolar extraction space closure in the maxillary arch with the AcceleDent Aura appliance vs no appliance in adolescents: A single-blind randomized clinical trial. *Am J Orthod Dentofacial Orthop* 2018;**153**(1):8-14. doi: 10.1016/j.ajodo.2017.08.007.

26. DiBiase AT, Woodhouse NR, Papageorgiou SN, et al.; Effects of supplemental vibrational force on space closure, treatment duration, and occlusal outcome: A multicenter randomized clinical trial. *American Journal of Orthodontics and Dentofacial Orthopedics* 2018;**153**(4):469-480.e4. doi: <https://doi.org/10.1016/j.ajodo.2017.10.021>.

27. Kumar V, Batra P, Sharma K, et al.; Comparative assessment of the rate of orthodontic tooth movement in adolescent patients undergoing treatment by first bicuspid extraction and en mass retraction, associated with low-frequency mechanical vibrations in passive self-ligating and conventional brackets: A randomized controlled trial. *Int Orthod* 2020;**18**(4):696-705. doi: 10.1016/j.ortho.2020.08.003.

28. Gujar AN, Shivamurthy PG; Effect of 125 Hz and 150 Hz vibrational frequency electric toothbrushes on the rate of orthodontic tooth movement and prostaglandin E2 levels. *Korean J Orthod* 2023;**53**(5):307-316. doi: 10.4041/kjod23.076.
