# Supplementary material for: The Effectiveness of Physical Adjunctive Interventions in the Acceleration of Orthodontic Tooth Movement: An Umbrella Review and Meta‐Analysis
Source: Int J Dent. 2026 Feb 3;2026:9131541. doi: 10.1155/ijod/9131541 (PMC12868923; doi:10.1155/ijod/9131541)
Supplement: Supplementary file 5 — Supporting Information 5 Table S5: Characteristics of the pooled primary studies on accelerating orthodontic treatment using PBM. [file IJOD-2026-9131541-s004.docx]

| **Supplementary Table 5:** Characteristics of the Pooled Primary Studies on Accelerating Orthodontic Treatment Using **PBM** | | | | | | | | | | | | | | | | | | |
| --- | --- | --- | --- | --- | --- | --- | --- | --- | --- | --- | --- | --- | --- | --- | --- | --- | --- | --- |
| **Treatment Method** | | **Vibration Device** | **Orthodontic Device** | **Study (Year)** | **No. Patients** | **WL (nm)** | **Output power** | **Total energy density** | **Irradiation points** | **Time** | **Sessions** | **Study Overlap Frequency** | **Bias Assessment Tools** | **Judgments** | | | | **Re-Assessment Using the RoB2 Tool** |
|  |  |  |  |  |  |  |  |  |  |  |  |  |  | **H** | **U** | **S** | **L** |  |
| Alignment & leveling  (9 RCTs)  (9 Parallel) | Mandible without extraction  (5 RCTs) | LLLT | FA | Caccianiga et al. 2017 (1) | 36 | 980 | 1W | 150 J/cm² | Intraoral (1.5 cm from arch) | 150s (3 sessions) | Monthly until alignment | 6 | ROB 1+2 | 2 | 2 |  | 2 | **S** |
|  |  |  |  | El Shehawy et al. 2020 (2) | 26 | 635 | 20 mW | 6.5 J/cm² | Intraoral (10 points: 5 facial & 5 lingual around mandibular anterior teeth roots) | 10 sec per session | 12 sessions (4/month × 3 months) | 1 | ROB 1 | 1 |  |  |  | **S** |
|  |  |  |  | Ghaffar et al. 2022 (3) | 30 | 940 ± 10 | 2.5 W | 25.7 J/cm² | Labial vestibule | 30 sec/zone | Days 3, 7, 14; monthly → biweekly | 2 | ROB 1+2 | 1 |  |  | 1 | **S** |
|  |  | LED |  | Nahas et al. 2017 (4) | 34 | 850 | 90 mW/cm² | 108 J/cm² | Extraoral (on cheek) | 20 min/day | Daily (~68 sessions) | 5 | ROB 1+2 | 1 | 2 | 1 | 1 | **H** |
|  |  |  |  | Lo Giudice et al. 2022 (5) | 89 | 470, 525, 590, 620, 680–760, 800–835 | 144 J/cm² (per session) | 144 J/cm² | Extraoral via 3 fixed panels | 18 minutes | Every 14 days (median: 7 sessions) | 2 | ROB 1+2 | 1 |  |  | 1 | **S** |
|  | Maxilla with extraction  (1 RCTs) | LLLT | FA | Alsayed Hasan et al. 2017 (6) | 26 | 830 | 150 mW | 2.25 J/cm² | Direct mucosal contact | 60 seconds/tooth | 4 sessions/month → biweekly | 7 | ROB 1+2 | 5 | 1 |  | 1 | **S** |
|  | Maxilla without extraction  (1 RCTs) | LED | FA | Okla et al. 2018 (7) | 38 | 850 | 0.065 J/cm² | 0.065 J/cm² | Intraoral (post-braces) | 5 min/arch/day | Daily (~41 days) | 2 | ROB 1 | 1 |  |  | 1 | **H** |
|  | Both jaws without extraction  (2 RCTs) | LED | FA | Kau et al. 2013 (8) | 90 | 850 | 60 mW/cm² | 72–216 J/cm² | Extraoral (cheek device) | 20–60 min/session | Daily/weekly until alignment | 1 | ROB 1 |  | 1 |  |  | **S** |
|  |  | LLLT | AA | Caccianiga et al. 2016 (9) | 21 | 980 | 1 W | 150 J/cm² | Extraoral (zygomatic/nasal) | 150 seconds/arch | Biweekly (~40 weeks) | 1 | ROB 1 |  | 1 |  |  | **H** |
| Canine Retraction  (28 RCTs)  (28 SMD) | Upper Canine Retraction  (22 RCTs)  (22 SMD) | LED | FA | Ekizer et al. 2016 (10) | 20 | 618 | 20 mW/cm² | 24 J/cm² | Transcutaneous (over cheek surface above canine and miniscrew areas) | 20 minutes | 21 sessions (daily) | 2 | ROB 1 |  |  |  | 2 | **S** |
|  |  |  |  | Farhadian et al. 2021 (11) | 56 | 640 | 40 mW/cm² | 10 J/cm² | Intraoral (Buccal surfaces) | 5 minutes/day | Daily until alignment completion | 1 | ROB 1 | 1 |  |  |  | **S** |
|  |  |  |  | Al-Shafi et al. 2021 (12) | 20 | 850 | 60 mW/cm² | 18 J/cm²* | Intraoral (OrthoPulse® device) | 5 minutes/day | Daily for 12 weeks (84 sessions) | 1 | ROB 2 |  |  |  | 1 | **S** |
|  |  | LLLT |  | Cruz et al. 2004 (13) | 11 | 780 | 20 mW | 16 J | 10 points (direct contact with mucosa, vertical application) | 100 seconds | 8 sessions (4 days/month for 2 months) | 6 | SCS+ ROB 1 | 1 | 4 | 1 |  | **S** |
|  |  |  |  | Limpanichkul et al. 2006 (14) | 12 | 860 | 100 mW | 166.32 J | 8 points (3 buccal, 2 lateral, 3 palatal) | 184 seconds | 9 sessions (3 consecutive days/month × 3 months) | 5 | ROB 1+2 |  | 3 | 1 | 1 | **S** |
|  |  |  |  | Youssef et al. 2008 (15) | 15 | 809 | 100 mW | 32 J | 6 points (3 buccal, 3 palatal; cervical/apical and middle thirds) | 80 seconds | 4 sessions per reactivation cycle | 1 | ROB 1 | 1 |  |  |  | **S** |
|  |  |  |  | Sousa et al. 2011 (16) | 10 | 780 | 20 mW | 18 J | 10 points (vertical contact with isolated mucosa) | 100 seconds | 9 sessions (3 days/month × 3 months) | 6 | SCS+ ROB 1+2 | 2 | 3 | 1 |  | **S** |
|  |  |  |  | Hosseini 2011 (17) | 12 | 890 | 80 W (peak) | 72 J | 2 points (buccal and palatal mucosa; slow probe movement covering the entire root length) | 72 seconds | 6 sessions (every 48 hours over 2 weeks) | 1 | ROB 1 |  |  |  | 1 | **L** |
|  |  |  |  | Kansal et al. 2014 (18) | 10 | 904 (IR) | 12 mW | 12 J | 10 points (5 buccal, 5 palatal mucosa) | 100 seconds | 10 sessions (days 1, 3, 7, 14, 21, 28, 35, 42, 49, 56) | 5 | SCS+ ROB 1+2 | 2 | 1 | 1 | 1 | **S** |
|  |  |  |  | Heravi 2014 (19) | 20 | 810 | 200 mW | 660 J | 10 points (perpendicular application to gingival mucosa with direct contact) | 300 seconds | 11 sessions (days 0, 3, 7, 11, 15, 28, 32, 35, 39, 43, 56) | 3 | ROB 1 | 2 | 1 |  |  | **S** |
|  |  |  |  | Yassaei et al. 2016 (20) | 11 | 980 | 100 mW | 308 J | 6 points (3 buccal, 3 lingual; cervical/middle third: 10 sec/point, apical: 8 sec/point) | 56 seconds/tooth | 5 sessions (days 0, 7, 14, 21, 28) | 3 | ROB 1+2 |  | 2 | 1 |  | **L** |
|  |  |  |  | Üretürk et al. 2017 (21) | 15 | 820 | 20 mW | 34 J | 10 points (5 buccal, 5 palatal with cotton isolation) | 100 seconds | 17 sessions (days 0, 3, 7, 14, 21, 30, 33, 37, 44, 51, 60, 63, 67, 74, 81, 84, 90) | 6 | ROB 1+2 |  | 4 | 1 | 1 | **S** |
|  |  |  |  | Qamruddin et al. 2017 (22) | 20 | 940 | 100 mW | 9 J | 10 points (3 seconds per point) | 30 seconds | 3 sessions (Weeks 0, 3, 6) | 6 | ROB 1+2 | 2 | 2 | 1 | 1 | **L** |
|  |  |  |  | Varella et al. 2018 (23) | 10 | 940 | 100 mW | 90 J | 10 points (buccal/lingual areas) | 100 seconds | 9 sessions (3 consecutive days at weeks 0, 4, 8) | 3 | ROB 1+2 |  | 1 | 1 | 1 | **S** |
|  |  |  |  | Mal et al. 2018 (24) | 10 | 940 | 0.2 mW | 350 J | 10 points (5 buccal, 5 palatal; cervical, apical, and middle thirds of the canine) | 250 seconds | ~12 sessions (days 0, 3, 7, 14 + every 15 days for 4 months) | 1 | ROB 1 |  | 1 |  |  | **S** |
|  |  |  |  | Isola et al. 2019 (25) | 41 | 810 | 1 W | 66.7 J/cm² | Buccal and palatal sides (3 points/side: distal, medial, mesial) | 15 seconds/area | Baseline + days 3, 7, 14 + every 15 days until space closure (~6-7 sessions) | 1 | ROB 2 |  |  |  | 1 | **S** |
|  |  |  |  | Alam. 2019 (26) | 32 | 940 | 100 mW | 75 J/tooth | 5 points/tooth | 3 seconds | 1 | 1 | ROB 1 | 1 |  |  |  | **H** |
|  |  |  |  | Impellizzeri et al. 2020 (27) | 6 | 650 (Red) / 910 (IR) | 100–500 mW | 0.24 J | 6 points (3 buccal, 3 palatal; non-pressure contact) | 60 seconds | 4 sessions (Days 0, 3, 7, 14) | 1 | ROB 2 |  |  | 1 |  | **S** |
|  |  |  |  | Mistry et al. 2020 (28) | 21 | 808 ± 5 | 200 mW | 41.28 J | 8 points (4 buccal, 4 palatal) | 80 seconds | 3 sessions (Days 0, 28, 56) | 2 | ROB 1+2 |  |  |  | 2 | **L** |
|  |  |  |  | Zheng et al. 2021 (29) | 12 | 810 | 100 mW | 64 J | 4 points (direct mucosal application) | 160 seconds | 4 sessions (Days 0, 7, 14, 21) | 2 | ROB 2 |  |  | 1 | 1 | **S** |
|  |  |  |  | Pérignon et al. 2021 (30) | 42 | 970 | 0.5 W | 30 J/cm² | 24 (6 points per tooth on 4 teeth) | 2 seconds | 2 (M0 & M1) | 1 | ROB 2 |  |  |  | 1 | **S** |
|  |  |  |  | Kharat et al. 2023 (31) | 20 | 940 | 0.1 W (100 mW) | Not specified | 30 (3 points/side × 5 buccal + 5 palatal) | 30 seconds (Analgesic), 10 seconds (Biosimulation) | Initial month: Days 0, 3, 7, 14.  Subsequent months: Every 15 days until retraction completion.  ~10 | 1 | ROB 2 |  | 1 |  |  | **S** |
|  | Upper and Lower Canine Retraction  (6 RCTs)  (6 SMD) | LLLT | FA | Doshi-Mehta et al. 2012 (32) | 20 | 808 ± 10 | 0.25–0.7 mW | 8 J/application | 10 points (5 buccal, 5 lingual sites) | 100 seconds | ~11 sessions (Days 0, 3, 7, 14 + every 15 days for ~4.5 months) | 7 | SCS+ ROB 1+2 | 3 | 3 | 1 |  | **S** |
|  |  |  |  | Souza. 2014 (33) | 11 | 780 | 40–70 mW | 39 J | Upper Jaw: 10 points (5 buccal, 5 palatal)  Lower Jaw: 10 points | Upper: 150 seconds  Lower: 100 seconds | 3 sessions (monthly for 3 months) | 3 | SCS+ ROB 1+2 | 1 | 1 | 1 |  | **S** |
|  |  |  |  | Pereira et al. 2014 (34) | 11 | 780 | 40–70 mW | 39 J | Upper Jaw: 10 points (5 buccal, 5 palatal)  Lower Jaw: 10 points (5 buccal, 5 lingual) | Upper: 150 seconds  Lower: 100 seconds | 3 sessions (monthly for 3 months) | 3 | SCS+ ROB 1+2 | 1 | 1 | 1 |  | **S** |
|  |  |  |  | Dalaie et al. 2015 (35) | 12 | 880 | 100 mW | 5 J/cm²  Total: 64–72J | 8 points (buccal/lingual, non-contact) | 80 seconds | 8–9 sessions (monthly) | 4 | ROB 1+2 | 1 | 2 | 1 |  | **S** |
|  |  |  |  | Kochar et al. 2017 (36) | 20 | 810 | 100 mW | 5 J/cm² | 10 points (vertical probe on tissue) | 100 seconds | Initial: days 0, 3, 7; repeated every 21 days | 4 | ROB 1+2 | 1 | 1 | 1 | 1 | **S** |
|  |  |  |  | Guram et al. 2018 (37) | 20 | 810 | 200 mW | 18 J | 6 points (3 buccal, 3 lingual near the root) | 30 seconds | 9 sessions (3 sessions/week for 21 days) | 4 | ROB 1+2 |  | 2 | 2 |  | **S** |
| En-masse Retraction  (4 RCTs)  (2 parallel/ 2 SMD) | Upper En masse Retraction  (2 RTCTs)  (2 SMD) | LLLT | FA | Sandoval et al. 2017 (38) | 20 | 940 | 100 mW | 576 J | 12 points (buccal and palatal, non-contact) | 720 seconds | 3 initial sessions + every 15 days (avg. 6–7 sessions) | 1 | ROB 1 |  | 1 |  |  | **S** |
|  |  |  |  | Arumughan et al. 2018 (39) | 12 | 810 | 100 mW | 40 J | 10 points (5 vestibular, 5 palatal) | 100 seconds | 4 sessions (Days 1, 21, 42, 63) | 4 | ROB 1+2 | 1 | 1 | 2 |  | **S** |
|  | Upper and Lower En masse Retraction  (2 RCTs)  (2 Parallel) | LLLT | FA | Lalnunpuii et al. 2020 (40) | 65 | 658 | 8 mW | 88 J | 10 points (direct tissue contact) | 100 seconds | Variable (Days 0, 3, 7, 14; then every 15 days) | 2 | ROB 1+2 | 1 |  |  | 1 | **S** |
|  |  | LED |  | Samara et al. 2018 (41) | 60 | 850 | 33 mW/cm²/arch/day | 6 J/cm² | Intraoral via OrthoPulse™ device | 180 sec (3 min) | 130 sessions (daily for ≈130 days) | 1 | ROB 1 | 1 |  |  |  | **S** |
| Upper premolar Retraction  (1 RCT)  (SMD) | | LLLT | FA | Domínguez et al. 2015 (42) | 10 | 670 | 200 mW | 648 J | 3 points (distal, buccal, lingual periodontal pockets) | 540 seconds (9 min) | 6 sessions (Days 0, 1, 2, 3, 4, 7) | 1 | ROB 1 | 1 |  |  |  | **H** |
| Upper first molar intrusion  (2 RCT)  parallel | | LLLT | FA | Abellán et al. 2021 (43) | 20 | 670 | 150 mW | 11.3 J/cm² | Gingival surfaces (distal, mesial, vestibular, palatal) | 12 minutes/session (3 min per surface) | Days 0, 1, 2, 3, 4, 7 + monthly follow-up for 6 months | 1 | ROB 1 | 1 |  |  |  | **H** |
|  |  |  |  | Hasan et al. 2022 (44) | 42 | 808 | 250 mW | 4 J/point (calculated) | Direct contact on buccal and palatal mucosa (6 points) | 16 seconds/point | ~14 sessions (every 15 days for 7 months) | 1 | ROB 1 | 1 |  |  |  | **L** |
| **PBM**: Photobiomodulation; **LLLT**: Low-Level Laser Therapy; **LED**: Light-Emitting Diode; **RCT**: Randomized Controlled Trial; **SMD**: Split-Mouth Design; **FA**: Fixed Appliance; **AA**: Aligner Appliance; **WL**: Wavelength; **IR**: Infrared; **RoB**/**ROB** **1** **&** **2**: Risk-of-Bias assessment tool (versions 1 and 2); **SCS**: Study Characteristics Score; **H**: High risk of bias; **U**: Unclear risk of bias; **S**: Some concerns; **L**: Low risk of bias; **M0**: Month 0 (baseline); **M1**: Month 1. | | | | | | | | | | | | | | | | | | |

1. Caccianiga G, Paiusco A, Perillo L, et al.; Does Low-Level Laser Therapy Enhance the Efficiency of Orthodontic Dental Alignment? Results from a Randomized Pilot Study. *Photomed Laser Surg* 2017;**35**(8):421-426. doi: 10.1089/pho.2016.4215.

2. El Shehawy TO, Hussein FA, Ei Awady AA; Outcome of photodynamic therapy on orthodontic leveling and alignment of mandibular anterior segment: A controlled clinical trial. *Photodiagnosis Photodyn Ther* 2020;**31**:101903. doi: 10.1016/j.pdpdt.2020.101903.

3. Ghaffar YKA, El Sharaby FA, Negm IM; Effect of low-level laser therapy on the time needed for leveling and alignment of mandibular anterior crowding. *Angle Orthod* 2022;**92**(4):478-486. doi: 10.2319/102721-795.1.

4. Nahas AZ, Samara SA, Rastegar-Lari TA; Decrowding of lower anterior segment with and without photobiomodulation: a single center, randomized clinical trial. *Lasers Med Sci* 2017;**32**(1):129-135. doi: 10.1007/s10103-016-2094-5.

5. Lo Giudice A, Nucera R, Leonardi R, et al.; A Comparative Assessment of the Efficiency of Orthodontic Treatment With and Without Photobiomodulation During Mandibular Decrowding in Young Subjects: A Single-Center, Single-Blind Randomized Controlled Trial. *Photobiomodul Photomed Laser Surg* 2020;**38**(5):272-279. doi: 10.1089/photob.2019.4747.

6. AlSayed Hasan MMA, Sultan K, Hamadah O; Low-level laser therapy effectiveness in accelerating orthodontic tooth movement: A randomized controlled clinical trial. *Angle Orthod* 2017;**87**(4):499-504. doi: 10.2319/062716-503.1.

7. Okla N, Bader DA, Makki L; Effect of Photobiomodulation on Maxillary Decrowding and Root Resorption: A Randomized Clinical Trial. *APOS Trends in Orthodontics* 2018;**8**:86. doi: 10.4103/apos.apos_46_18.

8. Kau CH, Kantarci A, Shaughnessy T, et al.; Photobiomodulation accelerates orthodontic alignment in the early phase of treatment. *Prog Orthod* 2013;**14**:30. doi: 10.1186/2196-1042-14-30.

9. Caccianiga G, Crestale C, Cozzani M, et al.; Low-level laser therapy and invisible removal aligners. *J Biol Regul Homeost Agents* 2016;**30**(2 Suppl 1):107-13.

10. Ekizer A, Türker G, Uysal T, et al.; Light emitting diode mediated photobiomodulation therapy improves orthodontic tooth movement and miniscrew stability: A randomized controlled clinical trial. *Lasers Surg Med* 2016;**48**(10):936-943. doi: 10.1002/lsm.22516.

11. Farhadian N, Miresmaeili A, Borjali M, et al.; The effect of intra-oral LED device and low-level laser therapy on orthodontic tooth movement in young adults: A randomized controlled trial. *Int Orthod* 2021;**19**(4):612-621. doi: 10.1016/j.ortho.2021.09.002.

12. Al-Shafi S, Pandis N, Darendeliler MA, et al.; Effect of light-emitting diode-mediated photobiomodulation on extraction space closure in adolescents and young adults: A split-mouth, randomized controlled trial. *Am J Orthod Dentofacial Orthop* 2021;**160**(1):19-28. doi: 10.1016/j.ajodo.2020.12.021.

13. Cruz DR, Kohara EK, Ribeiro MS, et al.; Effects of low-intensity laser therapy on the orthodontic movement velocity of human teeth: a preliminary study. *Lasers Surg Med* 2004;**35**(2):117-20. doi: 10.1002/lsm.20076.

14. Limpanichkul W, Godfrey K, Srisuk N, et al.; Effects of low-level laser therapy on the rate of orthodontic tooth movement. *Orthod Craniofac Res* 2006;**9**(1):38-43. doi: 10.1111/j.1601-6343.2006.00338.x.

15. Youssef M, Ashkar S, Hamade E, et al.; The effect of low-level laser therapy during orthodontic movement: a preliminary study. *Lasers Med Sci* 2008;**23**(1):27-33. doi: 10.1007/s10103-007-0449-7.

16. Sousa MV, Scanavini MA, Sannomiya EK, et al.; Influence of low-level laser on the speed of orthodontic movement. *Photomed Laser Surg* 2011;**29**(3):191-6. doi: 10.1089/pho.2009.2652.

17. Hosseini M, Mahmoodzadeh Darbandi M, Kamali A; Effect of low level therapy on orthodontic movement in human %J Journal of Dental Medicine. *J Dent Med* 2011;**24**(3):156-164.

18. Kansal A, Kittur N, Kumbhojkar V, et al.; Effects of low-intensity laser therapy on the rate of orthodontic tooth movement: A clinical trial. *Dent Res J (Isfahan)* 2014;**11**(4):481-8.

19. Heravi F, Moradi A, Ahrari F; The effect of low level laser therapy on the rate of tooth movement and pain perception during canine retraction. *Oral Health Dent Manag* 2014;**13**(2):183-8.

20. Yassaei S, Aghili H, Afshari JT, et al.; Effects of diode laser (980 nm) on orthodontic tooth movement and interleukin 6 levels in gingival crevicular fluid in female subjects. *Lasers Med Sci* 2016;**31**(9):1751-1759. doi: 10.1007/s10103-016-2045-1.

21. Üretürk SE, Saraç M, Fıratlı S, et al.; The effect of low-level laser therapy on tooth movement during canine distalization. *Lasers Med Sci* 2017;**32**(4):757-764. doi: 10.1007/s10103-017-2159-0.

22. Qamruddin I, Alam MK, Mahroof V, et al.; Effects of low-level laser irradiation on the rate of orthodontic tooth movement and associated pain with self-ligating brackets. *Am J Orthod Dentofacial Orthop* 2017;**152**(5):622-630. doi: 10.1016/j.ajodo.2017.03.023.

23. Varella AM, Revankar AV, Patil AK; Low-level laser therapy increases interleukin-1β in gingival crevicular fluid and enhances the rate of orthodontic tooth movement. *Am J Orthod Dentofacial Orthop* 2018;**154**(4):535-544.e5. doi: 10.1016/j.ajodo.2018.01.012.

24. Mal UH, Malagan M; Evaluation of the effects of laser irradiation on the rate of tooth movement: A split-mouth study. *Indian Journal of Health Sciences and Biomedical Research (KLEU)* 2018;**11**(5):42-50. doi: 10.4103/kleuhsj.kleuhsj_249_17.

25. Isola G, Matarese M, Briguglio F, et al.; Effectiveness of Low-Level Laser Therapy during Tooth Movement: A Randomized Clinical Trial. *Materials (Basel)* 2019;**12**(13). doi: 10.3390/ma12132187.

26. Alam MK; Laser-Assisted Orthodontic Tooth Movement in Saudi Population: A Prospective Clinical Intervention of Low-Level Laser Therapy in the 1st Week of Pain Perception in Four Treatment Modalities. *Pain Res Manag* 2019;**2019**:6271835. doi: 10.1155/2019/6271835.

27. Impellizzeri A, Horodynski M, Fusco R, et al.; Photobiomodulation Therapy on Orthodontic Movement: Analysis of Preliminary Studies with a New Protocol. *Int J Environ Res Public Health* 2020;**17**(10). doi: 10.3390/ijerph17103547.

28. Mistry D, Dalci O, Papageorgiou SN, et al.; The effects of a clinically feasible application of low-level laser therapy on the rate of orthodontic tooth movement: A triple-blind, split-mouth, randomized controlled trial. *Am J Orthod Dentofacial Orthop* 2020;**157**(4):444-453. doi: 10.1016/j.ajodo.2019.12.005.

29. Zheng J, Yang K; Clinical research: low-level laser therapy in accelerating orthodontic tooth movement. *BMC Oral Health* 2021;**21**(1):324. doi: 10.1186/s12903-021-01684-z.

30. Pérignon B, Bandiaky ON, Fromont-Colson C, et al.; Effect of 970 nm low-level laser therapy on orthodontic tooth movement during Class II intermaxillary elastics treatment: a RCT. *Sci Rep* 2021;**11**(1):23226. doi: 10.1038/s41598-021-02610-7.

31. Kharat DS, Pulluri SK, Parmar R, et al.; Accelerated Canine Retraction by Using Mini Implant With Low-Intensity Laser Therapy. *Cureus* 2023;**15**(1):e33960. doi: 10.7759/cureus.33960.

32. Doshi-Mehta G, Bhad-Patil WA; Efficacy of low-intensity laser therapy in reducing treatment time and orthodontic pain: a clinical investigation. *Am J Orthod Dentofacial Orthop* 2012;**141**(3):289-297. doi: 10.1016/j.ajodo.2011.09.009.

33. de SJ; Avaliação da influência do laser de baixa intensi dade na movimentação ortodôntica e supressão da dor. Universidade de São Paulo, Brazil, 2014.

34. C PSd; Influência do laser de baixa intensidade na movimentação ortodôntica-avaliação clínica e radiográfica. Universidade de São Paulo, Brazil, 2014.

35. Dalaie K, Hamedi R, Kharazifard MJ, et al.; Effect of Low-Level Laser Therapy on Orthodontic Tooth Movement: A Clinical Investigation. *J Dent (Tehran)* 2015;**12**(4):249-56.

36. Kochar GD, Londhe S, Varghese B, et al.; Effect of Low-level Laser Therapy on Orthodontic Tooth Movement. *Journal of Indian Orthodontic Society* 2017;**51**:81-86. doi: 10.1177/0974909820170204.

37. Guram G, Reddy RK, Dharamsi AM, et al.; Evaluation of Low-Level Laser Therapy on Orthodontic Tooth Movement: A Randomized Control Study. *Contemp Clin Dent* 2018;**9**(1):105-109. doi: 10.4103/ccd.ccd_864_17.

38. Sandoval P, Bizcar B, Navarro P, et al.; Efficacy of Diode Laser Therapy in Acceleration of Orthodontic Space Closure: A Split-Mouth Randomized Clinical Trial. *International Journal of Dentistry and Oral Health* 2017;**3**(2). doi: 10.16966/2378-7090.229.

39. Arumughan S, Somaiah S, Muddaiah S, et al.; A Comparison of the Rate of Retraction with Low-level Laser Therapy and Conventional Retraction Technique. *Contemp Clin Dent* 2018;**9**(2):260-266. doi: 10.4103/ccd.ccd_857_17.

40. Lalnunpuii H, Batra P, Sharma K, et al.; Comparison of rate of orthodontic tooth movement in adolescent patients undergoing treatment by first bicuspid extraction and en-mass retraction, associated with low level laser therapy in passive self-ligating and conventional brackets: A randomized controlled trial. *Int Orthod* 2020;**18**(3):412-423. doi: 10.1016/j.ortho.2020.05.008.

41. Samara S, Nahas A, Rastegar T; Velocity of orthodontic active space closure with and without photobiomodulation therapy: a single-center, cluster randomized clinical trial. *Lasers in Dental Science* 2018;**2**. doi: 10.1007/s41547-018-0026-3.

42. Domínguez A, Gómez C, Palma JC; Effects of low-level laser therapy on orthodontics: rate of tooth movement, pain, and release of RANKL and OPG in GCF. *Lasers Med Sci* 2015;**30**(2):915-23. doi: 10.1007/s10103-013-1508-x.

43. Abellán R, Gómez C, Palma JC; Effects of Photobiomodulation on the Upper First Molar Intrusion Movement Using Mini-Screws Anchorage: A Randomized Controlled Trial. *Photobiomodul Photomed Laser Surg* 2021;**39**(8):518-527. doi: 10.1089/photob.2020.4979.

44. Hasan AA, Rajeh N, Hajeer MY, et al.; Evaluation of the acceleration, skeletal and dentoalveolar effects of low-level laser therapy combined with fixed posterior bite blocks in children with skeletal anterior open bite: A three-arm randomised controlled trial. *Int Orthod* 2022;**20**(1):100597. doi: 10.1016/j.ortho.2021.10.005.
