# Supplementary material for: The Effectiveness of Physical Adjunctive Interventions in the Acceleration of Orthodontic Tooth Movement: An Umbrella Review and Meta‐Analysis
Source: Int J Dent. 2026 Feb 3;2026:9131541. doi: 10.1155/ijod/9131541 (PMC12868923; doi:10.1155/ijod/9131541)
Supplement: Supplementary file 6 — Supporting Information 6 Table S6: Characteristics of the pooled primary studies on accelerating orthodontic treatment using BES. [file IJOD-2026-9131541-s003.docx]

| **Supplementary Table 6:** Characteristics of the Pooled Primary Studies on Accelerating Orthodontic Treatment Using **BES** | | | | | | | | | | | | | |
| --- | --- | --- | --- | --- | --- | --- | --- | --- | --- | --- | --- | --- | --- |
| **Treatment Method** | | **Vibration Device** | **Orthodontic Device** | **Study** | **No. Patients** | **BES Parameters** | **Study Overlap Frequency** | **Bias Assessment Tools** | **Judgments** | | | | **Re-Assessment Using the RoB2 Tool** |
|  |  |  |  |  |  |  |  |  | **H** | **U** | **S** | **L** |  |
| Alignment & leveling  (1 RCT)  (1 Parallel) | Mandible without extraction | BM (1 RCT) | FA | Barsi et al. 2023 (1) | 28 | 10 μA/ 5 minutes/week  Lower incisors | 1 | ROB 2 |  |  |  | **1** | **S** |
| Canine Retraction  (3 RCTs)  (3 SMD) | | LIDEC  (1 RCT) | FA | Kim et al. 2008 (2) | 7 | 20 μA (15 V)/ 5 hours/day  Upper canines | 1 | ROB 2 | **1** |  |  |  | **H** |
|  |  | PEMF  (2 RCTs) |  | Showkatbakhsh et al. 2010 (3) | 10 | 0.5 mT, 1 Hz/ 8 hours/night  Upper canines | 1 | ROB 2 | **1** |  |  |  | **H** |
|  |  |  |  | Bhad et al. 2020 (4) | 19 |  | 1 | ROB 2 | **1** |  |  |  | **S** |
| **BES: Bioelectrical stimulation; BM: Bioelectrical microcurrent; RCT: Randomized controlled trial; SMD: Split-mouth design; FA: Fixed appliance; LIDEC: Low-intensity direct electrical current; PEMF: Pulsed electromagnetic field; ROB 2 (RoB2): Cochrane risk-of-bias assessment tool (version 2); H: High risk of bias; U: Unclear risk of bias; S: Some concerns; L: Low risk of bias.** | | | | | | | | | | | | | |

1. Barsi P, Casarin R, Stolf C, et al.; Can bioelectrical stimulation favor orthodontic treatment? A randomized clinical trial to evaluate tooth movement, patient-centered, and inflammatory biomarker outcomes. *AJO-DO Clinical Companion* 2023;**3**. doi: 10.1016/j.xaor.2023.09.004.

2. Kim J, Park Y, Kang S-G; The effects of electrical current from a micro-electrical device on tooth movement. *Korean Journal of Orthodontics - KOREAN J ORTHOD* 2008;**38**. doi: 10.4041/kjod.2008.38.5.337.

3. Showkatbakhsh R, Jamilian A, Showkatbakhsh M; The effect of pulsed electromagnetic fields on the acceleration of tooth movement. *World J Orthod* 2010;**11**(4):e52-6.

4. Bhad (Patil) WA, Karemore AA; Efficacy of pulsed electromagnetic field in reducing treatment time: A clinical investigation. *Am J Orthod Dentofacial Orthop* 2022;**161**(5):652-658. doi: 10.1016/j.ajodo.2020.12.025.
