# Supplementary material for: The Effectiveness of Physical Adjunctive Interventions in the Acceleration of Orthodontic Tooth Movement: An Umbrella Review and Meta‐Analysis
Source: Int J Dent. 2026 Feb 3;2026:9131541. doi: 10.1155/ijod/9131541 (PMC12868923; doi:10.1155/ijod/9131541)
Supplement: Supplementary file 7 — Supporting Information 7 Table S7: The degree of overlap of primary studies included in all systematic reviews. [file IJOD-2026-9131541-s002.docx]

| **Supplementary Table 7**: The degree of overlap of primary studies included in all systematic reviews. | | | | | | | | | | | | | | | | | | | | | | | |
| --- | --- | --- | --- | --- | --- | --- | --- | --- | --- | --- | --- | --- | --- | --- | --- | --- | --- | --- | --- | --- | --- | --- | --- |
| **Study** | | | **SRs** | | | | | | | | | | | | | | | | | | | | |
|  |  |  | **VD + PBMD** | **BES** | **VD** | | | | | | | | **PBMD** | | | | | | | | | | |
|  |  |  | **El-Angbawi et al, 2023** | **Dutta et al, 2024** | **Aljabaa et al, 2018** | **Abd Elmotaleb et al, 2019** | **Bakdach et al, 2020** | **Keerthana et al, 2020** | | **García Vega et al, 2021** | **Dutta et al, 2025** | **De Almeida et al, 2016** | | **Imani et al, 2018** | **Deana et al, 2019** | | **Bakdach et al, 2020** | **Camacho et al, 2020** | **Garjales et al, 2023** | **Jnaneshwar et al, 2023** | **Malik et al, 2024** | **Hmida et al, 2024** |  |
| Number of Trials (RCTs) | | | **23** | **4** | **6** | **6** | **17** | **12** | | **15** | **21** | **6** | | **6** | **16** | | **25** | **9** | **19** | **10** | **6** | **14** |  |
| **Shared Studies** | **VD** | **Miles et al, 2012** | **✓** | **✗** | **✓** | **✓** | **✓** | **✓** | | **✓** | **✓** | **✗** | | **✗** | **✗** | | **✗** | **✗** | **✗** | **✗** | **✗** | **✗** |  |
|  |  | **Woodhouse et al, 2015** | **✓** | **✗** | **✓** | **✓** | **✓** | **✓** | | **✓** | **✓** | **✗** | | **✗** | **✗** | | **✗** | **✗** | **✗** | **✗** | **✗** | **✗** |  |
|  |  | **Pavilin et al, 2015** | **✓** | **✗** | **✓** | **✓** | **✓** | **✓** | | **✓** | **✓** | **✗** | | **✗** | **✗** | | **✗** | **✗** | **✗** | **✗** | **✗** | **✗** |  |
|  |  | **Miles and Fisher, 2016** | **✓** | **✗** | **✓** | **✓** | **✓** | **✓** | | **✓** | **✓** | **✗** | | **✗** | **✗** | | **✗** | **✗** | **✗** | **✗** | **✗** | **✗** |  |
|  |  | **Dibiase et al, 2018** | **✗** | **✗** | **✓** | **✓** | **✓** | **✓** | | **✓** | **✓** | **✗** | | **✗** | **✗** | | **✗** | **✗** | **✗** | **✗** | **✗** | **✗** |  |
|  |  | **Katchooi et al, 2018** | **✓** | **✗** | **✓** | **✗** | **✓** | **✓** | | **✓** | **✓** | **✗** | | **✗** | **✗** | | **✗** | **✗** | **✗** | **✗** | **✗** | **✗** |  |
|  | **PBMD** | **Cruz et al. (2004)** | **✗** | **✗** | **✗** | **✗** | **✗** | **✗** | | **✗** | **✗** | **✓** | | **✓** | **✓** | | **✓** | **✓** | **✓** | **✗** | **✗** | **✗** |  |
|  |  | **Sousa et al. (2011)** | **✗** | **✗** | **✗** | **✗** | **✗** | **✗** | | **✗** | **✗** | **✓** | | **✓** | **✓** | | **✓** | **✓** | **✓** | **✗** | **✗** | **✗** |  |
|  |  | **Doshi-Mehta et al. (2012)** | **✗** | **✗** | **✗** | **✗** | **✗** | **✗** | | **✗** | **✗** | **✓** | | **✓** | **✓** | | **✓** | **✓** | **✓** | **✓** | **✗** | **✗** |  |
|  |  | **Qamruddin et al. (2017)** | **✗** | **✗** | **✗** | **✗** | **✗** | **✗** | | **✗** | **✗** | **✗** | | **✓** | **✓** | | **✓** | **✗** | **✓** | **✓** | **✗** | **✓** |  |
|  |  | **Alsayed Hasan et al. (2017)** | **✓** | **✗** | **✗** | **✗** | **✗** | **✗** | | **✗** | **✗** | **✗** | | **✗** | **✓** | | **✓** | **✓** | **✗** | **✓** | **✓** | **✓** |  |
|  |  | **Üretürk et al. (2017)** | **✗** | **✗** | **✗** | **✗** | **✗** | **✗** | | **✗** | **✗** | **✗** | | **✓** | **✓** | | **✓** | **✗** | **✓** | **✓** | **✗** | **✓** |  |
|  |  | **Caccianiga G et al. (2017)** | **✓** | **✗** | **✗** | **✗** | **✗** | **✗** | | **✗** | **✗** | **✗** | | **✗** | **✓** | | **✓** | **✗** | **✗** | **✓** | **✓** | **✓** |  |
| **Partially Overlapping Studies** | **VD** | **Leethanakul et al, 2016** | **✗** | **✗** | **✗** | **✗** | **✓** | **✗** | | **✓** | **✓** | **✗** | | **✗** | **✗** | | **✗** | **✗** | **✗** | **✗** | **✗** | **✗** |  |
|  |  | **Alansari et al, 2017** | **✗** | **✗** | **✗** | **✗** | **✓** | **✗** | | **✓** | **✓** | **✗** | | **✗** | **✗** | | **✗** | **✗** | **✗** | **✗** | **✗** | **✗** |  |
|  |  | **Liao et al, 2017** | **✗** | **✗** | **✗** | **✗** | **✓** | **✓** | | **✓** | **✓** | **✗** | | **✗** | **✗** | | **✗** | **✗** | **✗** | **✗** | **✗** | **✗** |  |
|  |  | **Miles et al, 2018** | **✗** | **✗** | **✗** | **✓** | **✓** | **✓** | | **✗** | **✓** | **✗** | | **✗** | **✗** | | **✗** | **✗** | **✗** | **✗** | **✗** | **✗** |  |
|  |  | **Siriphan et al, 2018** | **✓** | **✗** | **✗** | **✗** | **✗** | **✓** | | **✓** | **✓** | **✗** | | **✗** | **✗** | | **✗** | **✗** | **✗** | **✗** | **✗** | **✗** |  |
|  |  | **Azeem et al, 2019** | **✗** | **✗** | **✗** | **✗** | **✓** | **✓** | | **✓** | **✓** | **✗** | | **✗** | **✗** | | **✗** | **✗** | **✗** | **✗** | **✗** | **✗** |  |
|  |  | **Kannan et al, 2019** | **✗** | **✗** | **✗** | **✗** | **✗** | **✓** | | **✓** | **✓** | **✗** | | **✗** | **✗** | | **✗** | **✗** | **✗** | **✗** | **✗** | **✗** |  |
|  |  | **Taha et al, 2019** | **✓** | **✗** | **✗** | **✗** | **✓** | **✓** | | **✓** | **✗** | **✗** | | **✗** | **✗** | | **✗** | **✗** | **✗** | **✗** | **✗** | **✗** |  |
|  |  | **Kumar et al, 2020** | **✓** | **✗** | **✗** | **✗** | **✗** | **✗** | | **✓** | **✓** | **✗** | | **✗** | **✗** | | **✗** | **✗** | **✗** | **✗** | **✗** | **✗** |  |
|  | **PBMD** | **Limpanichkul et al. (2006)** | **✗** | **✗** | **✗** | **✗** | **✗** | **✗** | | **✗** | **✗** | **✗** | | **✗** | **✓** | | **✓** | **✓** | **✓** | **✓** | **✗** | **✗** |  |
|  |  | **Kansal et al. (2014)** | **✗** | **✗** | **✗** | **✗** | **✗** | **✗** | | **✗** | **✗** | **✓** | | **✗** | **✓** | | **✓** | **✓** | **✓** | **✗** | **✗** | **✗** |  |
|  |  | **Heravi et al. (2014)** | **✗** | **✗** | **✗** | **✗** | **✗** | **✗** | | **✗** | **✗** | **✗** | | **✓** | **✓** | | **✓** | **✗** | **✗** | **✗** | **✗** | **✗** |  |
|  |  | **Souza (2014)** | **✗** | **✗** | **✗** | **✗** | **✗** | **✗** | | **✗** | **✗** | **✓** | | **✗** | **✗** | | **✓** | **✗** | **✓** | **✗** | **✗** | **✗** |  |
|  |  | **Pereira (2014)** | **✗** | **✗** | **✗** | **✗** | **✗** | **✗** | | **✗** | **✗** | **✓** | | **✗** | **✗** | | **✓** | **✗** | **✓** | **✗** | **✗** | **✗** |  |
|  |  | **Dalaie K et al. (2015)** | **✗** | **✗** | **✗** | **✗** | **✗** | **✗** | | **✗** | **✗** | **✗** | | **✗** | **✓** | | **✓** | **✗** | **✓** | **✗** | **✓** | **✗** |  |
|  |  | **Yassaei et al. (2016)** | **✗** | **✗** | **✗** | **✗** | **✗** | **✗** | | **✗** | **✗** | **✗** | | **✗** | **✓** | | **✓** | **✗** | **✓** | **✗** | **✗** | **✗** |  |
|  |  | **Kochar GD et al. (2017)** | **✗** | **✗** | **✗** | **✗** | **✗** | **✗** | | **✗** | **✗** | **✗** | | **✗** | **✓** | | **✓** | **✗** | **✓** | **✗** | **✓** | **✗** |  |
|  |  | **Nahas et al. (2017)** | **✓** | **✗** | **✗** | **✗** | **✗** | **✗** | | **✗** | **✗** | **✗** | | **✗** | **✗** | | **✓** | **✗** | **✗** | **✓** | **✓** | **✓** |  |
|  |  | **Arumughan et al. (2018)** | **✗** | **✗** | **✗** | **✗** | **✗** | **✗** | | **✗** | **✗** | **✗** | | **✗** | **✗** | | **✓** | **✓** | **✓** | **✗** | **✗** | **✓** |  |
|  |  | **Guram et al. (2018)** | **✗** | **✗** | **✗** | **✗** | **✗** | **✗** | | **✗** | **✗** | **✗** | | **✗** | **✓** | | **✓** | **✗** | **✓** | **✗** | **✗** | **✓** |  |
|  |  | **Varella et al. (2018)** | **✗** | **✗** | **✗** | **✗** | **✗** | **✗** | | **✗** | **✗** | **✗** | | **✗** | **✗** | | **✓** | **✗** | **✓** | **✓** | **✗** | **✗** |  |
| **Unique Studies** | **VD** | **Chouinard, 2016** | **✗** | **✗** | **✗** | **✗** | **✓** | **✗** | | **✗** | **✗** | **✗** | | **✗** | **✗** | | **✗** | **✗** | **✗** | **✗** | **✗** | **✗** |  |
|  |  | **Bulic, 2017** | **✗** | **✗** | **✗** | **✗** | **✓** | **✗** | | **✗** | **✗** | **✗** | | **✗** | **✗** | | **✗** | **✗** | **✗** | **✗** | **✗** | **✗** |  |
|  |  | **Kalemaj et al, 2017** | **✗** | **✗** | **✗** | **✗** | **✗** | **✗** | | **✗** | **✓** | **✗** | | **✗** | **✗** | | **✗** | **✗** | **✗** | **✗** | **✗** | **✗** |  |
|  |  | **Pescheret, 2017** | **✗** | **✗** | **✗** | **✗** | **✓** | **✗** | | **✗** | **✗** | **✗** | | **✗** | **✗** | | **✗** | **✗** | **✗** | **✗** | **✗** | **✗** |  |
|  |  | **Bragassa, 2018** | **✗** | **✗** | **✗** | **✗** | **✓** | **✗** | | **✗** | **✗** | **✗** | | **✗** | **✗** | | **✗** | **✗** | **✗** | **✗** | **✗** | **✗** |  |
|  |  | **Bisht et al, 2019** | **✗** | **✗** | **✗** | **✗** | **✗** | **✗** | | **✗** | **✓** | **✗** | | **✗** | **✗** | | **✗** | **✗** | **✗** | **✗** | **✗** | **✗** |  |
|  |  | **Lombardo et al, 2019** | **✗** | **✗** | **✗** | **✗** | **✓** | **✓** | | **✗** | **✗** | **✗** | | **✗** | **✗** | | **✗** | **✗** | **✗** | **✗** | **✗** | **✗** |  |
|  |  | **Telatar et al, 2020** | **✗** | **✗** | **✗** | **✗** | **✗** | **✗** | | **✗** | **✓** | **✗** | | **✗** | **✗** | | **✗** | **✗** | **✗** | **✗** | **✗** | **✗** |  |
|  |  | **Reiss et al, 2020** | **✗** | **✗** | **✗** | **✗** | **✗** | **✗** | | **✗** | **✗** | **✗** | | **✗** | **✗** | | **✗** | **✗** | **✗** | **✗** | **✗** | **✗** |  |
|  |  | **Khera et al, 2022** | **✗** | **✗** | **✗** | **✗** | **✗** | **✗** | | **✗** | **✓** | **✗** | | **✗** | **✗** | | **✗** | **✗** | **✗** | **✗** | **✗** | **✗** |  |
|  |  | **Mayama et al, 2022** | **✗** | **✗** | **✗** | **✗** | **✗** | **✗** | | **✗** | **✓** | **✗** | | **✗** | **✗** | | **✗** | **✗** | **✗** | **✗** | **✗** | **✗** |  |
|  |  | **Gujar et al, 2023** | **✗** | **✗** | **✗** | **✗** | **✗** | **✗** | | **✗** | **✓** | **✗** | | **✗** | **✗** | | **✗** | **✗** | **✗** | **✗** | **✗** | **✗** |  |
|  |  | **Yildiz et al, 2024** | **✗** | **✗** | **✗** | **✗** | **✗** | **✗** | | **✗** | **✓** | **✗** | | **✗** | **✗** | | **✗** | **✗** | **✗** | **✗** | **✗** | **✗** |  |
|  | **PBMD** | **Youssef et al. (2008)** | **✗** | **✗** | **✗** | **✗** | **✗** | **✗** | | **✗** | **✗** | **✗** | | **✗** | **✗** | | **✗** | **✓** | **✗** | **✗** | **✗** | **✗** |  |
|  |  | **Hosseini et al. (2011)** | **✗** | **✗** | **✗** | **✗** | **✗** | **✗** | | **✗** | **✗** | **✗** | | **✗** | **✗** | | **✓** | **✗** | **✗** | **✗** | **✗** | **✗** |  |
|  |  | **Kau et al. (2013)** | **✗** | **✗** | **✗** | **✗** | **✗** | **✗** | | **✗** | **✗** | **✗** | | **✗** | **✗** | | **✓** | **✗** | **✗** | **✗** | **✗** | **✗** |  |
|  |  | **Dominguez et al. (2013)** | **✗** | **✗** | **✗** | **✗** | **✗** | **✗** | | **✗** | **✗** | **✗** | | **✗** | **✗** | | **✗** | **✓** | **✗** | **✗** | **✗** | **✗** |  |
|  |  | **Ekizer et al. (2016)** | **✗** | **✗** | **✗** | **✗** | **✗** | **✗** | | **✗** | **✗** | **✗** | | **✗** | **✗** | | **✓** | **✗** | **✗** | **✓** | **✗** | **✗** |  |
|  |  | **Caccianiga et al. (2016)** | **✗** | **✗** | **✗** | **✗** | **✗** | **✗** | | **✗** | **✗** | **✗** | | **✗** | **✗** | | **✓** | **✗** | **✗** | **✗** | **✗** | **✗** |  |
|  |  | **Sandoval et al. (2017)** | **✗** | **✗** | **✗** | **✗** | **✗** | **✗** | | **✗** | **✗** | **✗** | | **✗** | **✓** | | **✗** | **✗** | **✗** | **✗** | **✗** | **✗** |  |
|  |  | **Samara et al. (2018)** | **✗** | **✗** | **✗** | **✗** | **✗** | **✗** | | **✗** | **✗** | **✗** | | **✗** | **✗** | | **✓** | **✗** | **✗** | **✗** | **✗** | **✗** |  |
|  |  | **Al-Okla N et al. (2018)** | **✗** | **✗** | **✗** | **✗** | **✗** | **✗** | | **✗** | **✗** | **✗** | | **✗** | **✗** | | **✓** | **✗** | **✗** | **✗** | **✓** | **✗** |  |
|  |  | **Mal et al. (2018)** | **✗** | **✗** | **✗** | **✗** | **✗** | **✗** | | **✗** | **✗** | **✗** | | **✗** | **✓** | | **✗** | **✗** | **✗** | **✗** | **✗** | **✗** |  |
|  |  | **Isola et al. (2019)** | **✗** | **✗** | **✗** | **✗** | **✗** | **✗** | | **✗** | **✗** | **✗** | | **✗** | **✗** | | **✗** | **✗** | **✗** | **✗** | **✗** | **✓** |  |
|  |  | **Alam et al (2019)** | **✓** | **✗** | **✗** | **✗** | **✗** | **✗** | | **✗** | **✗** | **✗** | | **✗** | **✗** | | **✗** | **✗** | **✗** | **✗** | **✗** | **✗** |  |
|  |  | **El Shehawy et al (2020)** | **✓** | **✗** | **✗** | **✗** | **✗** | **✗** | | **✗** | **✗** | **✗** | | **✗** | **✗** | | **✗** | **✗** | **✗** | **✗** | **✗** | **✗** |  |
|  |  | **Giudice et al (2020)** | **✓** | **✗** | **✗** | **✗** | **✗** | **✗** | | **✗** | **✗** | **✗** | | **✗** | **✗** | | **✗** | **✗** | **✗** | **✗** | **✗** | **✓** |  |
|  |  | **Mistry et al. (2020)** | **✗** | **✗** | **✗** | **✗** | **✗** | **✗** | | **✗** | **✗** | **✗** | | **✗** | **✗** | | **✗** | **✗** | **✓** | **✓** | **✗** | **✗** |  |
|  |  | **Lahunpuli et al. (2020)** | **✓** | **✗** | **✗** | **✗** | **✗** | **✗** | | **✗** | **✗** | **✗** | | **✗** | **✗** | | **✗** | **✗** | **✓** | **✗** | **✗** | **✗** |  |
|  |  | **Impellizzeri et al. (2020)** |  | **✗** | **✗** | **✗** | **✗** | **✗** | | **✗** | **✗** | **✗** | | **✗** | **✗** | | **✗** | **✗** | **✓** | **✗** | **✗** | **✗** |  |
|  |  | **Abellán et al (2021)** | **✓** | **✗** | **✗** | **✗** | **✗** | **✗** | | **✗** | **✗** | **✗** | | **✗** | **✗** | | **✗** | **✗** | **✗** | **✗** | **✗** | **✗** |  |
|  |  | **Farhadian et al (2021)** | **✓** | **✗** | **✗** | **✗** | **✗** | **✗** | | **✗** | **✗** | **✗** | | **✗** | **✗** | | **✗** | **✗** | **✗** | **✗** | **✗** | **✗** |  |
|  |  | **Zheng et al. (2021)** | **✗** | **✗** | **✗** | **✗** | **✗** | **✗** | | **✗** | **✗** | **✗** | | **✗** | **✗** | | **✗** | **✗** | **✓** | **✗** | **✗** | **✓** |  |
|  |  | **Pérignon et al., 2021** | **✗** | **✗** | **✗** | **✗** | **✗** | **✗** | | **✗** | **✗** | **✗** | | **✗** | **✗** | | **✗** | **✗** | **✗** | **✗** | **✗** | **✓** |  |
|  |  | **Al Shafi et al. 2021** | **✗** | **✗** | **✗** | **✗** | **✗** | **✗** | | **✗** | **✗** | **✗** | | **✗** | **✗** | | **✗** | **✗** | **✗** | **✗** | **✗** | **✓** |  |
|  |  | **Gaffar et al. 2022** | **✓** | **✗** | **✗** | **✗** | **✗** | **✗** | | **✗** | **✗** | **✗** | | **✗** | **✗** | | **✗** | **✗** | **✗** | **✗** | **✗** | **✓** |  |
|  |  | **Hasan et al (2022)** | **✓** | **✗** | **✗** | **✗** | **✗** | **✗** | | **✗** | **✗** | **✗** | | **✗** | **✗** | | **✗** | **✗** | **✗** | **✗** | **✗** | **✗** |  |
|  |  | **Kharat et al., 2023** | **✗** | **✗** | **✗** | **✗** | **✗** | **✗** | | **✗** | **✗** | **✗** | | **✗** | **✗** | | **✗** | **✗** | **✗** | **✗** | **✗** | **✓** |  |
|  | **BES** | **Kim et al. 2008** | **✗** | **✓** | **✗** | **✗** | **✗** | **✗** | | **✗** | **✗** | **✗** | | **✗** | **✗** | | **✗** | **✗** | **✗** | **✗** | **✗** | **✗** |  |
|  |  | **Showkatbakhsh et al. 2010** | **✗** | **✓** | **✗** | **✗** | **✗** | **✗** | | **✗** | **✗** | **✗** | | **✗** | **✗** | | **✗** | **✗** | **✗** | **✗** | **✗** | **✗** |  |
|  |  | **Jung et al. 2017** | **✗** | **✓** | **✗** | **✗** | **✗** | **✗** | | **✗** | **✗** | **✗** | | **✗** | **✗** | | **✗** | **✗** | **✗** | **✗** | **✗** | **✗** |  |
|  |  | **Barsi et al. 2023** | **✗** | **✓** | **✗** | **✗** | **✗** | **✗** | | **✗** | **✗** | **✗** | | **✗** | **✗** | | **✗** | **✗** | **✗** | **✗** | **✗** | **✗** |  |
| **Abbreviations Reference** | | | | | | | | | **Canonical Correspondence Analysis (CCA) Data Summary** | | | | | | | **Color Coding, Signals** | | | | | | | |
| **SRs**: systematic reviews; **RCT**: randomized controlled trial; **BES**: Bioelectric Stimulation; **VD**: vibration device; **PBMD**: Photobiomodulation  **\** | | | | | | | | | **Number of included publications** (**N**) = 215  **Number of rows** (**r**) = 76  **Number of columns** (**c**) = 17  **CCA= 0.11** | | | | | | | **Green background**: Studies are common in **6 ≤ columns**. **Yellow background**: Studies overlapping in **3–5 columns**. **White background**: Unique studies (only in **1-2 columns**).  **✓** = Study present in the column.  **✗ =** Study not present in the column | | | | | | | |
