# Supplementary material for: The Effectiveness of Physical Adjunctive Interventions in the Acceleration of Orthodontic Tooth Movement: An Umbrella Review and Meta‐Analysis
Source: Int J Dent. 2026 Feb 3;2026:9131541. doi: 10.1155/ijod/9131541 (PMC12868923; doi:10.1155/ijod/9131541)
Supplement: Supplementary file 8 — Supporting Information 8 Table S8: The degree of overlap of primary studies included in systematic reviews of VDs. [file IJOD-2026-9131541-s001.docx]

| **Supplementary Table 8**: The degree of overlap of primary studies included in systematic reviews of **VDs** | | | | | | | | |
| --- | --- | --- | --- | --- | --- | --- | --- | --- |
| **SRs** | | | | | | | | |
| Study | | **Aljabaa et al, 2018** | **Abd Elmotaleb et al, 2019** | **Bakdach et al, 2020** | **Keerthana et al, 2020** | **García Vega et al, 2021** | **El-Angbawi et al, 2023** | **Dutta et al, 2025** |
| Number of Trials (RCTs) | | **6** | **6** | **17** | **12** | **15** | **12** | **21** |
| Shared Studies | **Miles et al, 2012** | **✓** | **✓** | **✓** | **✓** | **✓** | **✓** | **✓** |
|  | **Woodhouse et al, 2015** | **✓** | **✓** | **✓** | **✓** | **✓** | **✓** | **✓** |
|  | **Pavilin et al, 2015** | **✓** | **✓** | **✓** | **✓** | **✓** | **✓** | **✓** |
|  | **Miles and Fisher, 2016** | **✓** | **✓** | **✓** | **✓** | **✓** | **✓** | **✓** |
|  | **Dibiase et al, 2018** | **✓** | **✓** | **✓** | **✓** | **✓** | **✗** | **✓** |
|  | **Katchooi et al, 2018** | **✓** | **✗** | **✓** | **✓** | **✓** | **✓** | **✓** |
| Partially Overlapping Studies | **Leethanakul et al, 2016** | **✗** | **✗** | **✓** | **✗** | **✓** | **✗** | **✓** |
|  | **Alansari et al, 2017** | **✗** | **✗** | **✓** | **✗** | **✓** | **✗** | **✓** |
|  | **Liao et al, 2017** | **✗** | **✗** | **✓** | **✓** | **✓** | **✗** | **✓** |
|  | **Miles et al, 2018** | **✗** | **✓** | **✓** | **✓** | **✗** | **✗** | **✓** |
|  | **Siriphan et al, 2018** | **✗** | **✗** | **✗** | **✓** | **✓** | **✓** | **✓** |
|  | **Azeem et al, 2019** | **✗** | **✗** | **✓** | **✓** | **✓** | **✗** | **✓** |
|  | **Kannan et al, 2019** | **✗** | **✗** | **✗** | **✓** | **✓** | **✗** | **✓** |
|  | **Taha et al, 2019** | **✗** | **✗** | **✓** | **✓** | **✓** | **✓** | **✗** |
|  | **Kumar et al, 2020** | **✗** | **✗** | **✗** | **✗** | **✓** | **✓** | **✓** |
| Unique Studies | **Chouinard, 2016** | **✗** | **✗** | **✓** | **✗** | **✗** | **✗** | **✗** |
|  | **Bulic, 2017** | **✗** | **✗** | **✓** | **✗** | **✗** | **✗** | **✗** |
|  | **Kalemaj et al, 2017** | **✗** | **✗** | **✗** | **✗** | **✗** | **✗** | **✓** |
|  | **Pescheret, 2017** | **✗** | **✗** | **✓** | **✗** | **✗** | **✗** | **✗** |
|  | **Bragassa, 2018** | **✗** | **✗** | **✓** | **✗** | **✗** | **✓** | **✗** |
|  | **Bisht et al, 2019** | **✗** | **✗** | **✗** | **✗** | **✗** | **✗** | **✓** |
|  | **Lombardo et al, 2019** | **✗** | **✗** | **✓** | **✗** | **✓** | **✓** | **✗** |
|  | **Telatar et al, 2020** | **✗** | **✗** | **✗** | **✗** | **✗** | **✓** | **✓** |
|  | **Reiss et al, 2020** | **✗** | **✗** | **✗** | **✗** | **✗** | **✓** | **✗** |
|  | **Khera et al, 2022** | **✗** | **✗** | **✗** | **✗** | **✗** | **✗** | **✓** |
|  | **Mayama et al, 2022** | **✗** | **✗** | **✗** | **✗** | **✗** | **✗** | **✓** |
|  | **Gujar et al, 2023** | **✗** | **✗** | **✗** | **✗** | **✗** | **✗** | **✓** |
|  | **Yildiz et al, 2024** | **✗** | **✗** | **✗** | **✗** | **✗** | **✗** | **✓** |
| **Abbreviations Reference** | | | **Canonical Correspondence Analysis (CCA) Data Summary** | | | **Color Coding, Signals** | | |
| **SRs**: systematic reviews; **RCT**: randomized controlled trial; **VD**: vibration device. | | | **Number of included publications** (**N**) = 89  **Number of rows** (**r**) = 28  **Number of columns** (**c**) = 7  **CCA= 0.36** | | | **Green background**: Studies are common in **6 ≤ columns**. **Yellow background**: Studies overlapping in **3–5 columns**. **White background**: Unique studies (only in **1-2 columns**).  **✓** = Study present in the column.  **✗ =** Study not present in the column | | |
