# Supplementary material for: The Effectiveness of Physical Adjunctive Interventions in the Acceleration of Orthodontic Tooth Movement: An Umbrella Review and Meta‐Analysis
Source: Int J Dent. 2026 Feb 3;2026:9131541. doi: 10.1155/ijod/9131541 (PMC12868923; doi:10.1155/ijod/9131541)
Supplement: Supplementary file 9 — Supporting Information 9 Table S9: The degree of overlap of primary studies included in systematic reviews of PBM. [file IJOD-2026-9131541-s014.docx]

| **Supplementary Table 9**: The degree of overlap of primary studies included in systematic reviews of **PBM** | | | | | | | | | | | | | |
| --- | --- | --- | --- | --- | --- | --- | --- | --- | --- | --- | --- | --- | --- |
| **SRs** | | | | | | | | | | | | | |
| **Study** | | **De Almeida et al, 2016** | **Imani et al, 2018** | **Deana et al, 2019** | **Bakdach et al, 2020** | **Camacho et al, 2020** | **Garjales et al, 2023** | **Jnaneshwar et al, 2023** | | **El-Angbawi et al, 2023** | **Malik et al, 2024** | **Hmida et al, 2024** |  |
| **Number of Included RCTs** | | **6** | **6** | **16** | **25** | **9** | **19** | **10** | | **11** | **6** | **14** |  |
| Shared Studies | **Cruz et al. (2004)** | **✔️** | **✔️** | **✔️** | **✔️** | **✔️** | **✔️** | **❌** | | **❌** | **❌** | **❌** |  |
|  | **Sousa et al. (2011)** | **✔️** | **✔️** | **✔️** | **✔️** | **✔️** | **✔️** | **❌** | | **❌** | **❌** | **❌** |  |
|  | **Doshi-Mehta et al. (2012)** | **✔️** | **✔️** | **✔️** | **✔️** | **✔️** | **✔️** | **✔️** | | **❌** | **❌** | **❌** |  |
|  | **Qamruddin et al. (2017)** | **❌** | **✔️** | **✔️** | **✔️** | **❌** | **✔️** | **✔️** | | **❌** | **❌** | **✔️** |  |
|  | **Alsayed Hasan et al. (2017)** | **❌** | **❌** | **✔️** | **✔️** | **✔️** | **❌** | **✔️** | | **✔️** | **✔️** | **✔️** |  |
|  | **Üretürk et al. (2017)** | **❌** | **✔️** | **✔️** | **✔️** | **❌** | **✔️** | **✔️** | | **❌** | **❌** | **✔️** |  |
|  | **Caccianiga G et al. (2017)** | **❌** | **❌** | **✔️** | **✔️** | **❌** | **❌** | **✔️** | | **✔️** | **✔️** | **✔️** |  |
| Partially Overlapping Studies | **Limpanichkul et al. (2006)** | **❌** | **❌** | **✔️** | **✔️** | **✔️** | **✔️** | **✔️** | | **❌** | **❌** | **❌** |  |
|  | **Kansal et al. (2014)** | **✔️** | **❌** | **✔️** | **✔️** | **✔️** | **✔️** | **❌** | | **❌** | **❌** | **❌** |  |
|  | **Heravi et al. (2014)** | **❌** | **✔️** | **✔️** | **✔️** | **❌** | **❌** | **❌** | | **❌** | **❌** | **❌** |  |
|  | **Souza (2014)** | **✔️** | **❌** | **❌** | **✔️** | **❌** | **✔️** | **❌** | | **❌** | **❌** | **❌** |  |
|  | **Pereira (2014)** | **✔️** | **❌** | **❌** | **✔️** | **❌** | **✔️** | **❌** | | **❌** | **❌** | **❌** |  |
|  | **Dalaie K et al. (2015)** | **❌** | **❌** | **✔️** | **✔️** | **❌** | **✔️** | **❌** | | **❌** | **✔️** | **❌** |  |
|  | **Yassaei et al. (2016)** | **❌** | **❌** | **✔️** | **✔️** | **❌** | **✔️** | **❌** | | **❌** | **❌** | **❌** |  |
|  | **Kochar GD et al. (2017)** | **❌** | **❌** | **✔️** | **✔️** | **❌** | **✔️** | **❌** | | **❌** | **✔️** | **❌** |  |
|  | **Nahas et al. (2017)** | **❌** | **❌** | **❌** | **✔️** | **❌** | **❌** | **✔️** | | **✔️** | **✔️** | **✔️** |  |
|  | **Arumughan et al. (2018)** | **❌** | **❌** | **❌** | **✔️** | **✔️** | **✔️** | **❌** | | **❌** | **❌** | **✔️** |  |
|  | **Guram et al. (2018)** | **❌** | **❌** | **✔️** | **✔️** | **❌** | **✔️** | **❌** | | **❌** | **❌** | **✔️** |  |
|  | **Varella et al. (2018)** | **❌** | **❌** | **❌** | **✔️** | **❌** | **✔️** | **✔️** | | **❌** | **❌** | **❌** |  |
| Unique Studies | **Youssef et al. (2008)** | **❌** | **❌** | **❌** | **❌** | **✔️** | **❌** | **❌** | | **❌** | **❌** | **❌** |  |
|  | **Hosseini et al. (2011)** | **❌** | **❌** | **❌** | **✔️** | **❌** | **❌** | **❌** | | **❌** | **❌** | **❌** |  |
|  | **Kau et al. (2013)** | **❌** | **❌** | **❌** | **✔️** | **❌** | **❌** | **❌** | | **❌** | **❌** | **❌** |  |
|  | **Dominguez et al. (2013)** | **❌** | **❌** | **❌** | **❌** | **✔️** | **❌** | **❌** | | **❌** | **❌** | **❌** |  |
|  | **Ekizer et al. (2016)** | **❌** | **❌** | **❌** | **✔️** | **❌** | **❌** | **✔️** | | **❌** | **❌** | **❌** |  |
|  | **Caccianiga et al. (2016)** | **❌** | **❌** | **❌** | **✔️** | **❌** | **❌** | **❌** | | **❌** | **❌** | **❌** |  |
|  | **Sandoval et al. (2017)** | **❌** | **❌** | **✔️** | **❌** | **❌** | **❌** | **❌** | | **❌** | **❌** | **❌** |  |
|  | **Samara et al. (2018)** | **❌** | **❌** | **❌** | **✔️** | **❌** | **❌** | **❌** | | **❌** | **❌** | **❌** |  |
|  | **Al-Okla N et al. (2018)** | **❌** | **❌** | **❌** | **✔️** | **❌** | **❌** | **❌** | | **❌** | **✔️** | **❌** |  |
|  | **Mal et al. (2018)** | **❌** | **❌** | **✔️** | **❌** | **❌** | **❌** | **❌** | | **❌** | **❌** | **❌** |  |
|  | **Isola et al. (2019)** | **❌** | **❌** | **❌** | **❌** | **❌** | **❌** | **❌** | | **❌** | **❌** | **✔️** |  |
|  | **Alam et al (2019)** | **❌** | **❌** | **❌** | **❌** | **❌** | **❌** | **❌** | | **✔️** | **❌** | **❌** |  |
|  | **El Shehawy et al (2020)** | **❌** | **❌** | **❌** | **❌** | **❌** | **❌** | **❌** | | **✔️** | **❌** | **❌** |  |
|  | **Giudice et al (2020)** | **❌** | **❌** | **❌** | **❌** | **❌** | **❌** | **❌** | | **✔️** | **❌** | **✔️** |  |
|  | **Mistry et al. (2020)** | **❌** | **❌** | **❌** | **❌** | **❌** | **✔️** | **✔️** | | **❌** | **❌** | **❌** |  |
|  | **Lahunpuli et al. (2020)** | **❌** | **❌** | **❌** | **❌** | **❌** | **✔️** | **❌** | | **✔️** | **❌** | **❌** |  |
|  | **Impellizzeri et al. (2020)** | **❌** | **❌** | **❌** | **❌** | **❌** | **✔️** | **❌** | |  | **❌** | **❌** |  |
|  | **Abellán et al (2021)** | **❌** | **❌** | **❌** | **❌** | **❌** | **❌** | **❌** | | **✔️** | **❌** | **❌** |  |
|  | **Farhadian et al (2021)** | **❌** | **❌** | **❌** | **❌** | **❌** | **❌** | **❌** | | **✔️** | **❌** | **❌** |  |
|  | **Zheng et al. (2021)** | **❌** | **❌** | **❌** | **❌** | **❌** | **✔️** | **❌** | | **❌** | **❌** | **✔️** |  |
|  | **Pérignon et al., 2021** | **❌** | **❌** | **❌** | **❌** | **❌** | **❌** | **❌** | | **❌** | **❌** | **✔️** |  |
|  | **Al Shafi et al. 2021** | **❌** | **❌** | **❌** | **❌** | **❌** | **❌** | **❌** | | **❌** | **❌** | **✔️** |  |
|  | **Gaffar et al. 2022** | **❌** | **❌** | **❌** | **❌** | **❌** | **❌** | **❌** | | **✔️** | **❌** | **✔️** |  |
|  | **Hasan et al (2022)** | **❌** | **❌** | **❌** | **❌** | **❌** | **❌** | **❌** | | **✔️** | **❌** | **❌** |  |
|  | **Kharat et al., 2023** | **❌** | **❌** | **❌** | **❌** | **❌** | **❌** | **❌** | | **❌** | **❌** | **✔️** |  |
| **Abbreviations Reference** | | | **Canonical Correspondence Analysis (CCA) Data Summary** | | | | | | **Color Coding, Signals** | | | | |
| **SRs**: systematic reviews; **RCTs**: randomized controlled trials; **PBMD**: Photobiomodulation. | | | **Number of included publications** (**N**) = 122  **Number of rows** (**r**) = 44  **Number of columns** (**c**) = 10  **CCA= 0.19** | | | | | | **Green background**: Studies are common in **6 ≤ columns**. **Yellow background**: Studies overlapping in **3–5 columns**. **White background**: Unique studies (only in **1-2 columns**).  **✓** = Study present in the column.  **✗ =** Study not present in the column | | | | |
