# Supplementary material for: The Effectiveness of Physical Adjunctive Interventions in the Acceleration of Orthodontic Tooth Movement: An Umbrella Review and Meta‐Analysis
Source: Int J Dent. 2026 Feb 3;2026:9131541. doi: 10.1155/ijod/9131541 (PMC12868923; doi:10.1155/ijod/9131541)
Supplement: Supplementary file 10 — Supporting Information 10 Table S10: Detailed supporting reasons for the AMSTAR‐2 (A Measurement Tool to Assess Systematic Reviews‐2) assessment of each included systematic review. [file IJOD-2026-9131541-s015.docx]

| **Supplementary Table 10:** Detailed supporting reasons for the AMSTAR-2 (A Measurement Tool to Assess Systematic Reviews-2) assessment of each included systematic review | | | | | | | | | | | | | | | | | | | | |
| --- | --- | --- | --- | --- | --- | --- | --- | --- | --- | --- | --- | --- | --- | --- | --- | --- | --- | --- | --- | --- |
| **Questions** | **General PAI** | **BES** | **VDs** | | | | | | | **PBMD** | | | | | | | | | | |
|  | **El-Angbawi et al, 2023, UK** | **Dutta et al., 2024, India** | **Al jabaa et al. 2018, Saudi Arabia/USA** | **Abd Elmotaleb et al., 2019, Egypt** | **Bakdach et al. 2020, Syria** | **Keerthana et al. 2020, India** | **García Vega et al.2021, Mexico** | **Dutta et al. 2025, India** | **De Almeida et al., 2016, Brazil** | | **Imani et al. 2018, I*r*an** | **Deana et al. 2019, Chile** | **Bakdach et al. 2020, Syria** | **Camacho et al.2020, Colombia** | **Grajales et al. 2023, Spain** | **Jnaneshwar et al. 2023, India** | **Malik et al. 2024, India** | **Hmida et al. 2024, Tunisia** |  |  |
| **Q1. PICO** | **Yes** | **Yes** | **Yes** | **Yes** | **Yes** | **Yes** | **Yes** | **Yes** | **Yes** | | **Yes** | **Yes** | **Yes** | **Yes** | **Yes** | **Yes** | **Yes** | **Yes** |  |  |
| **Q2. Protocol** | **Yes**  Cochrane protocol (CD010887). Deviations were acknowledged in the "Differences between protocol and review" section. | **Partial Yes**  Justification: PROSPERO (CRD42023495077); PRISMA 2020.  Shortcomings: Deviations from protocol not stated/justified. | **No**  No pre-registered protocol or predefined review steps; deviations from methodology are not stated/justified | **Partial Yes**  Justification: Evidence-Based Center (14-2018); PRISMA guidelines.  Shortcomings: No pre-specified variance analysis plan; deviations not justified | **No**  No pre-registered protocol or predefined review steps; deviations from methodology are not stated/justified | **Partial Yes**  Justification: PROSPERO (CRD42020169675); PRISMA 2020.  Shortcomings: Deviations from protocol not stated/justified | **Partial Yes**  Justification: PROSPERO (CRD42021245217); PRISMA 2020.  Shortcomings: Deviations from protocol not stated/justified | **Partial Yes**  PROSPERO (CRD42024542014)  Shortcomings: Deviations from protocol not stated/justified | **Partial Yes**  Justification: PROSPERO (CRD42015025009); PRISMA 2020.  Shortcomings: Deviations from protocol not stated/justified | | **No**  PRISMA 2020.  No pre-registered protocol or predefined review steps.  deviations from methodology are not stated/justified | **No**  PRISMA 2020.  No pre-registered protocol or predefined review steps.  deviations from methodology are not stated/justified. | **No**  PRISMA 2020.  No pre-registered protocol or predefined review steps.  deviations from methodology are not stated/justified | **Partial Yes**  Justification: PROSPERO (CRD42019117648); PRISMA 2020.  Shortcomings: Deviations from protocol not stated/justified | **Partial Yes**  Justification: PROSPERO (CRD42022332585); PRISMA 2020.  Shortcomings: Deviations from protocol not stated/justified | **Partial Yes**  PROSPERO (CRD42020196472)  Shortcomings: Deviations from protocol not stated/justified | **No**  PRISMA 2020.  No pre-registered protocol or predefined review steps.  deviations from methodology are not stated/justified | **No**  PRISMA 2020.  No pre-registered protocol or predefined review steps.  deviations from methodology are not stated/justified |  |  |
| **Q3. Did the review authors explain their selection of the study designs for inclusion in the review?** | **Yes**  The authors specified the inclusion of RCTs in their eligibility criteria and justified excluding other designs | **Yes**  The authors specified the inclusion of RCTs in their eligibility criteria and justified excluding other designs | **Yes**  The authors specified the inclusion of RCTs in their eligibility criteria and justified excluding other designs | **No**  The review focused on RCTs but did not justify excluding other designs (e.g., observational studies). | **Yes**  The authors specified the inclusion of RCTs in their eligibility criteria and justified excluding other designs | **Yes**  The authors specified the inclusion of RCTs in their eligibility criteria and justified excluding other designs | **Yes**  The authors specified the inclusion of RCTs in their eligibility criteria and justified excluding other designs | **Yes**  The authors specified the inclusion of RCTs in their eligibility criteria and justified excluding other designs | **Yes**  The authors specified the inclusion of RCTs in their eligibility criteria and justified excluding other designs | | **Yes**  The authors specified the inclusion of RCTs in their eligibility criteria and justified excluding other designs | **Yes**  The authors specified the inclusion of RCTs in their eligibility criteria and justified excluding other designs | **Yes**  The authors specified the inclusion of RCTs in their eligibility criteria and justified excluding other designs | **Yes**  The authors specified the inclusion of RCTs in their eligibility criteria and justified excluding other designs | **Yes**  The authors specified the inclusion of RCTs in their eligibility criteria and justified excluding other designs | **Yes**  The authors specified the inclusion of RCTs in their eligibility criteria and justified excluding other designs | **Yes**  The authors specified the inclusion of RCTs in their eligibility criteria and justified excluding other designs | **Yes**  The authors specified the inclusion of RCTs in their eligibility criteria and justified excluding other designs |  |  |
| **Q4. Did the review authors use a comprehensive literature search strategy?** | **Yes**  The search was conducted across 5 databases + manual search + grey literature and trial registries. | **Partial Yes:**  The search covered 4 databases + manual searching.  Trial registries, grey literature, and expert consultation were not mentioned. | **Partial Yes:**  The search covered 4 databases + manual searching.  Trial registries, grey literature, and expert consultation were not mentioned. | **Partial Yes:**  The search included 3 databases + manual journal search.  Trial registries and grey literature were not mentioned; English-language restriction was not justified. | **Yes**  The search was conducted across 7 databases, + manual search + grey literature + trial registries. | **Partial Yes:**  The search covered 3 databases + manual searching.  Trial registries, grey literature, and expert consultation were not mentioned | **Partial Yes:**  The search covered 6 databases + manual searching.  Trial registries, grey literature, and expert consultation were not mentioned. | **Partial Yes:**  The search covered 5 databases + manual searching.  Trial registries, grey literature, and expert consultation were not mentioned. | **Partial Yes:**  The search covered 6 databases (including OpenGrey for grey literature).  The search did not mention clinical trial registries or review reference lists of included studies. | | **Partial Yes:**  The search covered 5 databases + manual searching.  Trial registries, grey literature, and expert consultation were not mentioned | **Partial Yes:**  The search covered 4 databases + manual searching.  Trial registries, grey literature, and expert consultation were not mentioned | **Yes**  The search was conducted across 7 databases + manual search + grey literature + trial registries. | **Partial Yes:**  The search covered 4 databases + manual searching.  Trial registries, grey literature, and expert consultation were not mentioned | **Partial Yes:**  The search covered 4 databases + manual searching.  Trial registries and expert consultation were not mentioned. | **Partial Yes:**  The search covered 5 databases + manual searching.  Trial registries, grey literature, the last search date (was it updated until the review’s publication date?), and expert consultation were not mentioned | **Partial Yes:**  The search covered 5 databases + manual searching.  Trial registries, grey literature, and expert consultation were not mentioned | **Partial Yes:**  The search covered 4 databases + manual searching.  Trial registries, grey literature, and expert consultation were not mentioned  Unclear if updated within 24 months before completion (search ended 15 Oct 2023; published Nov 2024). |  |  |
| **Q5. Did the review authors perform study selection in duplicate?** | **Yes** | **Yes** | **Yes** | **Yes** | **Yes** | **Yes** | **Yes** | **Yes** | **Yes** | | **Yes** | **Yes** | **Yes** | **Yes** | **Yes** | **Yes** | **Yes** | **Yes** |  |  |
| **Q6. Did the review authors perform data extraction in duplicate?** | **Yes** | **Yes** | **Yes** | **Yes** | **Yes** | **Yes** | **Yes** | **Yes** | **Yes** | | **No**  It was noted that one author (M.S) conducted the research and data extraction, while two authors reviewed only the titles and abstracts. | **Yes** | **Yes** | **No**  It was not explicitly stated that data extraction was conducted by independent reviewers or with a mechanism to verify agreement. | **Yes** | **Yes** | **No**  It was mentioned that one author extracted the data, which was then reviewed by two other authors, but it was not specified whether an 80% or higher agreement was achieved in the sample. | **No**  Section 2.3 did not mention that data extraction was performed by independent reviewers, but instead referred to the use of "data extraction models" without details on repetition. |  |  |
| **Q7. Did the review authors provide a list of excluded studies and justify the exclusions?** | **Yes** | **No**  The exclusion of 7 animal studies and two other studies was mentioned; however, neither a complete list of excluded studies nor a detailed justification for each exclusion was provided. | **Yes** | **No**  The number of excluded studies and general reasons (e.g., being group studies) were clarified, but without a detailed list or justification per study.  . | **Yes** | **Partial yes**  The excluded studies were included in Appendix 3, but without full details for each (only the author's name). | **Yes** | **No**  A detailed list of excluded studies, along with the reasons for exclusion, was not provided, despite including a PRISMA flowchart. | **No**  A detailed list of excluded studies, along with the reasons for exclusion, was not provided, despite including a PRISMA flowchart. | | **No**  A detailed list of excluded studies, along with the reasons for exclusion, was not provided, despite including a PRISMA flowchart. | **No**  A detailed list of excluded studies, along with the reasons for exclusion, was not provided, despite including a PRISMA flowchart. | **Yes** | **Yes** | **Yes** | **No**  A detailed list of excluded studies, along with the reasons for exclusion, was not provided, despite including a PRISMA flowchart. | **No**  A list of studies excluded was not provided, despite mentioning general exclusion reasons. | **No**  A list of studies excluded was not provided, despite mentioning general exclusion reasons. |  |  |
| **Q8. Did the review authors describe the included studies in adequate detail?** | **Yes** | **Yes** | **Yes** | **Partial yes**  Table 2 provided details on the sample and interventions but lacked precise descriptions of the temporal settings or dosages. | **Yes** | **Yes** | **Yes** | **Yes** | **Partial yes**  The study characteristics were outlined in the tables, but certain details (e.g., device parameters) remain unclear. | | **Yes** | **Yes** | **Yes** | **Yes** | **Yes** | **Yes** | **Yes** | **Yes** |  |  |
| **Q9. Did the review authors use a satisfactory technique for assessing the risk of bias (RoB) in individual studies that were included in the review?** | **Partial yes**  The 2023 review used the ROB1 tool for its included RCTs, noting that the more appropriate ROB2 tool was released in 2019. | **Yes**  The 2024 review used the ROB2 tool for its included RCTs | **Yes**  The 2018 review used the ROB1 tool for its included RCTs | **Yes**  The 2019 review used the ROB1 tool for its included RCTs | **Partial yes**  The 2020 review used the ROB1 tool for its included RCTs, noting that the more appropriate ROB2 tool was released in 2019. | **Partial yes**  The 2020 review used the ROB1 tool for its included RCTs, noting that the more appropriate ROB2 tool was released in 2019. | **Partial yes**  The 2021 review used the ROB1 tool for its included RCTs, noting that the more appropriate ROB2 tool was released in 2019. | **Yes**  The 2024 review used the ROB2 tool for its included RCTs | **No**  The selection criteria and scores tool, adapted from Cericato et al. [27], was used, but it is not valid. | | **Yes**  The 2018 review used the ROB1 tool for its included RCTs | **Yes**  The 2019 review used the ROB1 tool for its included RCTs | **Partial yes**  The 2020 review used the ROB1 tool for its included RCTs, noting that the more appropriate ROB2 tool was released in 2019. | **Partial yes**  The 2020 review used the ROB1 tool for its included RCTs, noting that the more appropriate ROB2 tool was released in 2019. | **Yes**  The 2023 review used the ROB2 tool for its included RCTs | **Yes**  The 2023 review used the ROB2 tool for its included RCTs | **Partial yes**  The 2024 review used the ROB1 tool for its included RCTs, noting that the more appropriate ROB2 tool was released in 2019. | **Yes**  The 2024 review used the ROB2 tool for its included RCTs |  |  |
| **Q10. Did the review authors report on the sources of funding for the studies included in the review?** | **Yes** | **No**  It is not mentioned whether the included preliminary studies disclosed their funding sources. | **Yes** | **Yes** | **No**  It is not mentioned whether the included preliminary studies disclosed their funding sources. | **No**  It is not mentioned whether the included preliminary studies disclosed their funding sources. | **No**  It is not mentioned whether the included preliminary studies disclosed their funding sources. | **No**  It is not mentioned whether the included preliminary studies disclosed their funding sources. | **No**  It is not mentioned whether the included preliminary studies disclosed their funding sources. | | **No**  It is not mentioned whether the included preliminary studies disclosed their funding sources. | **No**  It is not mentioned whether the included preliminary studies disclosed their funding sources. | **Yes** | **No**  It is not mentioned whether the included preliminary studies disclosed their funding sources. | **No**  It is not mentioned whether the included preliminary studies disclosed their funding sources. | **No**  It is not mentioned whether the included preliminary studies disclosed their funding sources. | **No**  It is not mentioned whether the included preliminary studies disclosed their funding sources. | **No**  It is not mentioned whether the included preliminary studies disclosed their funding sources. |  |  |
| **Q11. If meta-analysis was performed did the review authors use appropriate methods for statistical combination of results?** | **Yes** | **Yes** | A meta-analysis was not conducted due to heterogeneity in study designs and devices used. Instead, qualitative synthesis was employed. | **Yes** | A meta-analysis was not conducted due to heterogeneity in study designs and devices used. Instead, qualitative synthesis was employed. | **Yes** | A meta-analysis was not conducted due to heterogeneity in study designs and devices used. Instead, qualitative synthesis was employed. | **Yes** | **Yes** | | **Yes** | **Yes** | **Yes** | A meta-analysis was not conducted due to heterogeneity in study designs and devices used. Instead, qualitative synthesis was employed. | **Yes** | **Yes** | **Yes** | A meta-analysis was not conducted due to heterogeneity in study designs and devices used. Instead, qualitative synthesis was employed. |  |  |
| **Q12. If meta-analysis was performed, did the review authors assess the potential impact of RoB in individual studies on the results of the meta-analysis or other evidence synthesis?** | **Yes**  The evidence quality was assessed using GRADE, and a sensitivity analysis was conducted. | **No**  Sensitivity analysis was not conducted to assess the impact of bias risk on the results. | A meta-analysis was not conducted due to heterogeneity in study designs and devices used. Instead, qualitative synthesis was employed. | **No**  The evidence quality was not assessed using GRADE.  Sensitivity analysis was not conducted to assess the impact of bias risk on the results. | A meta-analysis was not conducted due to heterogeneity in study designs and devices used. Instead, qualitative synthesis was employed. | **Yes**  The evidence quality was assessed using GRADE, and a Publication Bias was conducted. | A meta-analysis was not conducted due to heterogeneity in study designs and devices used. Instead, qualitative synthesis was employed. | **Yes**  The evidence quality was assessed using GRADE, sensitivity analysis, and a Publication Bias was conducted. | **No**  The evidence quality was not assessed using GRADE.  Sensitivity analysis was not conducted to assess the impact of bias risk on the results. | | **No**  Sensitivity analysis was not conducted to assess the impact of bias risk on the results. | **No**  Sensitivity analysis was not conducted to assess the impact of bias risk on the results. | **Yes**  The evidence quality was assessed using GRADE, and a Publication Bias was conducted. | A meta-analysis was not conducted due to heterogeneity in study designs and devices used. Instead, qualitative synthesis was employed. | **No**  The evidence quality was not assessed using GRADE.  Sensitivity analysis was not conducted to assess the impact of bias risk on the results. | **Yes**  The evidence quality was assessed using GRADE, and the absence of sensitivity analyses was due to the high homogeneity among studies. | **No**  The evidence quality was not assessed using GRADE.  Sensitivity analysis was not conducted to assess the impact of bias risk on the results. | A meta-analysis was not conducted due to heterogeneity in study designs and devices used. Instead, qualitative synthesis was employed. |  |  |
| **Q13. Did the review authors account for RoB in individual studies when interpreting/ discussing the results of the review?** | **Yes** | **Yes** | **Yes** | **No**  It was noted that some studies carry a high risk of bias (e.g., funding), but their impact on the outcomes was not explicitly addressed. | **Yes** | **No**  It was noted that some studies carry a high risk of bias, but their impact on the outcomes was not explicitly addressed. | **Yes** | **Yes** | **No**  The general limitations of the review (e.g., small sample sizes) were discussed, but the risk of bias in individual studies was not linked to the interpretation of the results. | | **No**  It was noted that some studies carry a high risk of bias, but their impact on the outcomes was not explicitly addressed. | **Yes** | **Yes** | **Yes** | **No**  It was noted that some studies carry a high risk of bias, but their impact on the outcomes was not explicitly addressed. | **Yes** | **Yes** | **Yes** |  |  |
| **Q14. Did the review authors provide a satisfactory explanation for, and discussion of, any heterogeneity observed in the results of the review?** | **Yes** | **Yes** | **Yes** | **Yes** | **Yes** | **Yes** | **Yes** | **Yes** | **Yes** | | **Yes** | **Yes** | **Yes** | **Yes** | **Yes** | **Yes** | **Yes** | **Yes** |  |  |
| **Q15. If they performed quantitative synthesis did the review authors carry out an adequate investigation of publication bias (small study bias) and discuss its likely impact on the results of the review?** | **No**  The "Assessment of reporting biases" section stated that funnel plots were not used due to the limited number of studies (<10 per analysis). | **Yes** | Not applicable – No meta-analysis was conducted. | **No**  No graphical or statistical tests (e.g., funnel plot) were conducted. | Not applicable – No meta-analysis was conducted. | **Yes** | Not applicable – No meta-analysis was conducted. | **Yes** | **No**  Publication bias was not assessed. | | **No**  Publication bias was not assessed. | **No**  Publication bias was not assessed. | **Yes** | Not applicable – No meta-analysis was conducted. | **No**  Publication bias was not assessed. | **No**  Publication bias was not assessed. | **No**  Publication bias was not assessed. | Not applicable – No meta-analysis was conducted. |  |  |
| **Q16. Did the review authors report any potential sources of conflict of interest, including any funding they received for conducting the review?** | **Yes** | **Yes** | **Yes** | **Yes** | **Yes** | **Yes** | **Yes** | **Yes** | **Yes** | | **Yes** | **Yes** | **Yes** | **Yes** | **Yes** | **Yes** | **Yes** | **Yes** |  |  |
| **Overall quality** | **LQ** | **LQ** | **LQ** | **CLQ** | **LQ** | **LQ** | **MQ** | **LQ** | **CLQ** | | **CLQ** | **CLQ** | **LQ** | **MQ** | **CLQ** | **CLQ** | **CLQ** | **CLQ** |  |  |
| **PAI**: Physical Adjunctive Interventions; **BES**: Bioelectric stimulation; **VDs**: Vibration devices; **PBMD**: Photobiomodulation; **MQ**: Moderate quality; **LQ:** Low quality; **CLQ**: Critical low quality. | | | | | | | | | | | | | | | | | | | |  |
