# Supplementary material for: The Effectiveness of Physical Adjunctive Interventions in the Acceleration of Orthodontic Tooth Movement: An Umbrella Review and Meta‐Analysis
Source: Int J Dent. 2026 Feb 3;2026:9131541. doi: 10.1155/ijod/9131541 (PMC12868923; doi:10.1155/ijod/9131541)
Supplement: Supplementary file 11 — Supporting Information 11 Table S11: Detailed supporting reasons for the ROBIS Tool assessment of each included systematic review. [file IJOD-2026-9131541-s013.docx]

| **Supplementary Table 11:** Detailed supporting reasons for the ROBIS Tool assessment of each included systematic review | | | | | | | | | | | | | | | | | | | | | | | | | | | | | | | | | | | | | | | |
| --- | --- | --- | --- | --- | --- | --- | --- | --- | --- | --- | --- | --- | --- | --- | --- | --- | --- | --- | --- | --- | --- | --- | --- | --- | --- | --- | --- | --- | --- | --- | --- | --- | --- | --- | --- | --- | --- | --- | --- |
|  | **Domain 1** | | | | | | | **Domain 2** | | | | | | | | | | **Domain 3** | | | | | | | **Domain 4** | | | | | | | | | | **ROB** | | | | |
|  | **1.1** | **1.2** | **1.3** | **1.4** | **1.5** | **Risk** | **2.1** | | **2.2** | | **2.3** | | **2.4** | | **2.5** | **Risk** | **3.1** | | **3.2** | **3.3** | **3.4** | **3.5** | **Risk** | **4.1** | | **4.2** | | **4.3** | | **4.4** | **4.5** | **4.6** | **Risk** | **a** | | **b** | **c** | **Risk** | **Rationale** |
| El-Angbawi, 2023 | Y | Y | PY | Y | Y | Low | Y | | Y | | PY | | Y | | Y | low | Y | | Y | PY | PY | Y | low | Y | | PN | | Y | | Y | PN | Y | Low | Y | | Y | Y | Low | Comprehensive search, dual independent processes, and transparent reporting.  GRADE framework used to contextualize evidence certainty.  Conclusions prioritized clinical relevance and acknowledged limitations of primary studies.  Minor gaps in addressing ambiguities (Domain 1) and incomplete subgroup analyses (Domain 4). |
|  | The eligibility criteria were clear and suitable for the research question, with strict adherence to predefined standards. Minor ambiguity in details (e.g., defining "other anomalies") does not significantly weaken the process's credibility. | | | | |  | The review employed rigorous methods, using multiple databases and independent dual screening for reliability. The only minor limitation—incomplete search strategy details—does not substantially affect its quality. | | | | | | | | |  | ROB was assessed using a validated standardized tool (ROB 1 + GRADE). Two reviewers independently performed data extraction and bias assessments, and key study details/results were documented. | | | | | |  | Despite some limitations (e.g., incomplete reporting of preplanned analyses), the analysis appropriately addressed variance and bias. Results consistently highlighted the limited effectiveness of non-surgical interventions, supporting overall credibility. | | | | | | | | |  |  |  |  |  |  |  |
| Dutta, 2024 | Y | Y | PY | PN | Y | low | PY | | Y | | PY | | PY | | Y | low | Y | | Y | PN | Y | Y | Low | PN | | Y | | PY | | Y | PN | PN | High | PN | | Y | PN | High | Critical flaws in synthesis (Domain 4) due to high heterogeneity, exclusion of studies, and unaddressed biases in primary studies.  Partial mitigation of concerns in other domains (e.g., language restrictions, selection bias).  Conclusions rely heavily on limited, potentially biased evidence, reducing confidence in robustness. |
|  | Eligibility criteria were largely predefined, appropriate, and unambiguous. The primary concern is the lack of clarity on language restrictions, which could affect comprehensiveness. | | | | |  | The review applied rigorous methods (dual screening, hand-searching) with minor limitations (incomplete grey literature inclusion and unspecified language restrictions) yet maintained low concerns by adhering to systematic review standards. | | | | | | | | |  | ROB was assessed using a validated standardized tool (ROB 2 + GRADE). Two reviewers independently performed data extraction and bias assessments, and key study details/results were documented. | | | | | |  | Exclusion of eligible studies from synthesis, high heterogeneity in the 5th-month analysis, and failure to address biases in primary studies reduce confidence in the reliability of findings. | | | | | | | | |  |  |  |  |  |  |  |
| Aljabaa, 2018 | PN | Y | PY | Y | Y | Unclear | PY | | Y | | Y | | Y | | Y | Low | Y | | Y | Y | Y | Y | low | Y | | Y | | Y | | PN | PN | Y | High | PN | | Y | PY | High | Despite low concerns in Domains 1–3, the high concern in Domain 4 (unaddressed heterogeneity, lack of robustness checks, and potential bias from industry-sponsored studies) critically undermines the validity of the conclusions. |
|  | No registered protocol  The eligibility criteria were mostly pre-specified, appropriate, and unambiguous. The primary concern is the lack of clarity on restrictions related to information sources (e.g., language/publication bias), which is insufficiently documented. | | | | |  | The review applied rigorous methods (dual screening, hand-searching) but had minor limitations (e.g., incomplete grey literature). Concerns remain low due to adherence to systematic review standards. | | | | | | | | |  | ROB was assessed using a validated standardized tool (ROB 1). Two reviewers independently performed data extraction and bias assessments, and key study details/results were documented. | | | | | |  | While the narrative synthesis was appropriate given heterogeneity, the lack of quantitative methods to address variation (e.g., subgroup analyses) and assess robustness (e.g., sensitivity analyses) raises concerns. Additionally, high heterogeneity and potential bias in primary studies (e.g., industry sponsorship) may undermine the validity of conclusions. | | | | | | | | |  |  |  |  |  |  |  |
| Abd Elmotaleb, 2019 | Y | Y | Y | Y | N | High | PN | | Y | | PY | | PN | | Y | High | Y | | Y | PN | Y | Y | Low | Y | | PY | | PY | | PN | PN | Y | High | PN | | Y | Y | High | Domain 2 and 4 Concerns Dominate: The high risk of publication bias (due to excluded non-English/unpublished studies) and weak synthesis (limited meta-analysis, no sensitivity analyses) critically undermine the review’s reliability.  Incomplete Mitigation: The authors did not adequately discuss how these biases might affect conclusions, reducing confidence in the findings. |
|  | Although pre-registered protocols demonstrate rigor, unjustified language restrictions (English-only) and unassessed exclusion bias introduce validity-threatening restrictions. | | | | |  | Limited database and supplementary search methods coverage, and language restrictions raise concerns about missed studies | | | | | | | | |  | ROB was assessed using a validated standardized tool (ROB 1). Two reviewers independently performed data extraction and bias assessments, and key study details/results were documented. | | | | | |  | Limited studies (only 3) reduce statistical power and reliability.  No sensitivity/suppression analyses restrict bias evaluation and result consistency.  Unaddressed variability (e.g., measurement methods) risks misleading conclusions. | | | | | | | | |  |  |  |  |  |  |  |
| Bakdach, 2020 | PN | Y | PY | Y | Y | Unclear | Y | | Y | | Y | | Y | | Y | low | Y | | Y | PY | PY | Y | low | Y | | PY | | PY | | PN | N | PN | High | PN | | Y | Y | High | Rationale: No registered protocol  The inability to conduct meta-analyses, insufficient handling of biases in primary studies, and addressing heterogeneity (Domain 4) raises concerns about the validity of conclusions |
|  | Rationale: No registered protocol  Minor ambiguity in outcome definitions, but overall eligibility criteria were clear and relevant | | | | |  | A comprehensive, language-unrestricted search with dual screening reduces selection bias. | | | | | | | | |  | ROB was assessed using a validated standardized tool (ROB 1). Two reviewers independently performed data extraction and bias assessments, and key study details/results were documented. | | | | | |  | Rationale: Lack of quantitative synthesis and unaddressed heterogeneity weaken conclusions. | | | | | | | | |  |  |  |  |  |  |  |
| Keerthana, 2020 | Y | Y | Y | Y | N | High | PN | | Y | | Y | | PN | | Y | High | Y | | Y | PY | PY | Y | Low | PN | | Y | PY | | Y | | PY | PY | Unclear | PN | | Y | Y | High | Selection bias in study inclusion (language restrictions and lack of comprehensive database coverage).  Weak assessment and analysis of sources of heterogeneity and bias in the included studies.  Failure to discuss how these limitations affect the credibility of the conclusions. |
|  | Although pre-registered protocols demonstrate rigor, unjustified language restrictions (English-only) and unassessed exclusion bias introduce validity-threatening restrictions. | | | | |  | Limited database coverage and language restrictions raise concerns about missed studies | | | | | | | | |  | ROB was assessed using a validated standardized tool (ROB 1 + GRADE). Two reviewers independently performed data extraction and bias assessments, and key study details/results were documented. | | | | | |  | Robust synthesis methods addressed heterogeneity and bias, though some exclusions reduced comprehensiveness | | | | | | | | |  |  |  |  |  |  |  |
| García Vega, 2021 | Y | Y | PY | N | N | High | PY | | PN | | Y | | N | | Y | High | Y | | Y | PY | PY | Y | Low | PY | | Y | PN | | N | | N | PY | High | PN | | PN | PY | High | Language and Date Restrictions: Excluding non-English studies and those published before 2010 may omit critical evidence.  2. Incomplete Search Strategy: Failure to search clinical trial registries or grey literature (e.g., unpublished reports).  3. Failure to Address Between-Study Variation: The significant diversity in devices and measurement methods was not statistically addressed, weakening conclusions.  4. Absence of Sensitivity Analyses: No analyses were conducted to assess the robustness of findings or investigate publication bias. |
|  | Although pre-registered protocols demonstrate rigor, Unjustified date restrictions (2010–2021), unjustified language restrictions (English-only), and unassessed exclusion bias introduce validity-threatening restrictions. | | | | |  | Language bias and incomplete search strategies (e.g., no gray literature) likely omitted critical evidence. | | | | | | | | |  | ROB was assessed using a validated standardized tool (ROB 1 + GRADE). Two reviewers independently performed data extraction and bias assessments, and key study details/results were documented. | | | | | |  | Failure to address heterogeneity and assess robustness weakens confidence in conclusions. | | | | | | | | |  |  |  |  |  |  |  |
| Dutta, 2025 | Y | Y | PY | Y | Y | Low | Y | | Y | | PY | | Y | | Y | Low | Y | | Y | Y | Y | Y | Low | Y | | Y | PY | | PY | | Y | Y | Unclear | PY | | Y | Y | Unclear | The review demonstrated strong methodology in Domains 1–3 and mitigated some concerns in Domain 4 through sensitivity analyses and publication bias checks. However, unexplained heterogeneity remains a key limitation, reducing confidence in the synthesis. While not "high risk," the conclusions require cautious interpretation due to variability in study outcomes. |
|  | Pre-registration and structured PICOS criteria minimized bias, though minor ambiguities (e.g., "local factors") reduced clarity. | | | | |  | Rigorous screening minimized selection bias, but incomplete search strategy details reduced reproducibility. | | | | | | | | |  | ROB was assessed using a validated standardized tool (ROB 2 + GRADE). Two reviewers independently performed data extraction and bias assessments, and key study details/results were documented. | | | | | |  | While the authors addressed publication bias, performed sensitivity analyses, and discussed primary study biases, the extreme heterogeneity (I² = 96%) remains a critical limitation. The lack of subgroup analyses to explore heterogeneity sources (e.g., device type, frequency, patient compliance) reduces confidence in the pooled estimates. | | | | | | | | |  |  |  |  |  |  |  |
| de Almeida, 2016 | Y | Y | PN | N | Y | High | Y | | PN | | PY | | Y | | PY | Unclear | PY | | Y | PN | PY | Y | High | Y | | PY | Y | | PN | | PN | PN | High | PN | | Y | Y | High | The study faced methodological limitations, including unclear exclusion criteria, reliance on a non-validated assessment tool (modified Cericato checklist), alongside a failure to address heterogeneity across studies and evaluate the robustness of findings without conducting sensitivity analyses. |
|  | Unclear exclusion criteria (e.g., first premolar extraction focus) lacked sufficient justification.  Limited search strategies | | | | |  | The study followed rigorous research procedures, but a lack of specific search strategy details and supplementary search methods raised uncertainties. | | | | | | | | |  | Use of a non-validated quality tool and incomplete outcome collection limit reliability | | | | | |  | Failure to address heterogeneity among studies and the absence of sensitivity analyses undermine the credibility of the findings. | | | | | | | | |  |  |  |  |  |  |  |
| Imani, 2018 | PN | Y | PY | Y | Y | Unclear | Y | | PN | | PY | | Y | | Y | Unclear | Y | | Y | Y | Y | Y | Low | Y | | PY | PN | | PN | | PN | Y | High | N | | Y | Y | High | Lack of a registered review protocol, no manual search, no grey literature search, unaddressed heterogeneity, synthesis limitations, no sensitivity analysis, and publication bias |
|  | No registered protocol  Eligibility criteria were well-defined and aligned with the research question | | | | |  | Comprehensive database searches and dual screening reduce bias, though limited by neglecting manual searches and grey literature. | | | | | | | | |  | ROB was assessed using a validated standardized tool (ROB 1). Two reviewers independently performed data extraction and bias assessments, and key study details/results were documented. | | | | | |  | High heterogeneity and lack of exploration into its sources, coupled with no sensitivity analyses, raise concerns about result reliability | | | | | | | | |  |  |  |  |  |  |  |
| Deana, 2019 | PN | Y | PN | Y | N | Unclear | Y | | PN | | PN | | N | | Y | High | Y | | PN | Y | Y | Y | Unclear | N | | PY | PN | | PN | | N | N | High | N | | PN | Y | High | Multiple high-concern domains (eligibility, selection, synthesis) and failure to address limitations in interpretation. |
|  | Lack of protocol registration and ambiguous criteria (e.g., incomplete laser parameters) raised concerns. Language restrictions introduced potential selection bias. | | | | |  | Language restrictions, lack of supplementary search methods, and insufficiently detailed search terms likely missed relevant studies. | | | | | | | | |  | The evaluation was systematic, but the lack of detail in some studies affected the interpretation. | | | | | |  | Unexplained exclusion of studies, unaddressed heterogeneity, and lack of robustness checks undermined validity. | | | | | | | | |  |  |  |  |  |  |  |
| Bakdach, 2020 | PN | Y | PY | PY | Y | Unclear | Y | | Y | | Y | | Y | | Y | Low | Y | | Y | Y | Y | Y | Low | Y | | Y | PY | | Y | | PY | Y | Unclear | Y | | Y | Y | Unclear | The study demonstrates transparent reporting, acknowledgment of limitations, and rigorous methodology; however, the absence of a registered protocol and sensitivity testing of the results lowers the evaluation |
|  | Eligibility criteria were clearly defined in advance and aligned with the research goals. No registered protocol was available. Minor ambiguities (e.g., age ranges) did not significantly impact the study’s credibility. | | | | |  | A comprehensive, language-unrestricted search with dual screening reduces selection bias. | | | | | | | | |  | ROB was assessed using a validated standardized tool (ROB 1 + GRADE). Two reviewers independently performed data extraction and bias assessments, and key study details/results were documented. | | | | | |  | Appropriate methods addressed heterogeneity and bias; however, caution is warranted due to clinical diversity and potential publication bias, with no sensitivity analyses conducted | | | | | | | | |  |  |  |  |  |  |  |
| Camacho, 2020 | PY | Y | PY | N | N | High | Y | | PY | | PN | | N | | Y | High | Y | | Y | PY | PY | Y | Low | Y | | Y | PN | | N | | PN | PY | High | N | | PY | Y | High | Language bias – Potential exclusion of key non-English studies.  Unaddressed heterogeneity – Variations in laser parameters/metrics not analyzed.  Limited robustness – No sensitivity analyses or publication bias assessments. |
|  | Although pre-registered protocols demonstrate rigor, Unjustified date restrictions (2001–2018), unjustified language restrictions (English-only), and unassessed exclusion bias introduce validity-threatening restrictions. | | | | |  | Language restrictions, lack of supplementary search methods, and incomplete reporting of the search strategy increased the likelihood of missing eligible studies | | | | | | | | |  | ROB was assessed using a validated standardized tool (ROB 1). Two reviewers independently performed data extraction and bias assessments, and key study details/results were documented. | | | | | |  | Poor handling of heterogeneity and a lack of sensitivity analyses weakened conclusions | | | | | | | | |  |  |  |  |  |  |  |
| Grajales, 2023 | Y | Y | Y | Y | Y | Low | Y | | | Y | PY | Y | | Y | | Low | Y | | Y | Y | Y | Y | Low | PY | | PY | Y | | PN | | N | PN | High | PN | | Y | Y | High | Significant unexplained heterogeneity across studies.  No sensitivity analyses or publication bias assessment.  Results unadjusted for potential bias risks in primary studies. |
|  | Criteria are clearly predefined with logically justified constraints. | | | | |  | Implement a systematic approach to enhance decision accuracy in selection processes. | | | | | | | | |  | ROB was assessed using a validated standardized tool (ROB 2). Two reviewers independently performed data extraction and bias assessments, and key study details/results were documented. | | | | | |  | Unexplained high variance and the absence of sensitivity analyses undermine the credibility of the findings. | | | | | | | | |  |  |  |  |  |  |  |
| Jnaneshwar, 2023 | Y | Y | PY | Y | PY | Low | Y | | | PY | PY | Y | | Y | | Low | Y | | Y | Y | Y | Y | Low | Y | | PY | Y | | Y | | PY | Y | Low | Y | | Y | Y | Low | The review adhered to rigorous standards, following a registered protocol with no language restrictions. Comprehensive search and analysis methods were employed, with appropriate handling of bias and variability. The findings are supported by robust, clear evidence |
|  | The criteria are clear and appropriate, the review protocol is pre-registered, and there are no language restrictions | | | | |  | Adopt a structured methodology to improve precision in selection decision-making | | | | | | | | |  | ROB was assessed using a validated standardized tool (ROB 2 + GRADE). Two reviewers independently performed data extraction and bias assessments, and key study details/results were documented. | | | | | |  | The review adhered to rigorous synthesis protocols, and the absence of sensitivity analyses was due to the high homogeneity among studies. | | | | | | | | |  |  |  |  |  |  |  |
| Malik, 2024 | PN | Y | PY | Y | N | High | Y | | | N | PN | N | | PY | | High | PY | | Y | Y | PY | PN | Low | PY | | N | PN | | N | | N | PN | High | N | | PY | Y | High | Major concerns in Domains 1, 2, and 4, coupled with incomplete addressing of limitations |
|  | Restricting to English-only studies and a lack of a pre-registered protocol raise concerns about selection bias. | | | | |  | Language restrictions, lack of supplementary search methods, and incomplete reporting of search strategy increase the risk of missing studies | | | | | | | | |  | ROB was assessed using a validated standardized tool (ROB 1). Two reviewers independently performed data extraction and bias assessments, and key study details/results were documented. | | | | | |  | High heterogeneity, lack of exploration for bias sources, and incomplete reporting undermine confidence | | | | | | | | |  |  |  |  |  |  |  |
| Hmida, 2024 | PN | Y | PY | Y | N | High | Y | | | Y | PN | N | | Y | | High | Y | | Y | Y | Y | Y | Low | PN | | PY | Y | | PN | | N | PN | High | PN | | Y | Y | High | Potential language-related publication bias.  Opaque study exclusion criteria.  Unaddressed statistical heterogeneity across studies.  Absence of sensitivity analyses to confirm result robustness. |
|  | Restricting to English-only studies and a lack of a pre-registered protocol raise concerns about selection bias. | | | | |  | Language restrictions and low search strategy sensitivity may lead to the exclusion of critical studies. | | | | | | | | |  | ROB was assessed using a validated standardized tool (ROB 2 + GRADE). Two reviewers independently performed data extraction and bias assessments, and key study details/results were documented. | | | | | |  | unjustified study exclusions, lack of sensitivity analyses, and inadequate handling of statistical heterogeneity. | | | | | | | | |  |  |  |  |  |  |  |
| **ROB**: risk of bias; **Y**: Yes; **PY**: Probably Yes; **PN**: Probably No; **N**: No; **NI**: No Information | | | | | | | | | | | | | | | | | | | | | | | | | | | | | | | | | | | | | | | |
