# Supplementary material for: The Effectiveness of Physical Adjunctive Interventions in the Acceleration of Orthodontic Tooth Movement: An Umbrella Review and Meta‐Analysis
Source: Int J Dent. 2026 Feb 3;2026:9131541. doi: 10.1155/ijod/9131541 (PMC12868923; doi:10.1155/ijod/9131541)
Supplement: Supplementary file 12 — Supporting Information 12 Table S12: The need for reassessment of risk of bias in primary studies using ROB2 tool. [file IJOD-2026-9131541-s012.docx]

| **Supplementary Table 12:** The Need for Reassessment of Risk of Bias in Primary Studies Using ROB2 Tool | | | | |
| --- | --- | --- | --- | --- |
| **SRs /**  **Author, Year, Country** | **ROB Assessment Tool** | **AMSTAR-2 tool assessments** | **ROBIS** | **Judgments** |
| **El-Angbawi et al, 2023, UK** | **ROB1** | **LQ** | **L** | Reassessment ROB |
| **Dutta et al., 2024, India** | **ROB2** | **LQ** | **H** | Reassessment ROB |
| **Aljabaa et al. 2018, Saudi Arabia/USA** | **ROB1** | **LQ** | **H** | Reassessment ROB |
| **Abd Elmotaleb et al., 2019, Egypt** | **ROB1** | **CLQ** | **H** | Reassessment ROB |
| **Bakdach et al. 2020, Syria** | **ROB1** | **LQ** | **H** | Reassessment ROB |
| **Keerthana et al. 2020, India** | **ROB1** | **LQ** | **H** | Reassessment ROB |
| **García Vega et al.2021, Mexico** | **ROB1** | **MQ** | **H** | Reassessment ROB |
| **Dutta et al. 2025, India** | **ROB2** | **LQ** | **Unclear** | Reassessment ROB |
| **De Almeida et al., 2016, Brazil** | **Selection criteria and scores, adapted from Cericato et al** | **CLQ** | **H** | Reassessment ROB |
| **Imani et al. 2018, Iran** | **ROB1** | **CLQ** | **H** | Reassessment ROB |
| **Deana et al. 2019, Chile** | **ROB1** | **CLQ** | **H** | Reassessment ROB |
| **Bakdach et al. 2020, Syria** | **ROB1** | **LQ** | **Unclear** | Reassessment ROB |
| **Camacho et al.2020, Colombia** | **ROB1** | **MQ** | **H** | Reassessment ROB |
| **Grajales et al. 2023, Spain** | **ROB2** | **CLQ** | **H** | Reassessment ROB |
| **Jnaneshwar et al. 2023, India** | **ROB1** | **CLQ** | **L** | Reassessment ROB |
| **Malik et al. 2024, India** | **ROB1** | **CLQ** | **H** | Reassessment ROB |
| **Hmida et al. 2024, Tunisia** | **ROB2** | **CLQ** | **H** | Reassessment ROB |
| **SRs**: Systematic reviews; **ROBIS**: risk of bias in systematic reviews; **AMSTAR**: A Measurement Tool to Assess Systematic Reviews; **ROB1**: use of the ROB1 tool for studies in 2019 or earlier; **ROB1**: use of the ROB1 tool for studies after 2019; **ROB2**: Risk of Bias 2; **MQ**: Moderate Quality; **LQ**: Low Quality; **CLQ**: Critical Low Quality; **L**: Low Risk; **H**: High Risk.  **Primary studies included in systematic reviews are re-assessed with ROB2 if any of these apply:**   - An unvalidated tool was originally used - ROB1 was applied to studies post-2019 - AMSTAR-2 rates the review as low/critically low quality - ROBIS finds a high risk of bias | | | | |
