# Supplementary material for: The Effectiveness of Physical Adjunctive Interventions in the Acceleration of Orthodontic Tooth Movement: An Umbrella Review and Meta‐Analysis
Source: Int J Dent. 2026 Feb 3;2026:9131541. doi: 10.1155/ijod/9131541 (PMC12868923; doi:10.1155/ijod/9131541)
Supplement: Supplementary file 13 — Supporting Information 13 Table S13: Risk‐of‐bias reassessment of included RCTs with supporting justifications. [file IJOD-2026-9131541-s011.docx]

| **Supplementary Table 13:** Risk-of-Bias Reassessment of Included RCTs with Supporting Justifications | | | | | | | | |
| --- | --- | --- | --- | --- | --- | --- | --- | --- |
| **Study** | | **Randomization process** | **Deviations from intended interventions** | **Missing outcome data** | **Measurement of the outcome** | **Selection of the reported result** | **Overall bias** |  |
| Accelerating Orthodontic Treatment Using VDs | **Alansari, 2017** | **Low** **risk**. Random sequence generation and allocation concealment were clearly described. | **Some concerns:** Sham devices were used for blinding, but true blinding may not have been fully effective. | **Some concerns:** Drop-out handling unclear | **High risk:** Assessors measured tracking & cytokines unblinded | **Some concerns:** The protocol was not mentioned; there is a potential selective reporting concern. | **High risk:**  The study is judged to raise high risk because one of the domains got this result |  |
|  | **Azeem et al, 2019** | **Some concerns:** Random sequence generation is described, but no mention of allocation concealment. | **Low** **risk.** The intervention was implemented as intended, and patients adhered well. | **Low** **risk.** Outcome data were complete with no reported dropout. | **Low** **risk** Outcomes (tooth movement and pain) were measured with validated tools by blinded assessors. | **Some concerns:** No trial registration or protocol is cited; potential for selective reporting remains. | **Some concerns:**  The study is judged to raise some concerns risk because two of the domains got this result |  |
|  | **Bisht, 2019** | **Some concerns:** Randomization is mentioned, but there is no information on allocation concealment. | **Low** **risk**. Blinding of participants was attempted with placebo devices; however, awareness of the dummy device was noted. | **Low** **risk**. No dropouts or missing data were reported across groups. | **Some concerns:** Pain and alignment outcomes were measured objectively using VAS and Little’s index. Assessor blinding not stated. | **Some concerns:** No mention of protocol registration; potential for selective outcome reporting exists. | **Some concerns:**  The study is judged to raise some concerns risk because three of the domains got this result |  |
|  | **Bragassa, 2018** | **Some concerns:** Randomization is stated, but the method and allocation concealment are not clearly described. | **Low** **risk**. The intervention (vibration) was well implemented; adherence was monitored. | **Low** **risk**. No significant loss to follow-up; outcome data were complete and balanced. | **Low** **risk**. Outcomes (pain and tooth movement) were measured using validated tools like VAS and 3D scans. | **Some concerns:** No mention of protocol registration or pre-specification of outcomes; selective reporting is possible. | **Some concerns:**  The study is judged to raise some concerns risk because two of the domains got this result |  |
|  | **Bulic, 2017** | **Low** **risk**. – Random straw‑draw assignment; sequence concealed; baseline demographics (age, gender) well balanced. | **Some concerns:** – Participants unblinded; no sham device, so behavior or reporting (especially pain) might have been influenced. | **Low** **risk**. – Dropouts and missing pain data documented; exclusions applied equally; no evidence of bias due to differential missingness. | **Some concerns:** – Alignment measured in blinded digital models (low risk); pain was self‑reported without blinding, introducing possible measurement bias. | **Some concerns:** – No trial registration or prespecified analysis; multiple pain endpoints create risk for selective outcome reporting. | **Some concerns:**  The study is judged to raise some concerns risk because three of the domains got this result |  |
|  | **Chouinard, 2016** | **Low** **risk**. Computer-generated sequence with central randomization. | **Low** **risk**. Blinding and adherence ensured; ITT analysis conducted. | **Some concerns:** Small attrition; handling unclear | **Low** **risk**. Blinded assessors for biomarker analysis. | **Low** **risk**. Outcomes pre-specified; all results reported. | **Some concerns:**  The study is judged to raise some concerns risk because one of the domains got this result |  |
|  | **Dibiase et al, 2018** | **Low** **risk**. Appropriate randomization and allocation concealment are described. | **Low** **risk**. Well-controlled with blinding and compliance monitored. | **Low** **risk**. Minimal loss to follow-up with analysis per protocol. | **Low** **risk**. Objective measures and blinded outcome assessors. | **Low** **risk**. The protocol was available and adhered to. | **Low** **risk:**  The study is judged to be at low risk of bias for all domains. |  |
|  | **Gujar et al, 2023** | **Some concerns:** Randomization method mentioned, but concealment unclear. | **Some concerns:** No blinding; potential deviation due to self-administered intervention. | **Low** **risk**. Adequate data reported with minimal loss. | **Some concerns:** Movement gauged by the operator; assessor blinding not stated. | **Low** **risk**. No protocol available; all key outcomes reported. | **Some concerns:**  The study is judged to raise some concerns risk because three of the domains got this result |  |
|  | **Kalemaj et al, 2017** | **Some concerns:** Randomization process described but lacks concealment details. | **Some concerns:** Adherence was monitored, but blinding was not clearly reported. | **Low** **risk**. Complete data with no attrition reported. | **Some concerns:** IL-1β & alignment measured, assessor blinding not stated | **Low** **risk**. Reported as per pre-defined protocol. | **Some concerns:**  The study is judged to raise some concerns risk because three of the domains got this result |  |
|  | **Kannan et al, 2019** | **Some concerns:** Split-mouth, allocation method unclear | **Low** **risk**. The protocol was adhered to with minimal risk of deviation. | **Low** **risk**. Minimal missing data; handled appropriately. | **Some concerns:** Calliper measurements by a treating clinician. Assessor blinding not stated | **Low** **risk**. Reported outcomes match protocol intentions. | **Some concerns:**  The study is judged to raise some concerns risk because two of the domains got this result |  |
|  | **Katchooi et al, 2018** | **Low** **risk**. Randomized with computer sequence and central allocation. | **Low** **risk**. Adherence monitored; blinding applied appropriately. | **Low** **risk**. Low attrition with balanced follow-up. | **Low** **risk**. Blinded assessors used for outcome evaluation. | **Low** **risk**. Pre-specified outcomes reported per protocol. | **Low** **risk:**  The study is judged to be at low risk of bias for all domains. |  |
|  | **Khera et al, 2022** | **Low** **risk**. Random sequence generation and allocation concealment were appropriate. | **Low** **risk**. Participants and personnel were blinded; no deviation reported. | **Low** **risk**. Minimal attrition; ITT analysis used. | **Some concerns:** Outcomes assessors were blinded, though not clearly detailed. | **Low** **risk**. All outcomes pre-specified; selective reporting unlikely. | **Some concerns:**  The study is judged to raise some concerns risk because one of the domains got this result |  |
|  | **Kumar et al, 2020** | **Low** **risk**. Randomization method and allocation concealment are clearly described. | **Some concerns:** Participants and operator un-blinded; only data analyst blinded – protocol identical otherwise | **Low** **risk**. All participants accounted for, with low dropout. | **Low** **risk**. Blinding of outcome measurement is described clearly. | **Low** **risk**. Results consistent with pre-specified outcomes. | **Some concerns:**  The study is judged to raise some concerns risk because one of the domains got this result |  |
|  | **Leethanakul et al, 2016** | **Low** **risk**. Split-mouth randomization; assignment done randomly. | **Low** **risk**. Adherence and delivery of the intervention were consistent. | **Low** **risk**. No missing data or significant dropout reported. | **Low** **risk**. Outcomes were measured with validated methods by blinded investigators. | **Low** **risk**. Reporting matches protocol and objectives. | **Low** **risk:**  The study is judged to be at low risk of bias for all domains. |  |
|  | **Liao et al, 2017** | **Low** **risk**. Computer-generated randomization with split-mouth design. | **Low** **risk**. No deviation from intended interventions. | **Low** **risk**. Low attrition and complete outcome data. | **Some concerns:** Measurements intra-orally and on casts; assessor blinding not stated. | **Some concerns:** Comprehensive results, but a lack of registration. | **Some concerns:**  The study is judged to raise some concerns risk because two of the domains got this result |  |
|  | **Lombardo et al, 2019** | **Low** **risk**. Computer-randomization and allocation concealment are described. | **Low** **risk**. Participants and assessors were blinded; the protocol was followed closely. | **Low** **risk**. No dropout or missing data. | **Low** **risk**. Objective outcomes are assessed with digital modeling tools. | **Low** **risk**. The trial was registered and analyzed according to pre-specified outcomes. | **Low** **risk:**  The study is judged to be at low risk of bias for all domains. |  |
|  | **Mayama et al, 2022** | **Low** **risk**. Computer-generated sequence concealed in sequential opaque envelopes; baseline characteristics comparable. | **Low** **risk**. Split-mouth design and uniform operator minimized departures; any lack of participant blinding is unlikely to influence orthodontic vibration’s objective effects. | **Low** **risk**. < 5 % attrition; reasons unrelated to outcome; analyzed on an ITT basis. | **Low** **risk**. Root-resorption quantified by blinded assessor using micro-CT—objective, standardized method. | **Low** **risk**. Prospectively registered (UMIN000013722); manuscript reports all prespecified outcomes and time points. | **Low** **risk:**  The study is judged to be at low risk of bias for all domains. |  |
|  | **Miles & Fisher, 2016** | **Low** **risk**. Permuted blocks of 10 were produced in Excel, and assignments were kept in sequential opaque envelopes; the operator and model-assessor were blinded, so allocation concealment is adequate. | **Low** **risk**. Participants knew their group, but care was delivered per protocol, and primary outcomes were objective arch measurements; no evidence of systematic deviations. | **Low** **risk**. All 40 randomized adolescents completed every time-point; the CONSORT flow chart shows zero attrition or missing measures. | **Low** **risk**. Primary outcomes were taken from plaster models by a blinded assessor; objective tools minimize detection bias (VAS pain was secondary). | **Some concerns:** The article states, “Registration: This trial was not registered. Protocol was not published before trial commencement,” raising potential selective-reporting concerns. | **Some concerns:**  The study is judged to raise some concerns risk because one of the domains got this result |  |
|  | **Miles et al, 2012** | **Some concerns:** Block randomization in blocks of six is described, but no information on who generated the sequence or how allocation was concealed. | **Low** **risk**. Participants knew their group, yet care pathways were identical and the clinician delivering treatment was blinded; outcomes are objective, so deviations unlikely. | **Low** **risk**. CONSORT diagram shows only 2/66 (3 %) withdrawals for irregularity and 1/60 (2 %) for pain, evenly balanced between arms. | **Low** **risk**. Irregularity measured on plaster models by a blinded assessor; VAS pain secondary. | **Some concerns:** The article contains no trial registration or pre-published protocol, so selective reporting cannot be ruled out . | **Some concerns:**  The study is judged to raise some concerns risk because two of the domains got this result |  |
|  | **Miles et al, 2018** | **Low** **risk**. Permuted blocks of 10 were generated in Excel and concealed in sequential opaque envelopes; clinical assistants opened envelopes after bonding, keeping the operator blind — adequate sequence generation + concealment. | **Low** **risk**. Single-blind design: participants knew allocation but operator and model-assessor were blinded; primary outcome (millimeters of space closure) is objective, and analysis followed the randomized groups ⇒ deviations unlikely to bias effect. | **Low** **risk**. All 40 randomized adolescents completed every time-point; no drop-outs or missing impressions reported. | **Low** **risk**. Space closure measured on plaster models by a blinded assessor; ICC 0.99–0.998 shows excellent reliability, low detection bias. | **Some concerns:** Authors state “This trial was not registered. Protocol was not published before trial commencement,” so selective outcome reporting cannot be ruled out. | **Some concerns:**  The study is judged to raise some concerns risk because one of the domains got this result |  |
|  | **Pavlin et al, 2015** | **Low** **risk**. Third-party, computer-generated block randomization (size = 4) with stratification; allocation key kept off-site, ensuring concealment and well-balanced baseline groups. | **Low** **risk**. Parallel, double-blind design with an inert sham device; investigators, clinicians, and participants masked, and analysis followed the ITT principle—no meaningful protocol deviations. | **Low** **risk**. Intention-to-Treat ITT included all 45 randomized participants; only 6 were excluded from per-protocol for non-outcome-related reasons (< 5 %), so bias from missing data is unlikely. | **Low** **risk**. Tooth movement was measured monthly with digital calipers by two independent, blinded raters; excellent reliability (ICC 0.98) indicates objective, precise assessment. | **Some concerns:** Trial pre-registered on ClinicalTrials.gov and FDA IDE, industry funding, so some concern for selective reporting | **Some concerns:**  The study is judged to raise some concerns risk because one of the domains got this result |  |
|  | **Pescheret, 2017** | **Some concerns:** Allocation was by “drawing straws”, with no description of sequence generation or concealment → possible predictability and selection bias. Baseline balance was acceptable, but the method remains sub-optimal. | **Some concerns:** Participants knew their assignment and aligner-change intervals differed (7 vs 14 days); compliance with the vibrating device averaged 77 %. Treating clinicians were blinded, limiting performance bias, but non-adherence could still affect the effects. | **Some concerns:** 4/40 (10 %) participants withdrew (3 experimental, 1 control). Attrition was balanced and reasons reported, yet some pain-survey data were missing; no imputation was described. | **Some concerns:** Alignment was measured on de-identified digital scans (assessor masking, objective metric). Pain was self-reported and unblinded, but this was secondary. Overall, minor detection bias is possible. | **High risk:** No protocol or trial registration found; multiple outcomes analyzed but only selected ones reported (e.g., rate of alignment, pain). Selective reporting cannot be ruled out. | **High risk:**  The study is judged to raise high risk because one of the domains got this result |  |
|  | **Reiss et al, 2020** | **Low** **risk**. Block randomization stratified by sex; allocations in sequential opaque envelopes opened after bonding—adequate sequence generation and concealment. | **Some concerns:** Participants knew whether they received AcceleDent, and average compliance was only 53 %; analysis was ITT, clinicians/outcome assessors were masked, but unblinded subjects + variable adherence give some concerns. | **Some concerns:** 3/40 (7.5 %) withdrawals, all in control; several saliva samples missing with no imputation—loss is small but unbalanced and could influence pilot-size estimates. | **Low** **risk**. Irregularity measured on models by a blinded evaluator; biomarkers analyzed in a central lab—objective methods, low detection bias. | **Some concerns:** Trial registered on ClinicalTrials.gov (NCT02119455) before start, but no public protocol and multiple biomarkers explored; selective reporting can’t be excluded. | **Some concerns:**  The study is judged to raise some concerns risk because three of the domains got this result |  |
|  | **Siriphan et al, 2019** | **Some concerns:** Allocation by card-shuffling gave a random sequence, but concealment procedures were not described | **Some concerns:** Participants and operators knew who received vibration; compliance reminders were sent, but not logged. Analysis was ITT for tooth-movement, yet behavior could differ between arms | **Low** **risk**. Primary outcome (3-month canine distalization) included all 60 randomized subjects; cytokine analyses lost 8 (balanced across groups) but were secondary | **Low** **risk**. Tooth movement was measured on blinded, superimposed digital models; cytokines were quantified in a central lab. Objective, assessor-masked | **Some concerns:** Trial was registered (TCTR20170707004) but no publicly available protocol/analysis plan; exploratory biomarkers added post-hoc cannot be ruled out | **Some concerns:**  The study is judged to raise some concerns risk because three of the domains got this result |  |
|  | **Taha et al, 2019** | **Some concerns:** Simple random sequence generated in GraphPad; no details on allocation concealment (e.g., sealed envelopes), so predictability can’t be ruled out | **Some concerns:** Participants knew if they received AcceleDent, and average compliance fell to ≈56 %; no sham device; clinicians adjusted forces equally, but differential behavior can’t be excluded | **Low** **risk**. All 21 adolescents were analyzed at every time-point; no withdrawals or missing scans/pain scores reported → low risk | **Some concerns:** Canine movement measured on 3D super-imposed scans (objective), yet by the same, unmasked investigator; pain self-reported and unblinded. Objective metric limits bias, but assessor awareness remains | **Some concerns:** Trial described as IRB-approved pilot, but no protocol or registry number disclosed; multiple secondary outcomes explored (pain, compliance) with selective emphasis possible | **Some concerns:**  The study is judged to raise some concerns risk because four of the domains got this result |  |
|  | **Telatar et al, 2020** | **Some concerns:** Allocation was by simple coin-tossing with no description of sequence concealment—predictability and foreknowledge can’t be excluded | **Some concerns:** Participants used (or not) AcceleDent®; no sham device, and compliance differed (mean 85 %, higher in females). Operators knew assignment; analysis was ITT, but behavioral differences could influence effect | **Low** **risk**. 1/20 (5 %) dropout for non-study reasons; attrition is small and balanced enough to pose low risk. | **Some concerns:** Canine movement measured on 3-D digital scans; objective metric, yet the same investigator who randomized also measured and was unblinded—minor detection bias | **Some concerns:** Trial registered (NCT04206267) but no publicly available protocol/analysis plan; exploratory subgroup analyses reported selectively | **Some concerns:**  The study is judged to raise some concerns risk because four of the domains got this result |  |
|  | **Woodhouse et al, 2015** | **Low** **risk**. Computer-generated sequence; central allocation independent of clinicians ensured concealment | **Low** **risk**. Sham device controls expectations; operators & patients masked to active vs sham. | **Low** **risk**. 4/81 (5 %) withdrawals (balanced across arms); reasons unrelated to outcomes and no important missing cast data | **Low** **risk**. Mandibular alignment measured on coded casts by a blinded assessor with excellent intra-rater ICC (0.95) | **Low** **risk**. Trial prospectively registered (ClinicalTrials.gov NCT02314975); primary and secondary outcomes reported as planned | **Low** **risk:**  The study is judged to be at low risk of bias for all domains. |  |
|  | **Yildiz et al, 2023** | **Some concerns:** Described as a split-mouth, single-blind RCT with patients “randomly divided into two groups”, but no details on sequence generation or allocation concealment. | **Some concerns:** Participants knew whether they used AcceleDent and two Hycon activation schedules differed; clinicians also knew, yet analysis was ITT, and outcomes were objective. | **Low** **risk**. All 20 adolescents completed distalization; no drop-outs or missing 3-D scans/pain charts reported → low risk. | **Some concerns:** Canine movement quantified on digitized casts with 3-Shape software; assessor masking not stated, so minor detection bias possible. | **Some concerns:** Trial registered (NCT03968263) but no public protocol/analysis plan; several exploratory subgroup analyses (sex, side) reported. | **Some concerns:**  The study is judged to raise some concerns risk because four of the domains got this result |  |
| Accelerating Orthodontic Treatment Using PBM | **Abellán et al, 2021** | **Some concerns:** Allocation list created with Research Randomizer but the paper does not state who prepared it or how envelopes/assignments were concealed; baseline groups appear balanced. | **Some concerns:** Patients and operator knew whether photobiomodulation (PBM) was delivered; the PBM group attended extra laser sessions while controls did not. All 20 patients finished and were analyzed (near-ITT), yet open-label care could influence behaviour . | **Low** **risk**. 0 % attrition: every participant contributed complete scanner and CBCT records at all planned time-points. | **High risk:** Intrusion distance taken from super-imposed STL models/CBCT; assessor blinding is not reported. Digital measurements are objective, but potential awareness of group status leaves High risks. | **Some concerns:** Trial registered only in an internal university database; no public protocol or prospective registry and secondary outcomes (root-resorption volume, periodontal indices) may have been post-hoc. | **High risk:**  The study is judged to raise high risk because one of the domains got this result |  |
|  | **Alam, 2019** | **Some concerns:** The study used a parallel design and stated patients were randomly allocated into four groups using G*Power software to calculate the sample size, but did not describe how the random sequence was generated or concealed. | **High risk:** Neither the patients nor the operator were blinded to intervention, and the same operator performed both LLLT and non-LLLT, introducing potential performance bias. | **Low** **risk**. All 32 patients completed the study with no dropouts. | **Low** **risk**. Pain was measured via self-reported NRS with telephone follow-ups, reducing detection bias concerns. | **Some concerns:** There was no mention of trial registration or a pre-specified statistical analysis plan, leading to some concerns for selective reporting. | **High risk:**  The study is judged to raise high risk because one of the domains got this result |  |
|  | **AlSayed Hasan et al, 2017** | **Some concerns:** Allocation by patients drawing folded slips marked “laser” or “control”. No description of who prepared slips or whether identical envelopes were used | **Some concerns:** Participants and operator knew whether low-level-laser therapy (LLLT) was delivered; no sham device. Follow-up schedule and wire sequence were identical and analysis was ITT, yet awareness could influence behaviour (e.g., appointment enthusiasm) | **Low** **risk**. All 26 randomized patients completed every measurement time-point; 0 % attrition. | **Low** **risk**. Alignment was assessed on study casts with Little’s Irregularity Index by the same examiner; high intra-class reliability (ICC = 0.998) and objective metric mitigate detection bias. | **Low** **risk**. Trial prospectively registered (ClinicalTrials.gov NCT02568436) and primary/secondary outcomes reported as planned; selective reporting unlikely. | **Some concerns:**  The study is judged to raise some concerns risk because two of the domains got this result |  |
|  | Al-Shaf et al., 2021 | **Low** **risk**. Random sequence generation and allocation concealment were clearly described (computer randomization & opaque envelopes). | **Some concerns:** Patients and clinicians could not be blinded due to obvious sham design, although outcomes unlikely to be affected; thus "some concerns". | **Low** **risk**. Minimal missing data due to COVID-19; addressed properly with statistical models, impact judged minimal. | **Low** **risk**. Outcome assessors were blinded by deidentifying digital scans; measurements objective. | **Low** **risk**. No evidence of selective reporting; outcomes and analysis methods were pre-defined. | **Some concerns:**  The study is judged to raise some concerns risk because one of the domains got this result |  |
|  | Arumughan et al, 2018 | **Some concerns:** Split-mouth: the experimental side was “randomly selected by an individual who is not part of the study” but the article does not describe sequence generation (e.g., random-number table) or concealment. | **Some concerns:** Patients and operator knew which side received 810 nm diode-laser LLLT; no sham irradiation. Force application (150 g coil springs) was identical, yet awareness could influence cooperation or measurement timing. | **Low** **risk**. All 12 participants contributed measurements at baseline and day 84; no withdrawals or exclusions reported. | **Some concerns:** Canine–premolar distance taken with a digital calliper (±0.001 mm) by the same (unblinded) examiner; objective but assessor awareness may affect repeat readings. | **Some concerns:** The trial was not prospectively registered and only the primary outcome (space closure rate) is reported; pain/adverse events are mentioned in discussion of other studies but not presented. | **Some concerns:**  The study is judged to raise some concerns risk because four of the domains got this result |  |
|  | **Caccianiga et al, 2016** | **Some concerns:** Block randomization via computer was used, but allocation concealment was not described. | **Some concerns:** Participants and clinicians knew who received low-level-laser therapy; no sham device was used and the laser group attended extra appointments every second week. Although aligner-wear instructions were identical (12 h/day), unequal visit frequency and lack of blinding could influence co-interventions or enthusiasm | **Low** **risk**. All 21 patients completed follow-ups (Fig. 1). Outcomes (aligner fit/treatment success) were reported for all participants. | **High risk:** Subjective outcome: "Correct aligner fit" was assessed by an unblinded orthodontist, introducing detection bias.  No blinding: The orthodontist knew group assignments and may have been influenced (e.g., stricter assessment for controls).  Outcome definition: Lack of objective criteria for "passive fit" (e.g., digital tracking). | **Some concerns:** No protocol/pre-registration: No mention of a pre-published analysis plan.  Selective reporting: Only the aligner fit/treatment success was reported. Pain, root resorption, or bone turnover (mentioned in the Introduction) were not analyzed.  Incomplete results: Secondary outcomes (e.g., rotational movement) were measured but not compared between groups. | **High risk:**  The study is judged to raise high risk because one of the domains got this result |  |
|  | **Caccianiga et al, 2017** | **Low** **risk**. Computer-generated sequence (SPSS) with study numbers concealed until bonding—adequate sequence generation and allocation concealment. | **Some concerns:** Patients and the single operator knew who received LLLT; no sham device. Treatment visits followed a fixed 4-week schedule and analysis was ITT, but knowledge of group could influence care or behaviour (e.g., scheduling eagerness). | **Low** **risk**. All 36 randomized participants completed alignment; no withdrawals or missing time-to-event data reported. | **Some concerns:** Alignment end-point judged visually by the same (unblinded) operator; although based on objective bracket engagement, assessor awareness could bias the “crowding resolved” decision. | **Some concerns:** Trial described as ethics-approved “pilot” but not prospectively registered; only primary outcome (days to alignment) and one secondary (number of visits) reported—other time-dependent or safety outcomes may have been explored but unreported. | **Some concerns:**  The study is judged to raise some concerns risk because three of the domains got this result |  |
|  | **Cruz et al., 2004** | **Some concerns:** The study did not clearly describe the method of random allocation to sides (left vs right) for laser vs control, raising concerns about potential allocation bias. | **Low** **risk**. No major deviations were described. Both groups received intended interventions as per protocol. | **Low** **risk**. No dropouts reported; data was complete for all 11 patients. | **Some concerns:** Measurements were performed in-office using a caliper, but no mention of blinding of the assessor, leading to possible detection bias. | **Some concerns:** The study did not provide a pre-registered protocol or mention selective outcome reporting, creating some uncertainty. | **Some concerns:**  The study is judged to raise some concerns risk because three of the domains got this result |  |
|  | **Dalaie et al, 2015** | **Some concerns:** The study reported using complete block randomization via Microsoft Excel, but did not provide details on sequence generation security or allocation concealment. | **Low** **risk**. Both patients and evaluators were blinded to side allocation. The laser vs. control sides were clearly distinguished by an independent person handling the device, minimizing deviations. | **Low** **risk**. No missing data: all 12 patients completed the study without dropouts. | **Low** **risk**. Tooth movement was measured on dental casts with a digital caliper (Mitutoyo, 0.01 mm) by blinded assessors, reducing measurement bias. | **Some concerns:** There was no mention of trial registration or pre-specified analysis plan, leading to some concerns for selective reporting. | **Some concerns:**  The study is judged to raise some concerns risk because two of the domains got this result |  |
|  | **Domínguez et al, 2015** | **High risk:** The study reported random selection of patients for enrollment, but then always assigned the right side to laser and left side to placebo, without random allocation of sides, leading to high risk of selection bias. | **Low** **risk**. A placebo procedure was performed on the control side by placing the laser tip without activation, minimizing performance bias, thus low risk. | **Low** **risk**. All 10 patients completed the study with no missing data, so low risk. | **Low** **risk**. Outcomes (3D scanned tooth movement, ELISA for RANKL/OPG, and VAS pain) were measured objectively under the same protocol, reducing detection bias, thus low risk. | **Some concerns:** No protocol registration or pre-specified analysis plan was reported, raising some concerns for selective reporting. | **High risk:**  The study is judged to raise high risk because one of the domains got this result |  |
|  | **Doshi-Mehta et al, 2012** | **Some concerns:** Allocation was described only as a “randomly assigned incomplete-block split-mouth design.” No details on sequence generation or allocation concealment | **Some concerns:** Patients were blinded to the test/control quadrant, but the clinician applying the laser could not be blinded; no indication that this influenced co-interventions. | **Low** **risk**. All 20 enrolled participants completed follow-up; no exclusions reported. | **Low** **risk**. Tooth movement was measured with a digital caliper by an assessor who was explicitly stated to be blinded, and pain was patient-reported with a validated VAS; instruments are objective/validated. | **Some concerns:** The methods section lists distance and pain as outcomes and both are fully reported; however, no protocol or pre-registration is referenced, so selective reporting cannot be ruled out. | **Some concerns:**  The study is judged to raise some concerns risk because three of the domains got this result |  |
|  | **Ekizer et al, 2016** | **Low** **risk**. The study used proper randomization (coin toss), allocation concealment via sealed envelopes, and balanced groups with split-mouth design, minimizing selection bias. | **Low** **risk**. Blinding of both patients (device on both sides, but active only on one) and clinicians measuring outcomes was clearly reported, reducing performance and detection bias. | **Low** **risk**. No missing data was reported; 4 excluded patients were explained a priori for non-compliance, and complete data analysis was performed on the remaining 20 subjects. | **Low** **risk**. Outcomes were measured using validated tools (3D model superimpositions for tooth movement, RFA for miniscrew stability, ELISA for IL-1β) by blinded assessors. | **Some concerns:** Although most outcomes were pre-specified, there was limited discussion on a pre-registered protocol or statistical analysis plan, introducing some concern about selective reporting. | **Some concerns:**  The study is judged to raise some concerns risk because one of the domains got this result |  |
|  | El Shehawy et al, 2020 | **Some concerns:** Simple computer-generated sequence (GraphPad) was used, but the article does not describe allocation concealment (e.g., sealed-opaque envelopes); predictability cannot be ruled out. | **Some concerns:** Participants and operators knew whether photodynamic therapy was delivered; no sham device. Visits and arch-wire sequence were identical across arms and analyses were ITT, yet knowledge of group could influence behaviour. | **Some concerns:** 4/30 (13 %) withdrew (2 per arm) for missed laser appointments; remaining 26 analyzed with no imputation plan stated. Attrition is moderate but balanced. | **Low** **risk**. Mandibular alignment measured on blinded 3-D digital models with software; objective metric, good intra-rater ICC (0.96-0.99). | **Low** **risk**. Trial prospectively registered (NCT04376164) and primary/secondary outcomes reported as planned; selective reporting unlikely. | **Some concerns:**  The study is judged to raise some concerns risk because three of the domains got this result |  |
|  | **Farhadian et al, 2021** | **Low** **risk**. Stratified block randomization (by sex & bracket slot) prepared by an independent person; allocations placed in sequentially numbered opaque envelopes → adequate sequence generation + concealment. | **Some concerns:** Participants knew whether they used LED daily or attended laser sessions; controls received a sham “coated light-cure” exposure, and clinicians followed identical retraction mechanics. Analysis was per-protocol (4/60 exclusions) rather than ITT, so non-adherence could slightly bias effects. | **Low** **risk**. 56/60 (93 %) participants provided complete monthly models; attrition (6.7 %) was small and reasons unrelated to outcome (lost LED device, missed laser visits). | **Low** **risk**. 3-D canine movement measured on anonymized scans; assessor and statistician were blinded; objective digital measurements with ICC = 0.97. | **Some concerns:** Trial was retrospectively registered (IRCT20120220009086N4) after enrolment began and no public protocol/statistical plan is available; secondary outcomes (pain, rotation) reported but selective emphasis can’t be excluded. | **Some concerns:**  The study is judged to raise some concerns risk because two of the domains got this result |  |
|  | **Ghaffar et al, 2022** | **Low** **risk**. Computer-generated sequence; assignments placed in sealed opaque envelopes and kept by an independent secretary → adequate sequence generation & concealment. | **Some concerns:** Patients/operator knew group (laser vs none) and no sham; both groups attended the same recall schedule, and primary outcome (days to alignment) is unlikely to be affected by expectations. Still, knowledge could influence self-reported pain. | **Low** **risk**. 2/32 (6 %) withdrawals (1 per arm) with stated, unrelated reasons; digital models available for the remaining 30 patients. | **Low** **risk**. Alignment measured on blinded 3-Shape digital models (ICC 0.83-0.92); objective metric. Pain recorded by unblinded patients, but this is a secondary outcome. | **Some concerns:** Study reports ethics approval but no prospective trial registration or published protocol; multiple time-point outcomes analyzed, so selective reporting cannot be ruled out. | **Some concerns:**  The study is judged to raise some concerns risk because two of the domains got this result |  |
|  | **Guram et al, 2018** | **Some concerns:** Authors state “randomized double-blind split-mouth… a third evaluator divided quadrants” and that allocation was concealed from participants and first evaluator. However, the method of sequence generation (e.g., random-number table, computer list) and details of concealment (sealed envelopes, etc.) were not described | **Low** **risk**. Split-mouth design; both quadrants treated identically except for laser; participants and outcome assessors masked; no protocol deviations reported | **Low** **risk**. All 20 randomized participants completed the study; no exclusions or attrition mentioned; outcomes available for all time-points | **Low** **risk**. Tooth movement measured on cast models with digital caliper by blinded assessor; objective, validated method; identical timing across arms | **Some concerns:** No trial registration or pre-published protocol; multiple outcomes/time-points reported, but possibility of selective emphasis cannot be ruled out. | **Some concerns:**  The study is judged to raise some concerns risk because two of the domains got this result |  |
|  | **Hasan et al. 2022** | **Low** **risk.** Random sequence generation and allocation concealment were adequately reported (simple randomization with sealed envelopes). | **Low** **risk,** no deviations; interventions were applied as intended, and compliance was monitored monthly. | **Low** **risk:** No missing data reported; all participants completed the study as per CONSORT flow. | **Low** **risk** Standardized cephalometric measures with high intra-rater reliability (ICCs ≥0.97) and paired t-test showed no systematic error. | **Low** **risk** Outcomes were pre-specified (registered at ANZCTR) and all planned outcomes were reported. | **Low** **risk:**  The study is judged to be at low risk of bias for all domains. |  |
|  | **Heravi, 2014** | **Some concerns:** The paper does not explicitly describe how random allocation to laser vs placebo sides was performed, raising some concern. | **Low** **risk,** the intervention and placebo procedures were well controlled and equally administered on both sides. | **Low** **risk,** no missing data was reported, and all patients completed the study. | **Some concerns:** Blinding was partial: the operator knew the allocation, though patients were blinded, and measurement methods (TIP, models) were prone to some detection bias. | **Low** **risk,** Outcomes were reported as per protocol, including all pre-specified measures. | **Some concerns:**  The study is judged to raise some concerns risk because two of the domains got this result |  |
|  | **Hosseini, 2011** | **Low** **risk,** Randomization process was adequately described using a split-mouth design with random side allocation, reducing selection bias. | **Low** **risk,** there was no indication of deviations from intended interventions; both sides followed the planned protocols strictly. | **Low** **risk,** all patients completed the protocol except one excluded appropriately; missing data was minimal and handled transparently. | **Low** **risk,** Blinding of the measurement process was done via casts and reference plaques; objective measures minimized detection bias. | **Low** **risk,** no selective reporting was evident; outcomes were reported as pre-specified with appropriate statistical tests. | **Low** **risk:**  The study is judged to be at low risk of bias for all domains. |  |
|  | **Impellizzeri et al., 2020** | **Some concerns:** Some concern - Randomization was done using randomizer.org, but details on allocation concealment and baseline comparability are limited. | **Low** **risk,** Low risk - Split-mouth design with careful control of interventions and operator consistency. | **Low** **risk,** Low risk - No missing data; all patients completed planned protocol. | **Some concerns:** Some concern - Measurements were manual on models, single assessor, blinding not fully detailed. | **Some concerns:** Some concern - As a pilot study, selective reporting risk exists due to limited predefined outcomes. | **Some concerns:**  The study is judged to raise some concerns risk because three of the domains got this result |  |
|  | **Isola et al, 2019** | **Low** **risk,** Computer-generated 1:1 allocation, sealed opaque envelopes; allocation by an independent clinician | **Some concerns:** Participants not blinded and split-mouth design; objective outcome (tooth movement) unlikely affected, but subjective VAS pain could be influenced | **Low** **risk,** all 41 patients completed follow-up; no exclusions, no imputation | **Some concerns:** Tooth movement measured by unblinded examiner; pain self-reported by non-blinded patients; potential detection bias. | **Some concerns:** Trial protocol/registration not reported; selective reporting can’t be ruled out. | **Some concerns:**  The study is judged to raise some concerns risk because three of the domains got this result |  |
|  | **Kansal et al, 2014** | **Some concerns:** The study stated random allocation of quadrants to laser vs. control using coin toss, but did not provide allocation concealment details. | **Low** **risk**. The interventions were consistently applied with same appliances and standardized forces, and blinding of operator measuring distances was reported. | **Low** **risk**. There was no missing data; all 10 patients completed the study with clear reporting of outcomes. | **Some concerns:** The measurements were performed by a blinded operator, but the outcome assessment relied on simple caliper readings without calibration details or repeated measures reliability. | **Some concerns:** The outcomes reported matched the study objectives, but lack of protocol registration raises uncertainty about selective reporting. | **Some concerns:**  The study is judged to raise some concerns risk because three of the domains got this result |  |
|  | **Kau et al, 2013** | **Some concerns:** Subjects were said to be “randomized into groups with varying exposure times,” yet the article gives no details on sequence generation or allocation concealment; test/control numbers are highly unbalanced (73 vs 17) | **Some concerns:** Participants and clinicians knew who used the PBM device; no sham, and exposure schedules differed (daily vs weekly vs none). Analysis was largely ITT, but open-label use could alter co-interventions or motivation | **Low** **risk**. All 90 enrolled subjects appear in the mixed-effects analysis; the paper reports no withdrawals and handles outliers transparently. | **Some concerns:** Little’s Irregularity Index measured every 2–4 weeks by calibrated site examiners, but blinding of assessors to group is not stated; awareness could influence analogue calliper readings. | **Some concerns:** Trial was industry-sponsored, not prospectively registered, and only alignment speed and visits were reported; other planned outcomes (e.g., pain, safety) are not presented | **Some concerns:**  The study is judged to raise some concerns risk because four of the domains got this result |  |
|  | **Kharat et al, 2023** | **Some concerns:** The paper states that “the right- and left-extracted quadrants were randomly categorized into two groups,” but gives no details on sequence generation or allocation concealment. Operator blinding is also unclear. | **Some concerns:** Participants were blinded to the irradiated side, yet the operator who delivered the laser could not be blinded; no information on adherence checks or protocol deviations. However, no co-interventions likely. | **Low** **risk** All 20 enrolled patients were followed to the final assessment; no drop-outs or exclusions reported. | **Some concerns:** Tooth-movement was measured with a vernier caliper (objective) but by a non-blinded operator; pain scores were self-reported and participants could guess their allocation after a few days | **Some concerns:** No trial registry or pre-published protocol; multiple outcomes reported without clarity on primary vs. secondary and no mention of selective analysis decisions. | **Some concerns:**  The study is judged to raise some concerns risk because four of the domains got this result |  |
|  | **Kochar et al, 2017** | **Some concerns:** Authors state “randomly selected split-mouth design,” but do not describe the random-sequence generation method or allocation-concealment procedures. Baseline similarity was not statistically demonstrated. | **Low** **risk** Split-mouth design; both sides treated identically except for active laser; participants were blinded to the lased side; no protocol deviations reported. | **Low** **risk** All 20 enrolled participants completed the study; no exclusions or losses reported; analyses were performed on all randomized units. | **Low** **risk** Tooth movement measured on casts with a digital caliper by a blinded assessor; pain recorded with VAS; participants were unaware of group allocation. Outcomes are objective or self-reported but unlikely to be differentially biased. | **Some concerns:** No registered protocol is cited; outcome measures and time points reported appear reasonable, but selective-reporting cannot be ruled out (e.g., no variance data for some secondary outcomes, no mention of adverse events). | **Some concerns:**  The study is judged to raise some concerns risk because two of the domains got this result |  |
|  | **Lalnunpuii et al, 2020** | **Some concerns:** Computer-generated allocation sequence is described, and baseline groups were comparable. However, concealment is insufficiently detailed (“sequences were concealed and were chosen by the patient”), so protection against fore-knowledge of assignments is uncertain. | **Some concerns:** Due to the nature of the intervention, patients and primary investigator could not be blinded, which may have affected subjective outcomes (though primary outcome was objective), hence some concerns. | **Low** **risk** the authors report that all 65 randomized participants completed treatment and were included in the analysis; no attrition or exclusions are mentioned. | **Low** **risk** Outcome was measured with digital models to 0.001 mm accuracy; outcome assessor (data-analyst) was blinded; the measurement method is valid and identical across groups. | **Some concerns:** Trial was prospectively registered (CTRI/2018/04/013156), but the publicly available record does not contain a detailed statistical-analysis plan or a list of secondary outcomes. Because the protocol was “not published before trial commencement”, it is unclear whether unreported analyses exist | **Some concerns:**  The study is judged to raise some concerns risk because three of the domains got this result |  |
|  | **Limpanichkul et al, 2006** | **Some concerns:** The study used block randomization to assign sides to LLLT vs placebo, but did not clearly describe how the sequence was generated or how allocation concealment was ensured | **Low** **risk**. The operator used opaque and black probe sheaths with a third person managing device allocation, ensuring the operator was blinded to intervention, minimizing performance bias. | **Low** **risk**. All 12 patients completed the trial with no dropouts | **Low** **risk**. Tooth movement was measured using high-precision stereomicroscopy with a palatal plug reference device, by the same operator | **Some concerns:** The study did not mention pre-registration of a study protocol or pre-specified statistical analysis plan | **Some concerns:**  The study is judged to raise some concerns risk because two of the domains got this result |  |
|  | **Lo Giudice et al, 2020** | **Low** **risk**. Balanced-block list (stratified by sex & crowding) created in SPSS; assignments sealed in sequential opaque envelopes and opened at bonding → adequate sequence generation & concealment | **Some concerns:** Participants received in-office PBM sessions every 14 days; controls had no sham. Operator knew allocation, but scheduling, arch-wire sequence, and ITT analysis were identical across arms; behaviour could still differ | **Some concerns:** 11/100 (11 %) randomized subjects excluded after allocation (2 discontinued, 9 scheduling non-compliance); final sample 46 vs 43. Attrition moderate and reasons partly related to intervention | **Low** **risk**. Time-to-alignment decided by an assessor “unaware of whether subjects were PBM or control”; outcome is objective (complete contact correction) | **Some concerns:** Ethics approval reported, but no prospective registration or public protocol; only two outcomes (days & visits) presented—risk of unreported analyses | **Some concerns:**  The study is judged to raise some concerns risk because three of the domains got this result |  |
|  | **Mal et al. (2018)** | **Some concerns:** The study mentions random selection of quadrants for split-mouth design but does not specify the method of random sequence generation or allocation concealment. | **Low** **risk**. There is no indication of deviations from intended interventions; laser and control sides were managed as per protocol. | **Low** **risk**. All patients completed the study, with no missing data reported. | **Some concerns:** The study does not describe if outcome assessors were blinded, introducing uncertainty in measurement bias. | **Low** **risk**. All pre-specified outcomes appear to be reported with appropriate statistical analyses. | **Some concerns:**  The study is judged to raise some concerns risk because two of the domains got this result |  |
|  | **Mistry et al, 2020** | **Low** **risk**. Random sequence generation and allocation concealment were clearly reported using www.randomisation.com and sealed envelopes, with a split-mouth design limiting confounding. | **Low** **risk**. Triple-blind study (patients, operators, and statisticians) with identical sham controls minimizes risk of deviations. | **Low** **risk**. Only one patient excluded due to appliance breakage, fully reported, unlikely to bias results. | **Low** **risk**. Outcomes measured digitally by blinded operator with high intra-examiner reliability (CCC >0.98); objective measures like space closure, rotation, anchorage. | **Low** **risk**. Trial registered (ACTRN12619001237178); no evidence of selective reporting; all prespecified outcomes reported. | **Low** **risk:**  The study is judged to be at low risk of bias for all domains. |  |
|  | **Nahas et al, 2016** | **Some concerns:** Allocation by sealed envelopes drawn by patients; article does not describe who generated the sequence or how envelopes were prepared | **High risk:** Two test-arm participants were excluded post-randomization (≤ 80 % compliance, device failure); four controls were also dropped. Excluding non-adherent subjects directly ties to the intervention effect | **Some concerns:** 6/40 (15 %) withdrawals (4 control, 2 test) for reasons potentially linked to outcome; no imputation plan reported | **Some concerns:** Primary end-point (“days to decrowding”) decided by treating clinician who was aware of group, but Little’s index measured later on coded casts by a blinded assessor | **Some concerns:** Trial not prospectively registered and no public protocol; only selected outcomes (time, crowding index) reported, so selective reporting cannot be ruled out | **High risk:**  The study is judged to raise high risk because one of the domains got this result |  |
|  | **Okla et al, 2018** | **Some concerns:** Described as a “prospective, randomized, double-blind clinical trial,” but the article does not specify how the random sequence was generated or whether allocation was concealed with opaque envelopes → some concerns. | **Some concerns:** A sham (non-emitting) device was supplied, yet the test units were wireless while controls were wired, making blinding of participants and clinicians uncertain; compliance issues (missed sessions, device replacement) were managed pragmatically, but could differentially influence outcomes → some concerns. | **High risk:** 38 subjects randomised, only 26 (68 %) analysed at 6 months; seven “non-compliant” and five “missed-visit” exclusions occurred after randomization. Attrition was substantial and imbalanced (exact allocation not reported) with no imputation or sensitivity analysis → high risk. | **Some concerns:** Irregularity Index measured on stone casts with digital callipers; root length measured from calibrated periapicals. Examiner reliability was reported, but no statement of assessor blinding. Objective metrics limit bias, yet unblinded assessment cannot be excluded → some concerns. | **Some concerns:** The paper gives ethics approval but no prospective trial registration or publicly available protocol; multiple secondary outcomes (e.g., compliance, root resorption) analyzed without pre-specification, so selective reporting cannot be ruled out → some concerns. | **High risk:**  The study is judged to raise high risk because one of the domains got this result |  |
|  | **Pereira, 2014** | **Some concerns:** There is limited information about how the randomization sequence was generated or concealed. This raises some concerns about potential allocation bias. | **Low** **risk**. No notable deviations from the intended interventions were reported. All participants appear to have received the allocated treatment as planned. | **Low** **risk**. The study did not report substantial loss to follow-up, and outcomes were analyzed for nearly all participants | **Low** **risk**. Outcomes were objective (pain scores and functional parameters) and assessed similarly across groups, with no indication of differential measurement | **Some concerns:** The publication lacks a pre-registered protocol, and selective reporting cannot be ruled out (e.g., no mention if all planned outcomes were reported) | **Some concerns:**  The study is judged to raise some concerns risk because two of the domains got this result |  |
|  | **Pérignon et al, 2021** | **Low** **risk**. Used secure computerized block randomization via an independent unit (IWRS), with allocation concealed from recruiters | **Some concerns:** Patients and operator could not be blinded due to the laser procedure nature, although placebo with inactive laser was applied and same sounds were used | **Low** **risk**. Only 1 patient excluded pre-treatment due to missing consent; all others completed study. | **Low** **risk**. Outcomes (distance by caliper, rate of movement, VAS pain) were measured by an independent blinded examiner, reducing detection bias | **Some concerns:** Although trial was registered (NCT02181439), the analysis plan and protocol deviations (like imbalance in baseline Class II severity) were not detailed | **Some concerns:**  The study is judged to raise some concerns risk because two of the domains got this result |  |
|  | **Qamruddin et al., 2017** | **Low** **risk**. The study was a randomized split-mouth clinical trial using coin flip allocation, with well-balanced baseline characteristics. | **Low** **risk**. Patients and assessors were blinded to the laser intervention (placebo side with inactive laser), minimizing deviations. | **Low** **risk**. Complete data were reported with only two dropouts due to clear reasons (spring dislodgement, analgesic use), unlikely to bias results. | **Low** **risk**. Outcomes were measured using objective digital models for tooth movement and validated VAS pain scales with structured patient instructions and blinding, minimizing detection bias. | **Low** **risk**. All prespecified outcomes (canine movement and pain) were reported as planned. | **Low** **risk:**  The study is judged to be at low risk of bias for all domains. |  |
|  | **Samara et al, 2018** | **Some concerns:** Allocation used sealed envelopes drawn by patients in two blocks of 30; the paper does not state who prepared the sequence or whether envelopes were opaque and sequentially numbered | **Some concerns:** Participants knew they were using the daily LED device; no sham was provided. Analysis of the primary outcome was ITT, but compliance < 80 % led to exclusion in the per-protocol analysis and the PBM group had extra appliance use | **Some concerns:** 15 / 60 (25 %) patients dropped out (compliance failure, relocations, appliance breakage). The authors used multiple imputation, yet high attrition and reasons related to the intervention keep risk at some concerns | **Low** **risk**. Premolar-space widths were measured on coded plaster casts by a blinded examiner with ICC = 0.999; objective metric gives low risk | **Some concerns:** Trial and protocol were not prospectively registered; only velocity and visit data were reported, leaving room for selective non-reporting of other outcomes (e.g., pain, harms) | **Some concerns:**  The study is judged to raise some concerns risk because four of the domains got this result |  |
|  | **Sandoval et al, 2017** | **Some concerns:** Split-mouth design with quadrants “randomly divided” to laser vs control, but the article gives no details on sequence generation or allocation concealment | **Some concerns:** Patients were told both quadrants would receive hand-piece contact (guiding light used on control side), yet the operator knew which quadrant was irradiated; clinical care identical otherwise. Operator awareness creates potential but limited performance bias | **Low** **risk**. All 20 participants contributed data from both quadrants at every time-point; no withdrawals or exclusions reported | **Low** **risk**. In-mouth canine–premolar distances were taken with a digital calliper by an assessor blinded to which side was lasered; objective metric with Dahlberg error ≤ 0.05 mm | **Some concerns:** Trial was not prospectively registered and only velocity/space-closure outcomes are presented; other potential outcomes (pain, adverse effects) absent | **Some concerns:**  The study is judged to raise some concerns risk because three of the domains got this result |  |
|  | **Sousa et al, 2011** | **Some concerns:** The study used a split-mouth design and stated that one canine side was randomly chosen for laser application. However, it did not describe the method used to generate or conceal the random allocation sequence | **Low** **risk**. Patients were blinded to which side received laser; treatments adhered to the protocol, minimizing risk. | **Low** **risk**. No missing data reported; all patients completed the study and were analyzed. | **Low** **risk**. Outcome assessment was blinded and used precise 3D scans and radiographs, reducing detection bias. | **Some concerns:** The study did not mention a pre-specified protocol or statistical analysis plan, raising some concerns for selective reporting. | **Some concerns:**  The study is judged to raise some concerns risk because two of the domains got this result |  |
|  | **Souza, 2014** | **Some concerns:**  the thesis did not clearly describe the randomization method or allocation concealment; only stated selection of canines (split-mouth). | **Low** **risk**. there was no indication of deviations from intended interventions; the laser vs. control sides were treated per protocol. | **Low** **risk**. the study followed up all included participants with consistent measures. | **Some concerns:** measurement relied on cast scanning and VAS by patients without blinding assessors. | **Some concerns:** selective reporting could not be excluded due to incomplete protocol registration. | **Some concerns:**  The study is judged to raise some concerns risk because three of the domains got this result |  |
|  | **Üretürk et al, 2017** | **Some concerns:** The study used a split-mouth design (right vs left canine) but did not clearly describe the sequence generation or allocation concealment methods | **Low** **risk**. As patients served as their own controls and treatments were applied strictly per protocol by the same operator, deviations from intended interventions are unlikely. | **Low** **risk**. All enrolled patients completed the study with no indication of missing outcome data. | **Low** **risk**. Outcomes were measured objectively using 3D digital models (3Shape) and ELISA assays for cytokines, reducing detection bias. | **Some concerns:** The protocol or pre-specified analysis plan was not reported, so selective reporting cannot be ruled out. | **Some concerns:**  The study is judged to raise some concerns risk because two of the domains got this result |  |
|  | **Varella et al, 2018** | **Some concerns:** The study used a split-mouth design and assigned experimental/control sides by a lottery with sealed envelopes before subject recruitment. However, it did not describe how the random sequence was generated or if allocation concealment was robust. | **Low** **risk**. The study ensured blinding of the operator by using opaque and clear sheaths on the laser probe and having a third person handle the laser device. Therefore, deviations were unlikely | **Low** **risk**. All 10 patients completed the study with no dropouts, minimizing attrition bias. | **Low** **risk**. The outcomes (canine retraction and IL-1β levels) were assessed using validated measures (occlusograms, digital software, ELISA) by blinded operators, minimizing detection bias. | **Some concerns:** There was no mention of pre-registration of a protocol or pre-specified analysis plan, introducing some concern for selective reporting. | **Some concerns:**  The study is judged to raise some concerns risk because two of the domains got this result |  |
|  | **Yassaei et al, 2016** | **Low** **risk**. The study used a randomized split-mouth double-blind design with clear random allocation of quadrants for laser vs control. | **Low** **risk**. No deviations noted; laser intervention was consistently applied on the randomly assigned side, with a pseudo-laser procedure on the control side to maintain blinding. | **Low** **risk**. No dropouts, loss to follow-up, or missing data were reported in the study; complete data were analyzed. | **Low** **risk**. Outcome measurements (tooth movement via casts & calipers; IL-6 levels via ELISA) were appropriate and identical across groups, blinded to intervention side. | **Low** **risk**. The study reported all predefined outcomes (velocity of tooth movement, IL-6 levels) with no signs of selective reporting. | **Low** **risk:**  The study is judged to be at low risk of bias for all domains. |  |
|  | **Youssef et al, 2008** | **Some concerns:** The authors state that the right side of each jaw received laser while the left served as control, but no description of a true random sequence or allocation concealment is provided. | **Low** **risk**. Split-mouth design ensured participants and clinicians could not switch sides after allocation, and no co-interventions were reported. Compliance was adequate. | **Low** **risk**. All 15 participants completed the 6-month follow-up; no attrition reported. | **Low** **risk**. Tooth movement was measured with a digital calliper by one operator; blinding of the assessor is not mentioned, but objective millimeter measurements minimize detection bias. Pain was self-reported with a visual scale—appropriate and identical across groups. | **Some concerns:** No pre-registration or protocol is cited; selective reporting cannot be excluded (e.g. cytokine data mentioned in intro were not analyzed). | **Some concerns:**  The study is judged to raise some concerns risk because two of the domains got this result |  |
|  | **Zheng et al, 2021** | **Some concerns:** The study used a split-mouth design with random assignment of laser vs. control side via a coin toss, but did not detail how the sequence was generated beyond the simple coin method nor whether allocation was concealed. | **Low** **risk**. Patients and operator both wore protective glasses; interventions followed strictly by same operator across groups, minimizing deviations. | **Low** **risk**. All 12 patients completed the study without missing data | **Low** **risk**. Outcomes (tooth movement via intraoral scanner and cytokines via ELISA) were objectively measured and analyzed by same blinded operator | **Some concerns:** No pre-registration of protocol or statistical analysis plan reported, raising some concerns for selective reporting. | **Some concerns:**  The study is judged to raise some concerns risk because two of the domains got this result |  |
| Accelerating Orthodontic Treatment Using BES | **Barsi et al, 2023** | **Some concerns:** Allocation was concealed with sealed, numbered opaque envelopes prepared by an independent person, and groups were balanced at baseline. However, the article does not describe the method used to generate the random sequence (e.g., computer list, random‐number table) | **Low** **risk.** The trial was described as double-blind with a sham mouthpiece; the treating orthodontist and participants were masked, limiting performance bias. Any researcher who delivered the current (or sham) treatment was separate from the outcome assessors. | **Low** **risk.** Figure 1 flow chart shows 28 patients randomized and the same 28 analyzed at 3 months; no withdrawals or exclusions were reported, giving low risk from attrition bias. | **Low** **risk.** Primary and secondary outcomes were either objective (caliper-measured crowding, biomarker assays) or self-reported pain on VAS. The crowding assessor was blinded and calibrated; laboratory assays are unlikely to be influenced by knowledge of allocation | **Low** **risk.** The trial lists two prospective registrations (U1111-1241-5429; RBR-7PJMJ4) and reports all outcomes described in the methods section; no unreported outcomes or selective analyses were identified | **Some concerns:**  The study is judged to raise some concerns risk because one of the domains got this result |  |
|  | **Bhad et al, 2022** | **Some concerns:** Split-mouth design with sealed-envelope allocation, but the article does not describe who generated the sequence or how envelopes were prepared | **Some concerns:** Patients and operator were aware which quadrant received PEMF; no sham device and nightly wear averaged 8 h, so behaviour could differ between sides. Authors analyzed all teeth as randomized (split-mouth ITT), tempering but not eliminating bias | **Low** **risk**. N = 19; no drop-outs or lost models reported, and both quadrants of every participant were analyzed → low risk. | **Low** **risk**. Canine–molar distances taken on plaster models by a blinded assessor using a digital calliper (ICC ≈ 0.96; Dahlberg error ≤ 0.05 mm) → low risk. | **Some concerns:** Trial was not prospectively registered and no publicly available protocol/statistical analysis plan; several outcomes (M1 vs M2 rates) presented post-hoc → some concerns. | **Some concerns:**  The study is judged to raise some concerns risk because three of the domains got this result |  |
|  | **Kim et al, 2008** | **High risk:** no statement of random-sequence generation or allocation concealment, making prognostic imbalance likely. | **Some concerns:** The micro-electric appliance had to be activated 5 h/day, but adherence verification and blinding of patients/clinicians were not reported → possible, though unquantified, deviations. | **Low** **risk**. Weekly outcome data are presented for all 7 participants; no losses or exclusions are mentioned, indicating complete data. | **Some concerns:** Tooth movement was measured with an electronic caliper each week, yet the assessor was not blinded, and measurements are operator-dependent, introducing some detection bias. | **Some concerns:** No protocol or registry is cited; only favorable outcomes are reported, so selective reporting cannot be excluded. | **High risk:**  The study is judged to raise high risk because one of the domains got this result |  |
|  | **Showkatbakhsh et al, 2010** | **Some concerns:** Split-mouth allocation (“one side randomly assigned”) is mentioned, but no description of sequence generation or allocation concealment | **Some concerns:** Participants knew a battery was embedded in the plate; adherence (8 h/night) was self-reported and not objectively verified; no evidence of differential care between sides | **Low** **risk**. All 10 participants/20 canines analyzed; no attrition reported | **High risk:** Distances were measured with digital calipers on study casts by the investigators; no blinding of outcome assessors reported | **Some concerns:** Trial was registered neither prospectively nor retrospectively; only one outcome (space closure) reported, so selective-reporting can’t be ruled out | **High risk:**  The study is judged to raise high risk because one of the domains got this result |  |
