# Supplementary figures and images for: The Effectiveness of Physical Adjunctive Interventions in the Acceleration of Orthodontic Tooth Movement: An Umbrella Review and Meta‐Analysis
Source: Int J Dent. 2026 Feb 3;2026:9131541. doi: 10.1155/ijod/9131541 (PMC12868923; doi:10.1155/ijod/9131541)

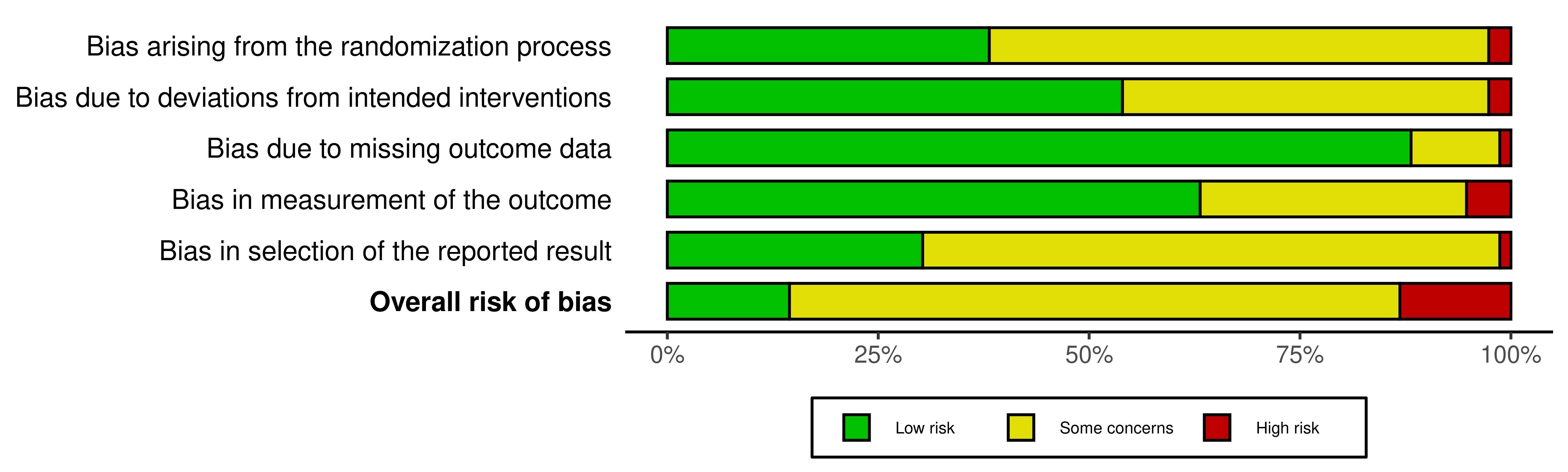

Supplement: Supplementary file 15 — Supporting Information 15 Figure S2: Overall reassessment of risk of bias profile for RCTs: Reviewers’ RoB 2 judgments presented as percentages across all included studies. [file IJOD-2026-9131541-s009.tiff]
